# Oxo-Replaced Polyoxometalates: There Is More than Oxygen

**DOI:** 10.1021/acsorginorgau.2c00014

**Published:** 2022-09-20

**Authors:** Joscha Breibeck, Nadiia I. Gumerova, Annette Rompel

**Affiliations:** Universität Wien, Fakultät für Chemie, Institut für Biophysikalische Chemie, Josef-Holaubek-Platz 2, 1090 Wien, Austria

**Keywords:** molecular metal oxides, oxygen substitution, terminal oxo-sites, bridging oxo-sites, organic functionalization, isolobal principle, charge density control, Lindqvist polyoxomolybdate, organoimido derivatives

## Abstract

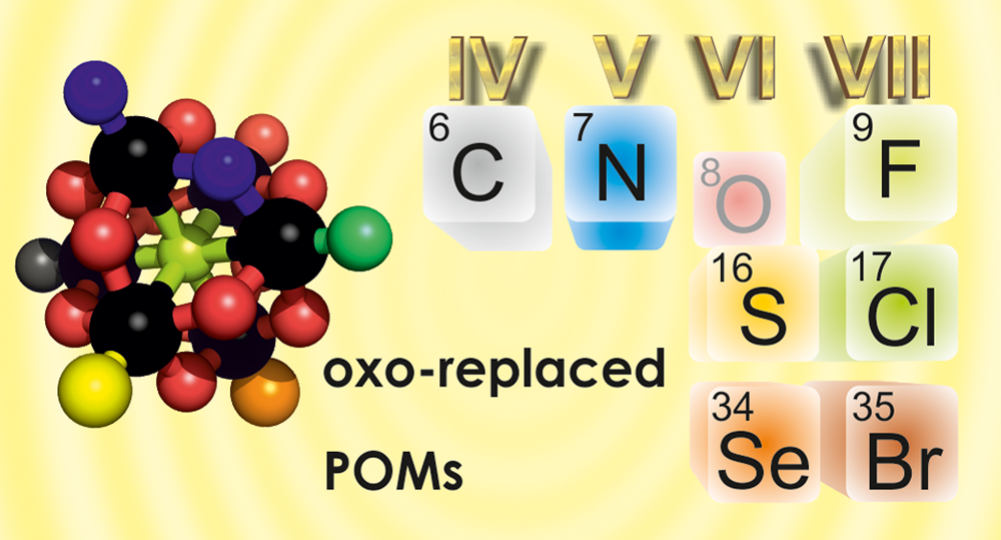

The presence of oxo-ligands is one of the main required characteristics for polyoxometalates (POMs), although some oxygen ions in a metallic environment can be replaced by other nonmetals, while maintaining the POM structure. The replacement of oxo-ligands offers a valuable approach to tune the charge distribution and connected properties like reducibility and hydrolytic stability of POMs for the development of tailored compounds. By assessing the reported catalytic and biological applications and connecting them to POM structures, the present review provides a guideline for synthetic approaches and aims to stimulate further applications where the oxo-replaced compounds are superior to their oxo-analogues. Oxo-replacement in POMs deserves more attention as a valuable tool to form chemically activated precursors for the synthesis of novel structures or to upgrade established structures with extraordinary properties for challenging applications.

## 1 Introduction

### 1.1 The Role of Oxo-Replaced Polyanions among Polyoxometalates

Polyoxometalates (POMs) are discrete polynuclear metal-oxo compounds comprising early transition metals in usually high oxidation states and demonstrate an enormous variety in archetypical scaffolds.^1,2^ POMs can be tuned in their structures and as consequence charge densities (ratio of overall anion charge *q* to number of addenda atoms *n*), which allows for highly diverse applications that include catalysis,^3,4^ bio- and nanotechnology,^5^ medicine,^6−8^ macromolecular crystallography,^9,10^ electrochemistry,^11,12^ material sciences,^13^ and molecular magnetism.^14^ There are three main structural requirements for metal-oxide to be classified as a POM: (1) the addenda ions (commonly Mo^VI^, W^VI^) have a quasi-octahedral coordination and form d_π_–p_π_ bonds with oxygens; (2) octahedra {MO_6_} (M is addenda ion) are connected via sharing a corner, an edge or rarely facet; and (3) each octahedral unit has no more than two terminal O centers.^1^

**Figure 1:**
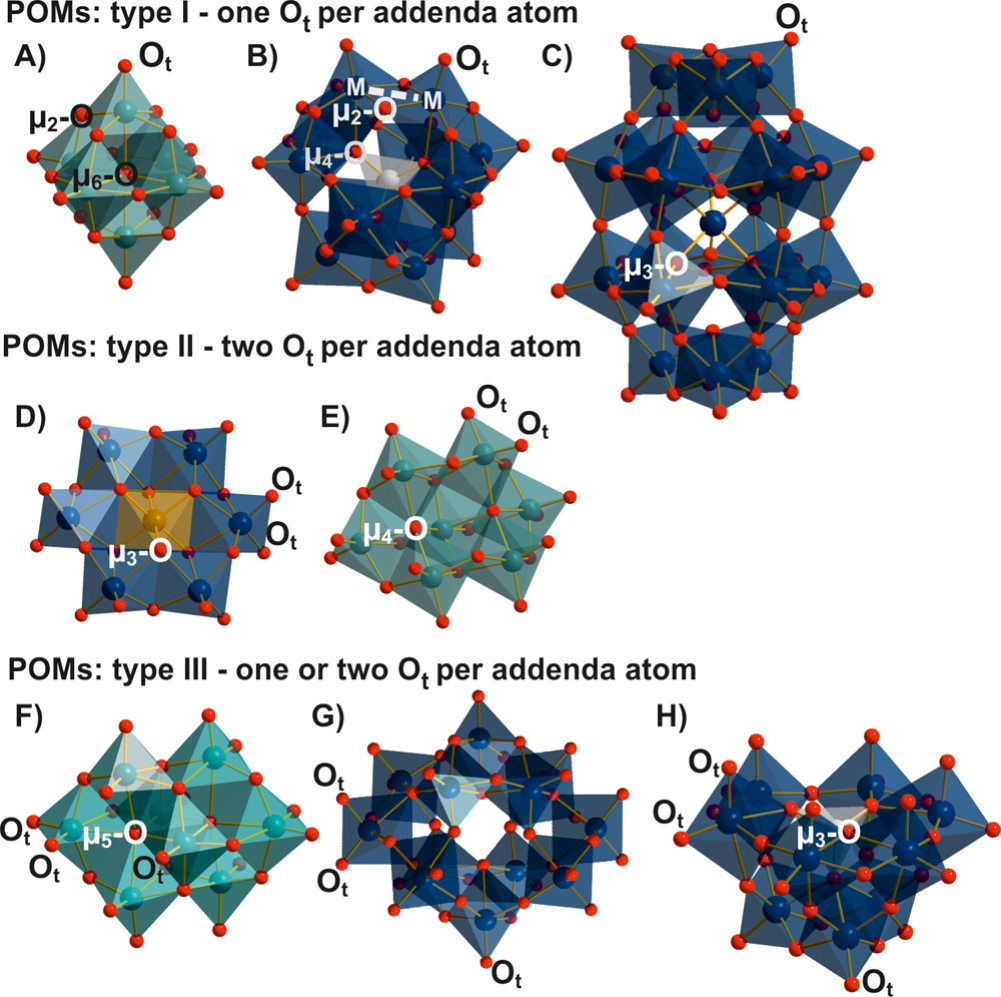
POM archetypes in a mixed ball-and-stick and polyhedral representation with a focus on structures relevant for oxo-replacement in POMs: (A) Lindqvist anion [Mo^VI^_6_O_19_]^2–^^27^ containing six O_t_, one μ_6_-O, and 12 μ_2_-O; (B) Keggin anion γ-[Si^IV^W^VI^_12_O_40_]^4–^^28^ containing 12 O_t_, four μ_4_-O, four μ_3_-O, and 20 μ_2_-O; (C) Wells–Dawson anion α-[{W^VI^O_6_}(H_2_)_2_W^VI^_18_O_56_]^6–^^30^ containing 18 O_t_, eight μ_3_-O and 36 μ_2_-O; (D) Anderson–Evans anion [Te^VI^W^VI^_6_O_24_]^6–^^32^ 12 O_t_, six μ_3_-O and six μ_2_-O; (E) heptamolybdate [Mo^VI^_7_O_24_]^6–^^33^ containing 12 O_t_, six μ_3_-O, and six μ_2_-O; (F) octamolybdate β-[Mo^VI^_8_O_26_]^4–^^34^ containing 14 O_t_, two μ_5_-O, four μ_3_-O, and six μ_2_-O; (G) paratungstate [H_2_W^VI^_12_O_42_]^10–^^35^ containing 18 O_t_, six μ_3_-O, and 18 μ_2_-O; (H) type I-derived lacunary anion *A-β-*[Si^IV^W^VI^_9_O_34_]^10–^^36^ containing 15 O_t_, four μ_3_-O, and 15 μ_2_-O. Hydrogen atoms are omitted. Color code: dark green, Mo^VI^; dark blue, W^VI^; brown, Te^VI^; light gray, Si^IV^; orange, V^V^; red, O.

The present work focuses on POM oxo-analogues, where structures were confirmed by single-crystal X-ray diffraction analysis (accessed by https://www.ccdc.cam.ac.uk/, 283 structures as of November 2021), including 99 relevant compounds with no available crystal structure, but a convincing structural characterization using, e.g., IR, powder X-ray diffraction, heteronuclear NMR, elemental analysis, and/or mass spectrometry. Only structures with direct M–X bonds of an O-replacing element X to a classical constitutional addenda ion M = V^V/IV/III^, Mo^VI/V^, W^VI/V^ are considered, starting from an addenda ion count of at least four (Figure 1). If the synthesis procedure included the addition of a lacunary POM ligand to an addenda ion complex containing a preformed M–X bond and resulting in the classical POM structure, we also consider such compounds to be oxo-substituted POMs. This is a justified approach both for the systematization of all structures with ligands other than oxygen attached to addenda ions and for considering only the final synthesized POM structures, regardless of its synthetic route. To enable understanding of the effects caused by oxo-replacement, we focus on corresponding POM structures with a verified fully O-based analogue, generally synthesized from ortho- and meta-oxometalates through condensation reactions.^1^

The oxo-substitution can lead to novel photo- and electrochemical properties compared to the unmodified POMs. Thus, a few examples of catalytic applications were reported,^15,16^ and some hybrids show promising application as conducting^17,18^ and energy storage^19^ materials. Fundamental aspects of choosing a ligand for oxygen replacement, as well as a systematic analysis of the properties of synthesized hybrid POMs presented in this review, will make an important contribution to understanding the prospects of POM modification and motivate researchers to expand existing POM classes by selecting other nonmetals as alternatives to O centers. To elucidate the impact of O-substitution on the structure, properties, and applications brought to the POM scaffold^20^ and to facilitate the selection of a suitable POM for such functionalization,^21−26^ this review summarizes for the first time decades of efforts in POM research on oxo-replaced polyanions in which at least one bridging or terminal O center has been replaced by another nonmetal element.

### 1.2 Binding Modes of Oxygen Centers within a POM Scaffold

Terminal and bridging oxygen ions are two fundamentally different classes of O centers that can be distinguished in POM structures.^1^ The terminal oxygens O_t_ are linked to only one addenda metal by a strong multiple bond, which is usually described as a double bond, although in fact three orbitals (one s and two p) are involved in its formation, and this bond can be considered as a triple bond^1^ (section 1.2.1). According to the coordination environments of the addenda ion, all POMs can be divided into three groups. In so-called type I represented by the Lindqvist^27^ (Figure 1A), Keggin^28,29^ (Figure 1B), and Wells–Dawson^30,31^ (Figure 1C), each addenda ion has just one terminal oxo ligand. Type II polyanions feature two terminal oxo ligands per addendum ion, represented by Anderson–Evans^32^ (Figure 1D) and heptamolybdate^33^ (Figure 1E) structures. Type III POMs have a combination of these two sites and are represented by octamolybdate^34^ (Figure 1F), paratungstate^35^ (Figure 1G), and various lacunary structures^36^ (Figure 1H) obtained by stepwise removal of M=O units from intact type I anions.^37^ The O_t_ sites of the resulting lacunary POMs are very reactive toward condensation or recomplementation with metals because of their strong basicity and nucleophilicity. In contrast, O_t_ centers in intact POM structures are relatively inert, as reflected in their low basicity.

Depending on the POM archetype, bridging oxygen ions interconnect the metal centers in various modes: μ_2_-O, μ_3_-O, μ_4_-O, μ_5_-O, and μ_6_-O (Figure 1). The O basicity increases in the same order. Both μ_2_-O and μ_3_-O are often sterically accessible on the surface of POM frameworks.^38^ However, the tetrahedral μ_4_-O (Keggin archetype, Figure 1B) and the octahedral μ_6_-O coordination mode of oxygen (Lindqvist, Figure 1A, and related decametalate structures) is characterized by very long and weak bonds that can only be stabilized in the center of POM scaffolds, where these highly basic oxide anions are kept inaccessible to the solvent and to other potential reagents, rendering them practically not reactive.

#### 1.2.1 Terminal Oxygen Centers and Their Replacement

**Figure 2:**
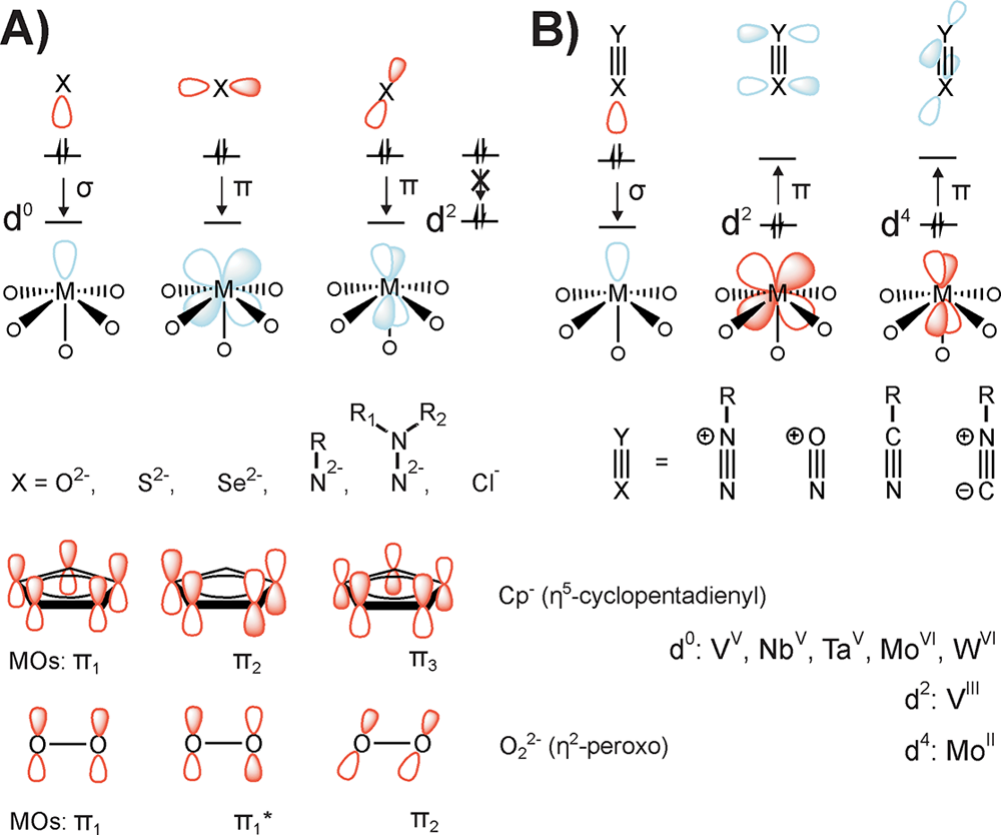
Simplified representation of the orbital interaction of ligands that occupy O_t_ sites in the binding environment of a POM addenda center M. Arrows indicate the electron donation from occupied (red) to empty (blue) orbitals. Besides the σ-bond along the binding axis, the terminal oxo site is characterized by two π-interactions. Oxo-replacing ligands are indicated by X and X≡Y. (A) Multiple binding-mode of six-electron donor ligands. In the d^2^-state (here d_*xz*_ and d_*yz*_ orbitals have been chosen to represent *t*_2*g*_ state), only one π-interaction is possible. Ligands of the first row form bonds to the metal by their p orbitals. η^5^-Cp^–^ and η^2^-O_2_ use suitable isolobal MOs with bonding (π_1_, π_2_, π_3_) or antibonding (π_1_*) character. (B) Formal binding situation with reduced metal centers and electron-accepting terminal ligands. Up to two π-bonds are formed by back-donation from the addenda center to empty π*-orbitals of the ligand.

Because of the good steric accessibility, the replacement of terminal oxygen centers O_t_ is by far more frequent and easier to accomplish than that of bridging oxygens in the intact POM structures. O_t_ substitution reactions in the intact anions remain, at least theoretically, unaffected by the rest of the energetically stabilized POM framework and should therefore require a lower activation barrier to occur. The binding interaction of O_t_ centers with the d^0^ addenda metal (e.g., fully oxidized W^VI^, Mo^VI^, or V^V^) in POMs results in a six-electron donation (one σ and two π d–p bonds) to the metal center (Figure 2A).^39^ According to the isolobal principle,^40^ which describes the analogy of electronically equivalent and energetically similar frontier orbitals, other ligands that donate six electrons for metal bonding and match the symmetry and size of this metal interaction site should be able to replace the terminal oxo group. Indeed, several POM compounds (Table S1) were prepared with sulfido (S^2–^, section 4.1), selenido (Se^2–^, section 4.2), imido (NR^2–^, section 3.1.2), hydrazido (N-NR_2_^2–^, section 3.1.3), chlorido (Cl^–^, section 5.2), and even cyclopentadienido (η^5^-C_5_H_5_^–^) or η^2^-peroxido (η^2^-O_2_^2–^) ligands (see section 2.1) with analogue electron structure (Figure 2A). The local reduction of d^0^ addenda metal centers with removal of the terminal oxido ligand to d^2^ (e.g., the diazenido (N=NR^–^), section 3.1.4, nitroso (N=O^–^), section 3.1.6), or even d^4^ (e.g., nitrilo (N≡CR) and isonitrilo (C≡NR), section 3.1.7) state fills the previously empty electron-accepting d orbitals and allows an entirely different interaction with ligands demonstrating π-acceptor character (Figure 2B). The metal d-electrons then stabilize the bond to the ligand through back-donation.

#### 1.2.2 Bridging Oxygen Centers and Their Replacement

Bridging oxygens are more nucleophilic and basic, but because they are less sterically accessible (especially μ_3_, μ_4_, μ_5_, or μ_6_) than O_t_ sites, they are less likely to be substituted. With their electron lone pairs, the μ_2_- and μ_3_-O centers (Figure 1) participate in multicenter bonds that stabilize the whole POM framework,^41^ making them difficult to extract without destroying the polyanion structure. So far, there are only three examples of μ-O substitutions in POM structures (see section 3.2).^42^

### 1.3 Synthetic Approaches and Challenges

The elements suitable for O replacement, which are identified and analyzed in this work, cover a defined Pauling electronegativity (EN) window between 2.55 (C, Se) and 3.98 (F). The chemical similarity between F and O allows for the substitution of multiply bridging μ_3_-O in Keggin structures and even μ_6_-O sites in the center of Lindqvist structure by direct incorporation of F^–^ during synthesis from HF.^43^ The replacement of O centers within a POM scaffold by other nonmetal elements, usually with lower Pauling EN than the one for oxygen (3.44), brings up several synthetic challenges. The precursors used for another nonmetal element transfer are often very reactive and need to be protected from oxidation or hydrolysis in a chemically inert atmosphere, i.e., degassed organic solvent, leaving the classical aqueous POM chemistry environment behind. Similarly, the resulting M–X bonds (M, addenda ion; X, O-replacing ligand) in the substituted POMs have a different polarity, and consequently reactivity compared to the M-O bonds, and sometimes require a protecting environment to prevent bond hydrolysis or oxidation. Due to the high redox activity of POMs, one should be aware that the POM scaffold itself can oxidize certain substituting residues (e.g., primary hydrazides). Generally, those ligands best mimicking the electronic interactions at oxo-sites are most stably incorporated into POM frameworks.

Based on the POM archetype and their size, three synthetic approaches can be applied. In small condensed anions like the Lindqvist anion (Figure 1A), O_t_ centers can be activated for extraction from the POM scaffold by suitable reagents (e.g., carbodiimides)^44^ and efficiently substituted by alternative moieties like imido (NR^2–^) or hydrazido (N-NR_2_^2–^) groups (see section 3.1.1). This works especially well for polyoxomolybdates (POMos), as the W=O groups of polyoxotungstates (POTs) are less reactive. All other larger anions show a lower reactivity of the O_t_ sites and therefore require another synthetic strategy based on the combination of preformed M–X-containing (M, addenda ion; X, O-replacing ligand) fragments with lacunarized POMs. The incorporation of preassembled M–X is the most promising and extendable approach circumventing the sterical shielding.^45^ This synthetic approach is a valuable route to otherwise inaccessible structural features in Keggin anions like diazenido (N=NR)^46^ or nitroso (N=O)^47^ functions. In a third less frequently used approach, the desired functionalization is obtained by direct co-assembly of ortho-metalate monomers M^VI^O_4_^2–^ (M = Mo, W) in the presence of suitable precursor building-blocks (e.g., [W^II^(NO)Cl_3_(CH_3_CN)_2_] as a source for {W^II^(NO)}^3+^^48^).

## 2 Oxo-Replacement by Group 14 Elements: Carbon

Carbon (Pauling EN: 2.55) is the only element of group 14 known so far substituting oxygen in POM structures (Table S1). Carbon-based ligands have been shown to replace one^49^ or two^50^ terminal O_t_ sites in the Lindqvist archetype (Figure 1A), but with completely different underlying chemical principles as purely electron-donating or π-accepting ligands. So far, there are no reports on the replacement of oxygen by carbon for other POM archetypes.

### 2.1 η^5^-Cyclopentadienyl Stabilization by Lindqvist POM Anions

**Figure 3:**
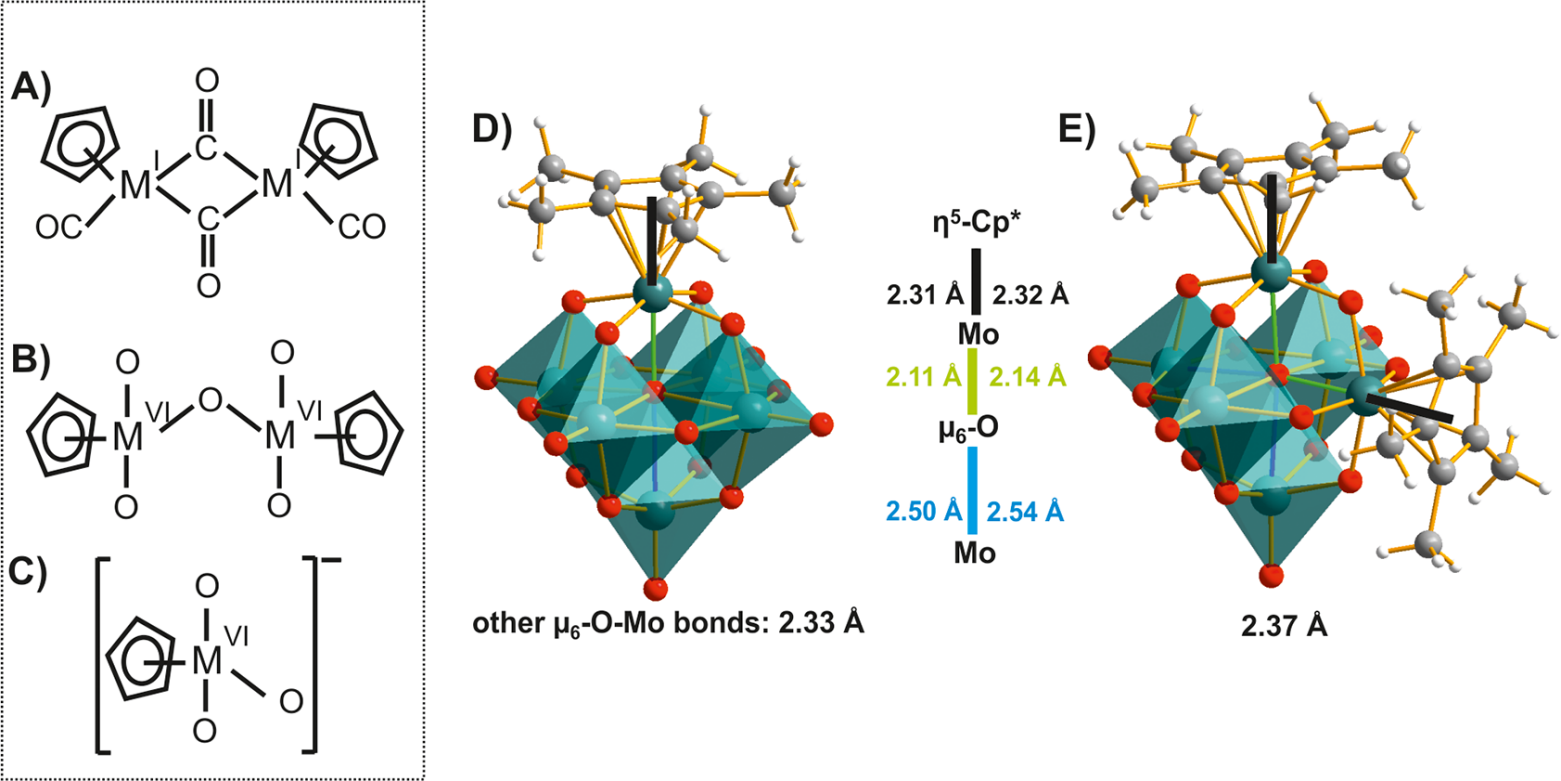
Precursors^53^ (A–C) used for the synthesis of η^5^-Cp*-substituted Lindqvist POMs. (D) [Mo^VI^_6_O_18_(η^5^-Cp*)]^−^^49^ and (E) *cis*-[Mo^VI^_6_O_17_(η^5^-Cp*)_2_]^0^ ^50^ as examples of mono- and disubstitution. In precursors formula M stands for Mo or W. The relevant bonds are highlighted in color, and their lengths are compared. For the η^5^-Cp* ligand, the bond distance to the ligand center of gravity is measured. Color code: dark green, Mo^VI^; red, O; dark gray, C; white, H.

The π-system of the monovalent η^5^-pentamethylcyclopentadienyl anion (Cp^*–^, C_5_H_5_^–^) is isoelectronic and isolobal^40^ to the O_t_ group (Figure 2A). Although some Mo^VI/V^ and W^VI/V^ complexes with Cp^*–^ can decompose spontaneously in moist air,^51^ O_t_ substitution by Cp^*–^ is achieved in water/methanol solutions.^52^ The assembly of dinuclear metal–organic precursor [(η^5^-Cp*)W^I^(CO)_2_]_2_^53^ (Figure 3A) in a water/methanol mixture directly yielded the oxidized disubstituted Lindqvist POT *cis*-[W^VI^_6_O_17_(η^5^-Cp*)_2_]^52^ (isostructural Mo-analogue in Figure 3E). The same synthesis strategy with [(η^5^-Cp*)Mo^I^(CO)_2_]_2_ (Figure 3B) was applied to give [Mo^VI^_6_O_18_(η^5^-Cp*)]^−^ (Figure 3D).^54^ Other organo-precursors as [((η^5^-Cp*)M^VI^)_2_O_5_]^2–^ (M = Mo, W) (Figure 3B) or [(η^5^-Cp*)Mo^VI^O_3_]^−^ (Figure 3C) were applied to obtain mono-^49^ (Figure 3 D) and difunctionalized structures^50,55,56^ (Figure 3 E).

In [Mo^VI^_6_O_18_(η^5^-Cp*)] (Figure 3D),^49^ the Mo-Cp* bond (2.31 Å) is significantly longer than the O_t_ bond (1.68 Å) that it replaces. The phenomena that the central oxygen atom μ_6_-O largely moves to the Mo ion bearing the Cp* ligand (Figure 3D) and that additional surface charges probably extend to the terminal oxygen atoms in *trans*-position in anions Cp*Mo_6_ and Cp*_2_Mo_6_ are explained by *trans*-influence.^57^ Wang et al. explained this phenomenon based on the energetic level and character of the involved molecular orbitals (MOs).^57^ Due to the large EN difference between the metal center and the O_t_ ligand, the bonding orbitals are strongly polarized toward the O center, leaving little electron density at the metal and thereby reducing the *trans*-interaction with the μ_6_-O. With decreasing EN difference by replacing O_t_ (EN: 3.44) with an N-based ligand (EN: 3.04; see section 3) or even carbon (EN: 2.55), the respective bonding MOs exhibit a reduced ligand character and a more pronounced metal character with more electron density available for interaction with the *trans*-μ_6_-O-ligand. The system can be stabilized by strengthening this *trans*-interaction with the more electronegative element and shortening the respective bond to O. This phenomenon also explains why the even more deformed disubstituted *cis*-isomer (Figure 3E) is strongly favored over the symmetric *trans*-isomer (not displayed in Figure 3), which does not allow bond stabilization. A decrease in charge density upon Cp*^–^ replacement of O leads to an increased reducibility of the POM anions, as confirmed by cyclovoltammetry (CV).^55^ So far, for Cp*-substituted Lindqvist POMs, no application has been reported.

### 2.2 Isocyanide in Reduced Vanadium Anions: Modeling the Carbonyl Interaction

In 2019, Matson et al. showed a way to functionalize reduced V^V/IV^-based Lindqvist POMs with one or two isocyanide groups (C≡N–R) replacing O_t_ sites.^58^ The removal of one or two O_t_ in [V^V^_2_V^IV^_2_O_7_(OCH_3_)_12_]^0^ reveals reduced V^III^ at the surface of the POM, which can react with *tert*-butyl isocyanide. Isocyanide features a dual ligand behavior as strong σ-donor and π-acceptor that only can bind to reduced V^III^ addenda ions providing d-electrons for π-back-bonding (Figure 2B). Matson et al. showed that the attachment of *tetra*-butyl isocyanide, a carbon monoxide analogue, provides insight into the ability of the vacant reduced Lindqvist polyoxovanadates (POVs) to activate CO in order to mediate the emission of this toxic environmental contaminant.^58^

## 3 Oxo-Replacement by Group 15 Elements: Nitrogen

The vast majority of ligands substituting for O in POM structures feature an N (Pauling EN: 3.04) donor, the only stable O substitute from group 15 in the periodic table. The rich chemistry of nitrogen makes it possible to interact both with fully oxidized and with reduced addenda centers at O_t_ sites, and even bridging O centers can occasionally be replaced. The type II and III POM structures can be functionalized by weakly bound N ligands (see info about amino, imino and amido ligands in the SI), but multiply bonded N ligands show a higher application potential due to their complex electronic structure and are discussed in this section.

### 3.1 Substitution of Terminal Oxygens

#### 3.1.1 Nitrido Functionalization

The simplest nitrogen-based ligand to replace O_t_ groups is the more highly charged nitrido (N^3–^) function. Applied synthetic approaches to introduce this function into a common heteroatom such as Mo^VI^ or W^VI^ have not been reported so far. Although it was once claimed that the nitrido derivative of Lindqvist hexamolybdate could be obtained by assembly of appropriate Mo building blocks,^59^ no crystallographic data are available. POTs and POMos with direct nitrido-functionalization remain the subject of theoretical studies, which predict that they are stable and behave as nucleophiles toward electrophilic reagents.^60^

#### 3.1.2 Imido Functionalization: The Most Applied Route to Hybrid Oxo-Replaced Structures

##### 3.1.2.1 The Lindqvist-Type

**Scheme 1:**
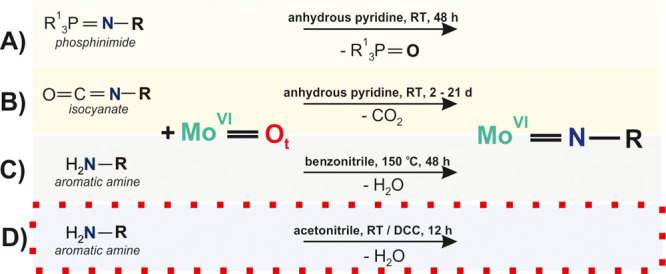
Synthesis of Organoimido Lindqvist-Type POMos [Mo^VI^_6_O_19–*x*_(NR)_*x*_]^2–^ Four methods (A–D)^44,62−65^ for O_t_ substitution are shown with the most efficient protocol^44^ highlighted (red dotted line). The synthetic strategy (D) was developed by Wei^44^ based on the first report of an organoimido derivative obtained from a reaction with an aromatic amine.^65^ The {Mo^VI^=O} fragment is shown without POM scaffold. DCC, dicyclohexyl carbodiimide, R and R^1^, aromatic group.

Imido ligands R–N^2–^ are suitable alternatives to oxo groups in POM structures with an equivalent binding behavior (Figure 2A) that do not overload POM addenda with additional charge. Almost all POM imido derivatives comprise Lindqvist-type POMos [Mo^VI^_6_O_19–*x*_(NR)_*x*_]^2–^ (*x* = 1–6), and it is synthetically feasible to replace even all six O_t_ sites in this anion.^61^ The first applied imido transfer agents were phosphinimides R^1^_3_P=N–R^2^ (R^1^, R^2^ = aryl) with sufficient reactivity in pyridine solvent to be used in equimolar amounts in 48 h (Scheme 1A).^62^ Later, the milder isocyanates R–N=C=O (Scheme 1B) were used in high excess as more reactive alkyl ligands for POMo imido-functionalization; however, the reaction took several days.^63,64^ Finally, Wei et al. established a reliable and efficient protocol (Scheme 1C,D) to carry out the substitution reaction with amines R–NH_2_ in 48 h in benzonitrile or even in 12 h in acetonitrile using a stoichiometric amount of the dehydrating agent dicyclohexyl carbodiimide (DCC), which even works for less reactive aromatic amines.^44^

**Figure 4:**
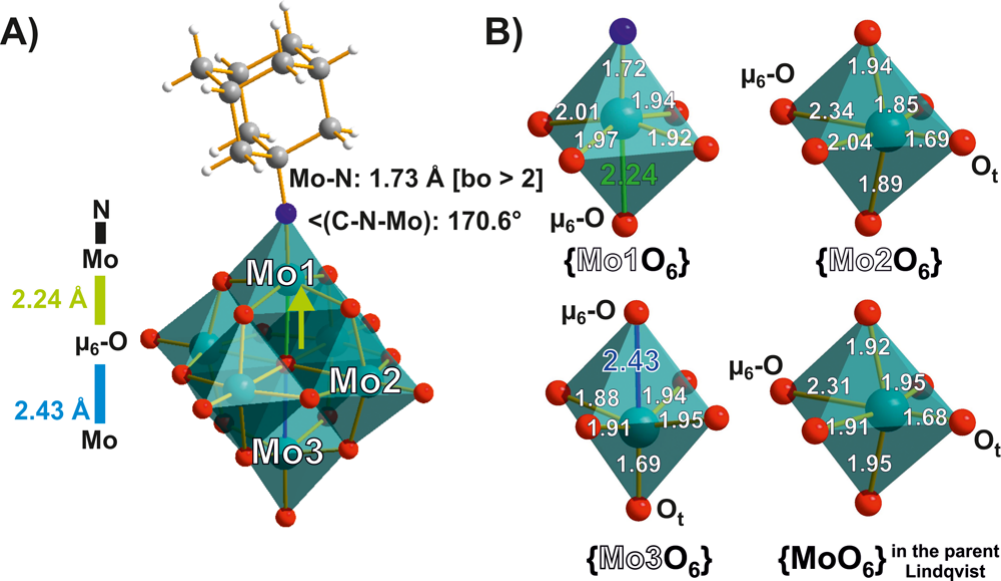
(A) The adamantyl-imido(N–C_10_H_15_)-functionalized Lindqvist anion [Mo^VI^_6_O_18_(N–C_10_H_15_)]^2–^.^66^ (B) Bond lengths in Å for the {MoO_6_} octahedra in the axial (Mo1 and Mo3) and equatorial (Mo2) positions compared to the bond lengths for {MoO_6_} in the parent Lindqvist POMo. Color code: dark green, Mo; red, O; blue, N; dark gray, C; white, H.

**Figure 5:**
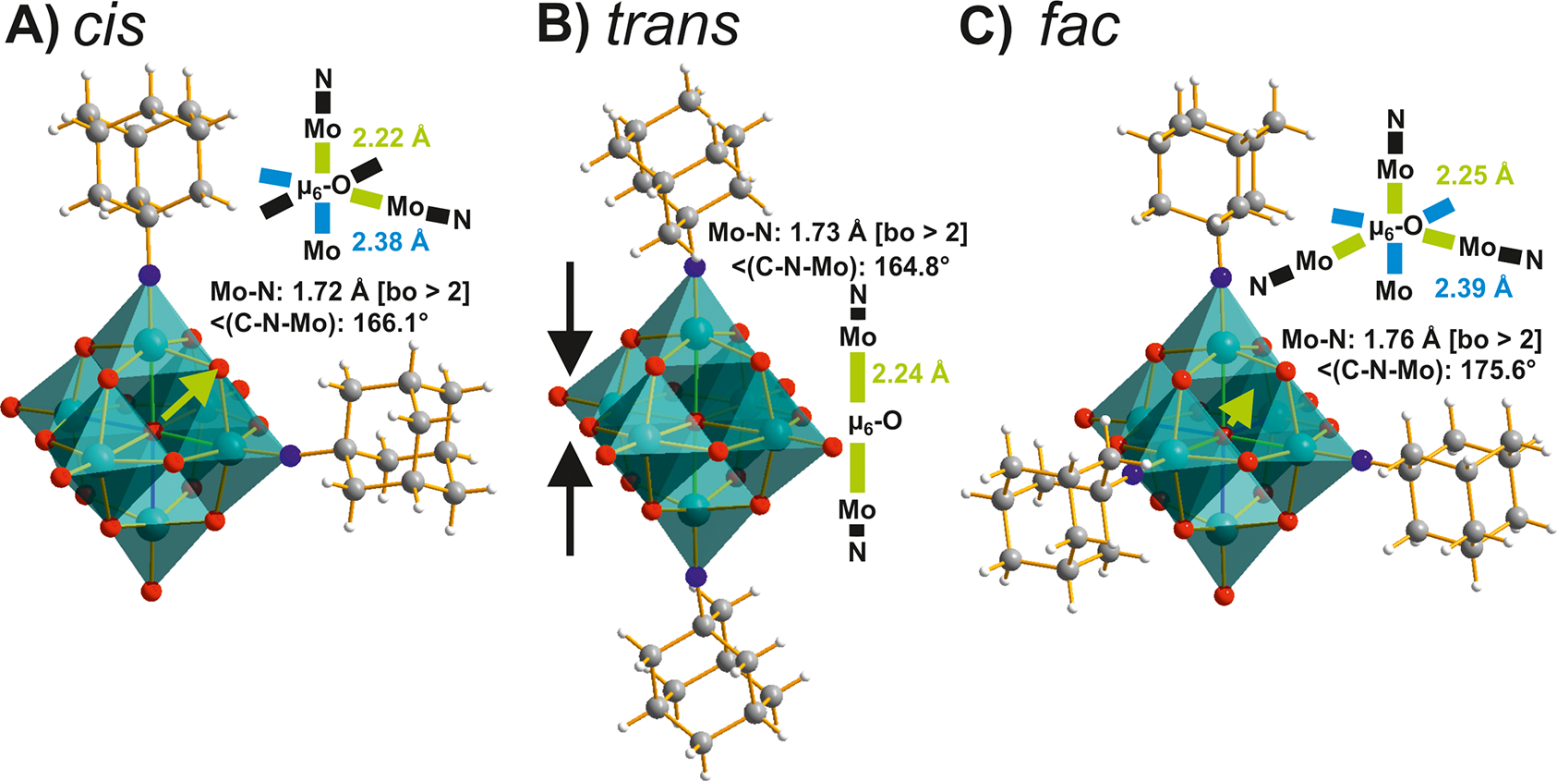
Homologous adamantyl-imido(N–C_10_H_15_)-functionalized Lindqvist POMos in a mixed ball-and-stick and polyhedral representation. (A) *cis-*[Mo^VI^_6_O_17_(N–C_10_H_15_)_2_]^2–^ with neighboring substituents,^66^ (B) *trans-*[Mo^VI^_6_O_17_(N–C_10_H_15_)_2_]^2–^ with opposite substituents,^66^ and (C) *fac-*[Mo^VI^_6_O_16_(N–C_10_H_15_)_3_]^2–^^66^ with three substituents as part of the same superoctahedral face. The relevant bonds are highlighted in color, and their lengths are compared. Color code: dark green, Mo^VI^; red, O; blue, N; dark gray, C; white, H.

Substitution of terminal oxygens alters the *trans*-influence (cf. Section 2.1 for explanation) as well, which is manifested in different structural consequences for mono-, di-, and trifunctionalized Lindqvist anions (Figures 4 and 5).^26,66^ The μ_6_-O displacement (Figures 1A, 4, and 5) is only slightly pronounced (with Mo−μ_6_-O distances 2.43 Å for the unsubstituted site and 2.24 Å for substituted (Figure 4A)), in accordance with the strong imido–metal triple-bond character. With the exception of the substituted sites, the remaining octahedra in the monosubstituted structure have a geometry close to that of the parent Lindqvist anion (Figure 4B), while in the di- and trifunctionalized Lindqvist anions, the distortion is very close to that of the unsubstituted Lindqvist POMo. Nevertheless, the *cis*- (Figure 5A) and *fac*-substitution (Figure 5C) patterns (23 and 2 structures, Table S1) are clearly favored over the *trans*- (Figure 5B) (5 structures, Table S1) and the not yet obtained *mer*-isomers. The Mo−μ_6_-O distances are almost the same for *cis*- (Figure 5A) and *fac*-isomers (Figure 5C). The *trans*-substitution appears to be kinetically favored and can be achieved under controlled reflux conditions at 95 °C.^67^ Strong et al.^63^ showed by CV that imido-POMs are less easily reduced, which is linearly related with increasing degree of substitution, demonstrating the more pronounced electron-donating effect of the imido-group compared to the oxo-form.

The first mixed-addenda structures of arylimido(NR)-Lindqvist POMs [W^VI^_5_O_18_{Mo^VI^(NR)}]^2–^^68^ (R is 2,6-dimethylaniline C_6_H_3_(CH_3_)_2_NH_2_ or 2,6-dimethyl-4-iodoaniline C_6_H_2_I(CH_3_)_2_NH_2_) were obtained by selective O_t_ replacement at the Mo^VI^ center, underlining the distinct reactivity of the Mo^VI^=O_t_ group. Wei et al. showed that, compared to the hexamolybdate anion, [Mo^VI^W^VI^_5_O_19_]^2–^ shows slightly less reactivity, and the reaction is further complicated by the competing conversion of [Mo^VI^W^VI^_5_O_19_]^2–^ to [W^VI^_6_O_19_]^2–^.^68^ Because of the relatively low reactivity of the W=O groups, there is only one example of an imido-Lindqvist POT prepared using an isocyanate precursor and a reaction time of 7 days.^69^

Several Lindqvist^70^ or Anderson–Evans^71−73^ POM structures have been functionalized with tris(hydroxymethyl)aminomethane (TRIS) and further conjugated via an imido bond Mo=N–R. Remarkably, a one-pot reaction of octamolybdate α-[Mo^VI^_8_O_26_]^4–^, Mn^III^ and TRIS under microwave conditions leads to the *in situ* conversion of one polyanion precursor to two different archetypes, followed by their controlled coupling [Mo^VI^_6_O_18_NC(OCH_2_)_3_Mn^III^Mo^VI^_6_O_18_(OCH_2_)_3_CNMo^VI^_6_O_18_]^7–^.^71^

##### 3.1.2.2 Potential Application of Organoimido Lindqvist POMos

Theoretical^74−76^ and experimental^77−79^ works show that imido-functionalized POMs demonstrate second-order nonlinear optical (NLO) coefficients (first hyperpolarizability, β > 10^–27^ esu in some cases) that compete with high-performance organic materials,^80^ and is based on POM to ligand charge transfer. Fielden and co-authors^81−83^ recently have investigated a family of arylimido Lindqvist POMos that demonstrate hyper-Rayleigh scattering with *β*_0_-values of up to 133 × 10^–30^ esu exceeding those of any dipolar organic system with comparable donor, π-system and absorption profile. Given the relatively high energies of organoimido-POM electronic transitions and the challenge of obtaining high-activity molecular NLO materials with adequate transparency (reabsorption of visible light can cause lowered efficiency, overheating and instability), the arylimido Lindqvist anions can be viewed as a platform for new highly active and transparent, second-order NLO materials that have a potential application in telecommunications.

Organo-imido hexamolybdates conjugated with poly(phenylene-ethynylene) polymers have been used in photovoltaic cells and as components in dye-sensitized solar cells.^84−86^ Due to their ability to accept electrons, POMs can successfully replace other electron acceptors, such as fullerenes, introduced to provide charge separation from photogenerated excitons. These results convincingly demonstrate the potential application of oxo-replaced POM-based organo-inorganic hybrids in molecular electronics and photonics.

Another distinctive feature of the organoimido Lindqvist POMos is the propensity for self-assembly in solutions and formation of nanoscaled paddle-wheel complexes and blackberry-type assemblies.^87−89^ The use of amphiphilic oxo-replaced POM hybrids is a feasible and effective approach with more controllability and designability to fabricate POM-nanostructures.

Four arylamido-functionalized Lindqvist POMos [Mo^VI^_6_O_18_(≡NAr)]^2–^ have been tested in DMSO solution against human leucocythemia K562 tumor cells and showed lower inhibitory activities (growth inhibition percentage up to 53.4% at POMo concentration 100 μg/mL) than the antitumor drug of clinical practice 5-fluorouracil (62.2%), but better inhibitory activities than that of parent POMo (Bu_4_N)_2_[Mo^VI^_6_O_19_] (28.7%) at the same concentration.^90,91^ The adamantyl-imido (N–C_10_H_15_)-substituted hexamolybdate (Figure 5C) has promising antiproliferative performance on breast cancer MCF-7 cells in mixed DMSO-medium solvent compared with unfunctionalized hexamolybdate and amantadine (1-adamantylamine, C_10_H_15_–NH_2_), which shows antiproliferative activity itself.^66^ Finally, organoimido Lindqvist POMo functionalized with *N*-acylureido and 2-amino-3-methylbenzoxyl (C_8_H_11_NO) group exhibits favorable pharmacodynamics toward human malignant glioma cell (U251) and the ability to penetrate across the blood–brain barrier and low toxicity toward rat pheochromocytoma cells (PC12).^92^ Although organoimido Lindqvist POMos showed promising biological activity, attention should be given to their low solubility in aqueous solutions and the need to add an organic solvent such as DMSO for dissolution.

The preliminary herbicidal activity test indicated that fluoro-functionalized phenylimido hexamolybdates display potent herbicidal activity, in particular against the roots of some tested plants (such as *Brassica campestris L.*, *Eclipta prostrata L.*, *Echinochloa crusgallis L.*, and *Cirsium japonicum DC.*).^93,94^

##### 3.1.2.3 The Keggin-Type

The O_t_ centers of the Keggin archetype are less reactive than their Lindqvist counterparts and cannot be substituted in the intact anion by a condensation reaction.^1,39^ The only organoimido-substituted Keggin derivative is the phenylimido POT [P^V^W^VI^_12_O_39_(N–C_6_H_5_)]^3–^, obtained by recomplementation of the lacunary anion [P^V^W^VI^_11_O_39_]^7–^ with a preformed imido tungsten chloride precursor W^VI^(NC_6_H_5_)Cl_4_.^95^ The structure was confirmed by ^1^H, ^31^P, ^183^W, and ^1^H–^183^W HMQC NMR spectroscopy as well as cyclic voltammetry, electronic absorption, and elemental analysis. Density functional theory (DFT) calculations suggest a similar *trans*-influence (cf. Section 2.1 for Lindqvist POMo) of the imido-linkage on the bond to the central μ_3_-O (Figure 1B) in [P^V^W^VI^_12_O_39_(N–C_6_H_5_)]^3–^. In another theoretical study on two Keggin [P^V^W^VI^_12_O_39_(N–C_6_H_5_)]^3–^, [P^V^Mo^VI^_12_O_39_(N–C_6_H_5_)]^3–^ and one Lindqvist [Mo^VI^_6_O_18_(N–C_6_H_5_)]^2–^, it was shown that phenylimido group effectively modifies the electronic properties and Keggin POMo has the strongest oxidation abilities within this series.^96^

#### 3.1.3 Hydrazido-Functionalization: Mimicking Carbonyl Chemistry

**Figure 6:**
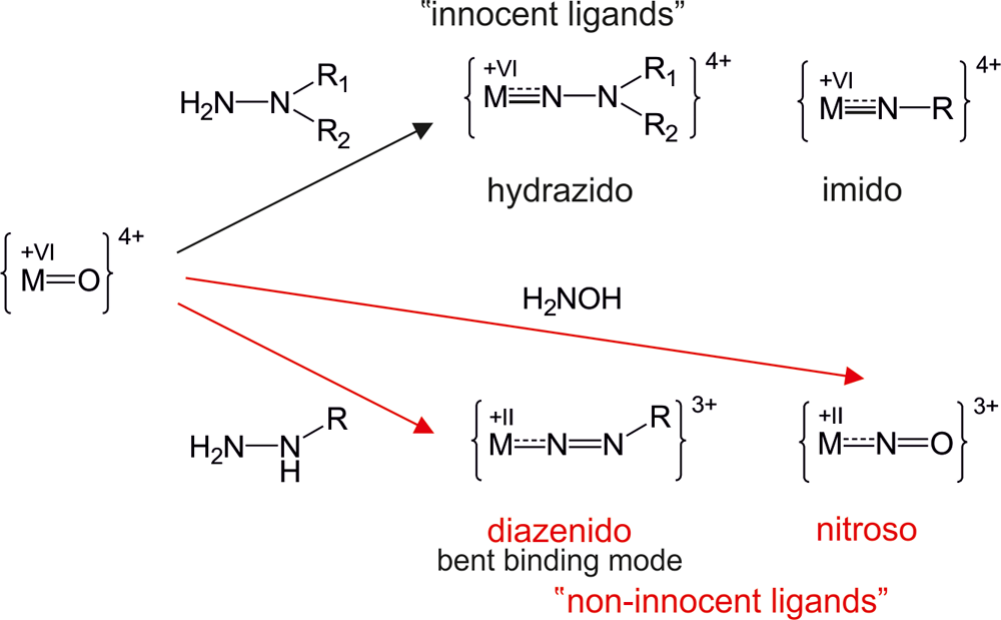
Reaction of “innocent” (no redox reaction, black) and “non-innocent ligands” (redox reaction involved, red) with addenda centers M^VI^ (M = Mo, W) according to the common formalism. The hydrazido and diazenido-functionalization has only been reliably reported with M = Mo. 1,1-Disubstituted hydrazines form a triple bond similar to imido-functionalization. Monosubstituted hydrazines, however, are oxidized upon attachment to the metal center, reducing it to the M^II^ state. Thereby, the nature of the ligand and the binding interactions are significantly changed (cf. Figure 2B). Hydroxylamine performs a similar reaction yielding nitroso-functionalization.

All organic reactions of amines with carbonyls and carboxylates are also suitable for hydrazides (H_2_N-NR_2_). A hydrazido linkage (N-NR_2_^2–^) can only be achieved with a 1,1-disubstituted hydrazine (H_2_N-NR_2_), in a condensation reaction as in case of imido (NR^2–^) conjugation (Figure 6).

**Figure 7:**
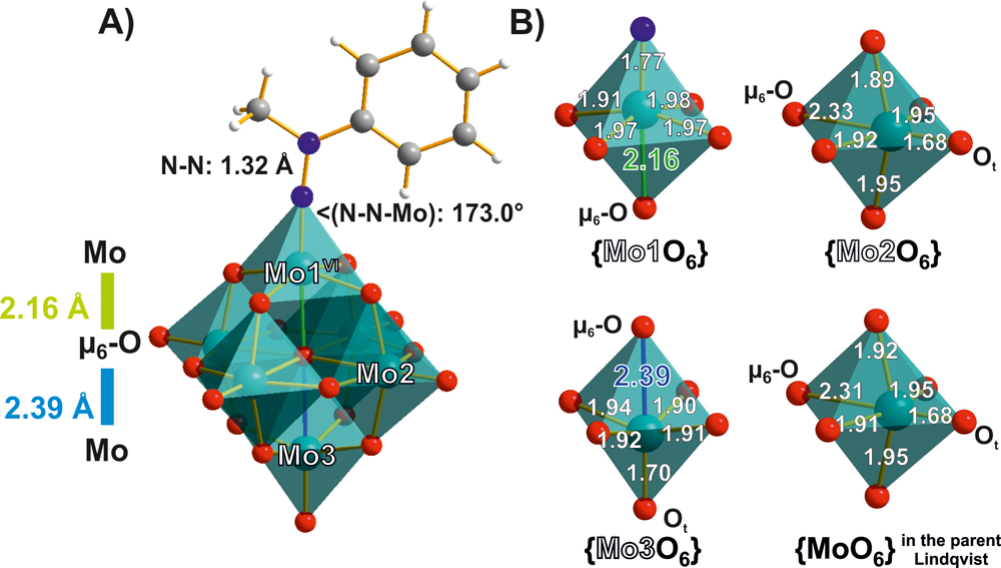
(A) The hydrazido-functionalized [Mo^VI^_5_Mo^II^O_18_(NN(CH_3_)(C_5_H_6_))]^2–^.^59^ (B) Bond lengths in Å for the {MoO_6_} octahedra in the axial (Mo1 and Mo3) and equatorial (Mo2) positions compared to the bond lengths for {MoO_6_} in the parent Lindqvist POMo. Color code: dark green, Mo; red, O; blue, N; dark gray, C; white, H.

The only reported structure is a 1-methyl-1-phenyl hydrazine-substituted Lindqvist hexamolybdate (Figure 7A),^59^ which actually compares well with an imido derivative in its structural and binding properties (Figure 2A). Monosubstitution leads to a larger distortion of {MoO_6_} in the axial position and does not affect the distortion in equatorial octahedra (Figure 7B). The 1,2-disubstitution pattern does not lead to stable POM attachment, and monosubstituted hydrazines react in a markedly different way (see section 3.1.4). So far, no properties have been identified for the hydrazido-functional POMs that could lead to applications.

#### 3.1.4 Diazenido Ligands: “Non-Innocent” Ligand Behavior

Diazenido groups (−N=N–R) are formed when primary hydrazines are used for O_t_ functionalization (Figure 6). The addenda metal center is reduced to its d^4^ state by the ligand, which corresponds to M^II^ for Mo or W, showing so-called “non-innocent” behavior (Figure 6). In this case, the binding situation can be imagined as a σ-donation of a diazonium ligand to the metal and a π-backdonation to the ligand (Figure 2B). This is in accordance with the usually observed bent-diazenido binding-mode (Figure 6).^97^

##### 3.1.4.1 The Lindqvist-Type

**Figure 8:**
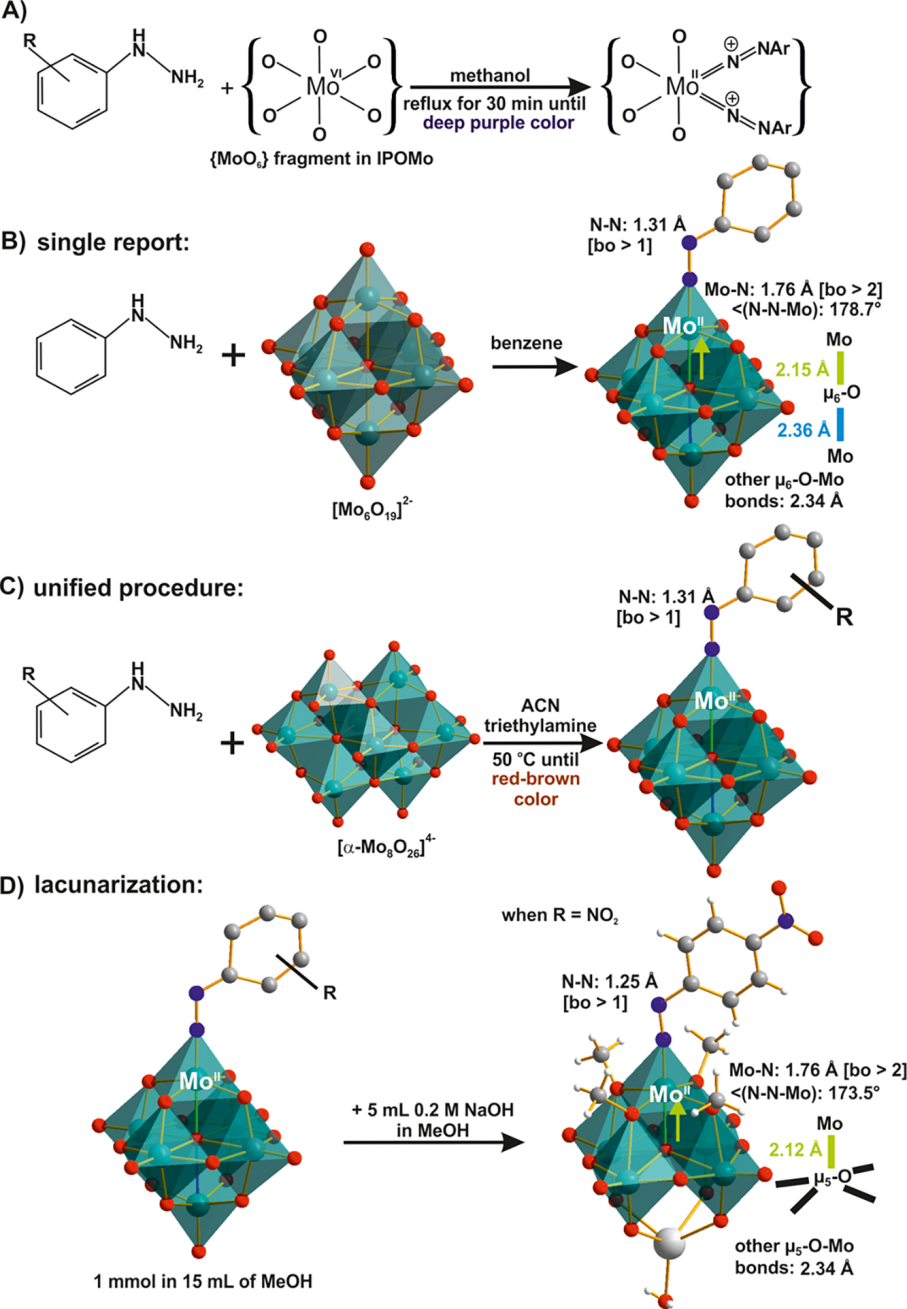
Hydrazido and diazenido-functionalization. (A) Reaction of {MoO}_6_ unit in isopolyoxomolybdate (IPOMo) with arylhydrazine. (B) Synthesis of [Mo^VI^_5_Mo^II^O_17_(NN-C_5_H_6_)]^3–^.^99^ (C) Synthesis of [Mo^VI^_5_Mo^II^O_18_(NN-C_5_H_4_-R)]^3–^.^100^ (D) Formation of [Mo^VI^_4_Mo^II^O_13_(OCH_3_)_4_(NN-C_6_H_4_NO_2_){Na(H_2_O)}]^2–^.^46^ The relevant bonds are highlighted in color and their lengths are compared. Color code: dark green, Mo; red, O; blue, N; dark gray, C; white, H; light gray, Na.

To date, only POMos have been functionalized with diazenido groups (Figure 8). The products of the reactions between arylhydrazines and polyoxomolybdates in alcohols mostly contain [Mo^II^(N_2_Ar)_2_]^2+^ units (Figure 8A), in which Mo^II^ is bonded to two ligands and can only form low-nuclear anions such as [Mo^II^_4_O_8_(OCH_3_)_2_(NNAr)_4_]^2–^.^98^ The derivatives containing [Mo^II^(N_2_Ar)]^3+^ units are not readily available, and Hsieh and Zubieta^99^ reported the first compound prepared from phenylhydrazine and Lindqvist [Mo^VI^_6_O_19_]^2–^ (Figure 1A) in dry benzene (Figure 8B). Later Gouzerh et al.^100^ showed that the synthesis of [Mo^VI^_5_Mo^II^O_18_(N_2_Ar)]^3–^ depends crucially on the temperature and the composition of the reactant mixture, while the type of solvent is negligible. During the synthesis, heating at 50 °C should be stopped as soon as the color of the mixture has changed to reddish brown and the addition of triethylamine is favorable for the reaction (Figure 8C). For possible applications, it should be noted that [Mo^VI^_5_Mo^II^O_18_(N_2_Ar)]^3–^ anions are only moderately stable in solution and are mainly converted into [Mo^VI^_6_O_19_]^2–^ in DMF or to α-[Mo^VI^_8_O_26_]^4–^ in acetonitrile even at 50 °C.^100^ As many hydrazines have interesting biological activities (e.g., antidepressant^101^ or anticancer^102^), their introduction through covalent bonding can confer biological properties on Lindqvist POMo. Benzoyldiazenido-functionalized hexamolybdates exhibit enhanced antitumor inhibitory activities against human leucocythemia K562 cells compared to the activity of hexamolybdate and the corresponding hydrazine precursors alone.^103^

In addition to the intact Lindqvist anion, the monolacunary compounds with the diazenido modification can also be synthesized (Figure 8D) by controlled basic hydrolysis of the parent POM in methanol in the presence of NaOH.^46^ Remarkably, the diazenido bond withstands such harsh conditions breaking the POMo integrity. The modified lacunary fragments [Mo^VI^_4_Mo^II^O_13_(OMe)_4_(NNAr){Na(MeOH)}]^2–^ were applied to form sandwiches with main group (Ba^III^ and Bi^III^)^46^ and lanthanide (Tb^III^, Dy^III^, Ho^III^, Er^III^, Yb^III^, and Nd^III^)^104^ cations.

Proust and Coronado showed that the lanthanide-based sandwiches {LnMo_10_} demonstrate single-ion magnet (SIM) behavior and are soluble in organic solvents, making them easier to process and incorporate into spintronic devices.^104^ These polyoxomolybdate-based SIMs can facilitate their processability due to the presence of organic groups by being grafted onto surfaces/electrodes or by allowing the incorporation of another property via the organic ligand.^104^

**Figure 9:**
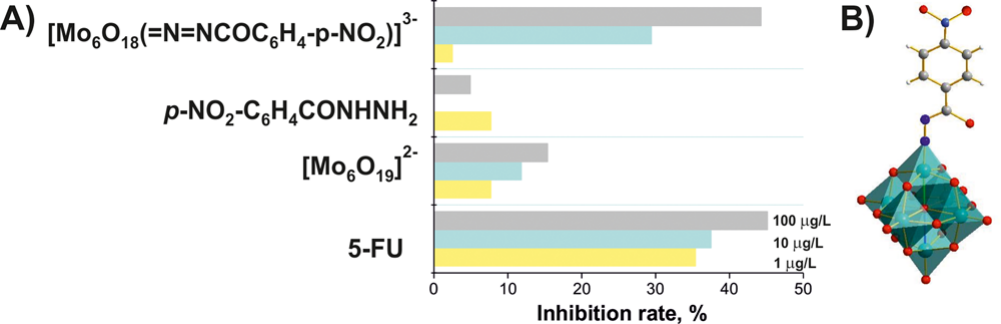
Antitumor activity tests against human leucocythemia K562 tumor cells. (A) Inhibitory rate for three reference compounds 5-fluorouracil (5-FU), [Mo^VI^_6_O_19_]^2–^, *p*-nitrobenzoylhydrazine *p*-NO_2_-C_6_H_4_CONHNH_2_, and corresponding hybrid compound dissolved in DMSO with concentrations of 1, 10, and 100 μg/mL; (B) [Mo^VI^_5_Mo^II^O_18_(NN-CO(C_6_H_4_–*p*-NO_2_))]^3–^.^105^ Color code: dark green, Mo; red, O; blue, N; dark gray, C; white, H.

In analogy to arylamido-functionalized Lindqvist POMos (see Section 3.1.3), antitumor activity tests against K562 show that most of the benzoyldiazenido-functionalized Lindqvist POMos have enhanced inhibitory activities compared to hexamolybdate and the corresponding hydrazide ligands (Figure 9).^105^

##### 3.1.4.2 The Keggin-Type

The [Mo^II^(NNR)]^3+^ fragment was generated *in situ* from the lacunary Lindqvist POMo in a acetonitrile(ACN)/methanol mixture and transferred to a lacunary Keggin POT [P^V^W^VI^_11_O_39_]^7–^ to generate the first diazenido-derivative of this archetype.^46^

#### 3.1.5 Diazoalkane and Thiosemicarbazide: Between Hydrazido and Diazenido Bonding

##### 3.1.5.1 The Lindqvist POMo

**Figure 10:**
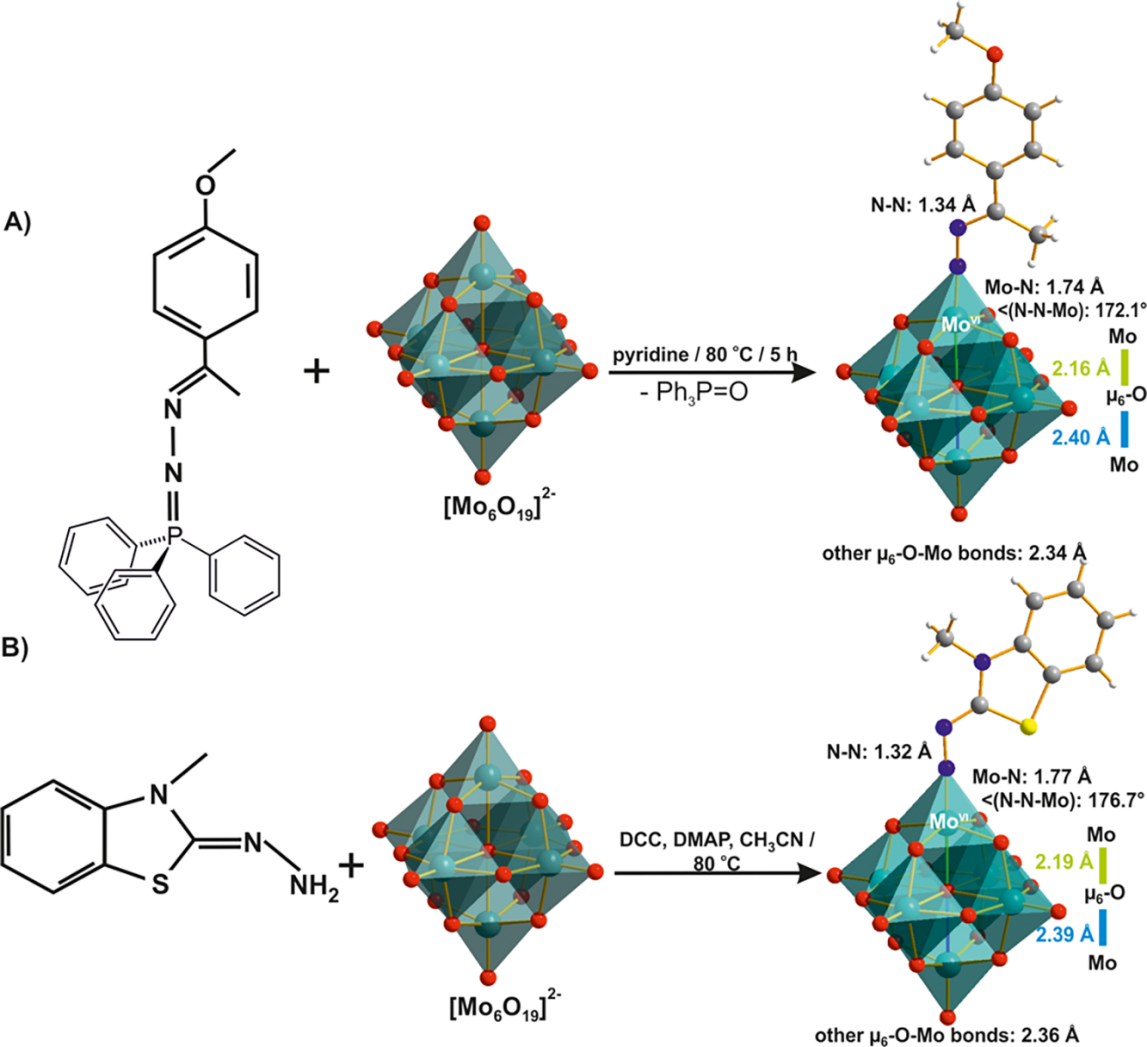
Synthesis scheme of: (A) [Mo^VI^_6_O_18_(NN-C(CH_3_)(C_6_H_4_OCH_3_))]^2–^^106^ and (B) [Mo^VI^_6_O_18_(NN-C_8_H_7_SN)]^2–^.^107^ DCC, *N*,*N*′-dicyclohexylcarbodiimide; DMAP, 4-dimethylaminopyridine. The relevant bonds are highlighted in color and their lengths are compared. Color code: dark green, Mo; red, O; blue, N; yellow, S; dark gray, C; white, H.

The binding of a diazoalkane (hydrazonato) ligand (N–N=CR^1^R^2^)^−^^106^ (Figure 10A) and a thiosemicarbazide system (N–N=C(NR^1^R^2^)(SR^3^))^2–^^107^ (Figure 10B) to Mo in Lindqvist POMo occurs at a higher temperature (Figure 10) and shows intermediate features between the dinitrogen-ligand binding modes described above.

Formally, the Mo^VI^ center in question is linked to the ligand through a hydrazido bond, but also conjugated to an sp^2^-C carbon in a π-donating environment, delivering electrons to the N–N bond and reducing the formal charge of the Mo ion. The binding mode also demonstrates the efficient electron delocalization in the POM framework interfering with the formal metal oxidation states. In all compounds, the Mo–N bond has a clear triple-bond character and the N–N distance indicates a partial double bond, both based on the bond length analysis in X-ray structures.^108^ The redox properties of the benzothiazole hydrazone–hexamolybdate hybrid [Mo^VI^_6_O_18_(NN-C_8_H_7_SN)]^2–^ (Figure 10B) together with a good electronic communication between the organic π system and the molybdenum centers make these compounds very promising building blocks for conducting molecular materials.^107^

##### 3.1.5.2 The Lindqvist POT

The diazoalkane hexatungstate analogue was synthesized in a one-pot reaction from orthotungstate and a diazoalkane-transferring phosphazine due to the reduced reactivity of the POT O_t_ sites, rather than by functionalization of the intact anion as in the POMo version.^109^

#### 3.1.6 Nitroso-Functionalization: An Analogue of the Diazenido System

##### 3.1.6.1 The Lindqvist-Type

**Figure 11:**
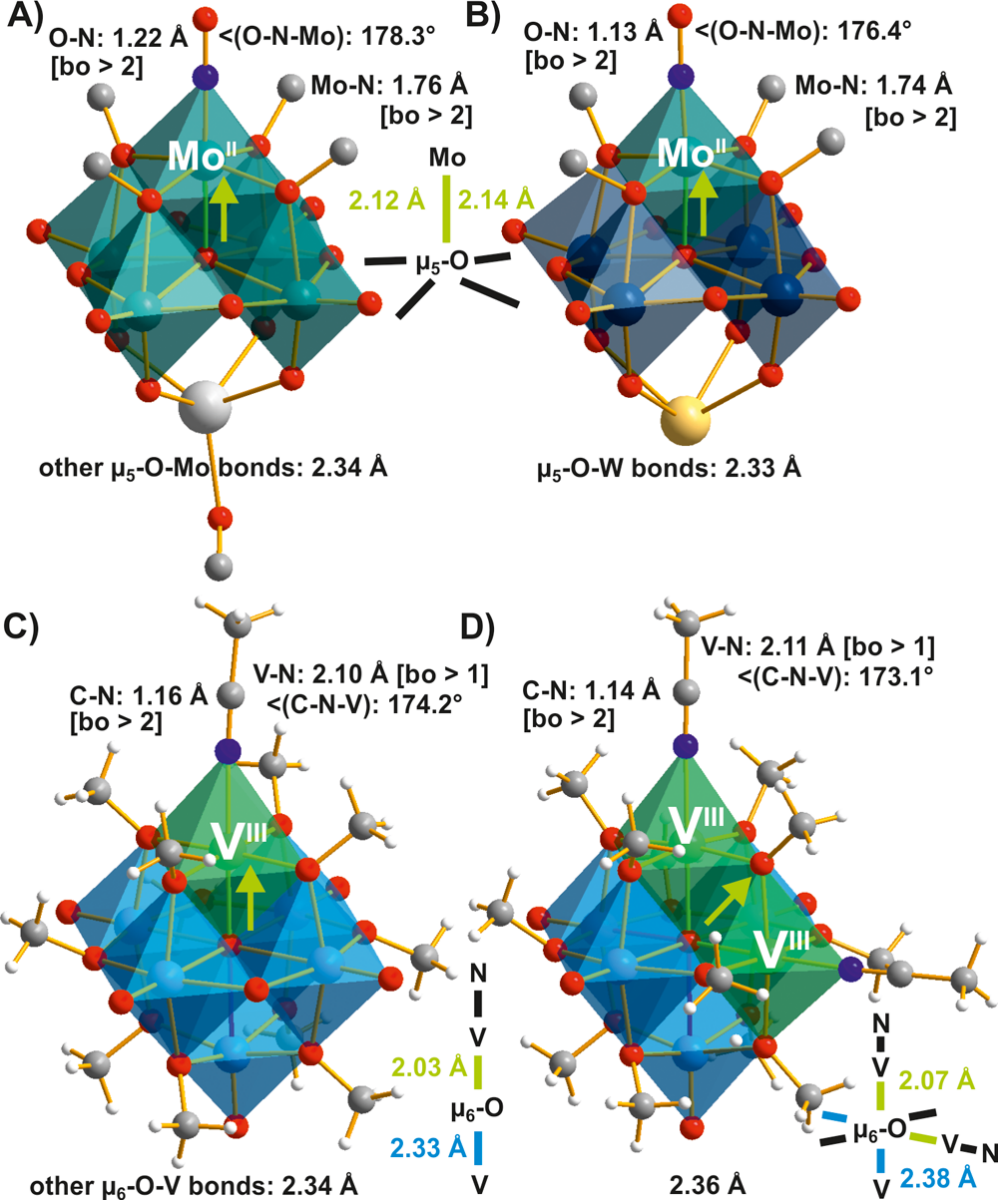
Lindqvist POMs with electron-accepting nitrogen ligands in a mixed ball-and-stick and polyhedral representation: (A) Lacunary anion [Mo^VI^_4_Mo^II^O_13_(OCH_3_)_4_(NO){Na(OCH_3_)}]^2–^,^111^ (B) lacunary fragment [W^VI^_4_O_13_(OCH_3_)_4_{Mo^II^(NO)}{Bi^III^)}],^112^ (C) neutral Lindqvist POM [V^V^V^IV^_4_V^III^(OCH_3_)_12_O_6_(NCCH_3_)]^0^ (the V^V^ and V^IV^ sites are fluctuating and not precisely located),^58^ and (D) neutral Lindqvist POM *cis-*[V^IV^_4_V^III^_2_(OCH_3_)_12_O_5_(NCCH_3_)_2_]^0^.^58^ The relevant bonds are highlighted in color and their lengths are compared. Color code: dark green, Mo; dark blue, W^VI^; light blue, V^IV^; green, V^III^; red, O; blue, N; dark gray, C; white, H; light gray, Na; yellow: Bi^III^.

The formal treatment of the diazenido ligand (N=NR)^−^ as a diazonium (N≡NR)^+^ cation facilitates the understanding of the nitrosyl binding situation as an isoelectronic and isolobal ligand interaction (Figure 2B). A nitrosyl (R–NO) function is commonly introduced into a POM framework by reacting with the reagent hydroxylamine (Figure 6), accompanied by oxidation of the ligand and reduction of the metal center to M^II^. The functionalization of Lindqvist POMos and POTs (Figure 11A,B) was developed by Proust et al.^48,110,111^ by base hydrolysis of the parent anion, similar to diazenido derivatives, demonstrating an impressive stability of the NO modification. The N–O bond distance is very short when compared to the diazenido derivatives, indicating only weak π interaction with the reduced Mo^II^ center. The reactive lacunary anions of {XM_10_} archetype were used to form sandwiches with main group (Ca^II^, Sr^II^, Ba^II^ and Bi^III^^112^), transition-metal (Ag^I^,^113^ Mn^II^ and Re^II^^114^), and lanthanide (Ce^III^ and Eu^III^;^112^ Tb^III^, Dy^III^, Ho^III^ and Er^III^^115^) cations.

The Dy-containing nitroso-functionalized POMo {Dy[Mo^VI^_4_Mo^II^O_13_(OMe)_4_(NO)]_2_}^3–^ shows slow magnetization relaxation with an energy barrier for magnetization reversal of 50 K, which is the highest barrier height observed for a polyoxomolybdates-based SIM.^115^

##### 3.1.6.2 The Keggin-Type

One lacunary compound was obtained by co-assembly of a [Mo^II^(NO)]^3+^ unit and a tungstate precursor [P^V^W^VI^_11_O_39_]^7–^,^47^ and the subtle difference in the chemical environment of the electron-withdrawing POT framework led to an even less pronounced π-backdonation from Mo^II^ to the NO ligand, as indicated by the stronger N–O multiple bond (Figure 11A,B). NO-substituted POMs are generally more reducible than their oxo-forms with the same charge density due to the electron-accepting properties of the NO ligand.^47^

#### 3.1.7 Nitrilo Ligands: Masking Reduced Metal Sites

The neutral Lindqvist hexavanadates [V^V^V^IV^_4_V^III^(OCH_3_)_12_O_6_(NCCH_3_)]^0^ and *cis-*[V^IV^_4_V^III^_2_(OCH_3_)_12_O_5_(NCCH_3_)_2_]^0^ (Figure 11C,D), with a nitrilo group (N≡C–R) coordinated to the V^III^ sites, were originally developed by the Matson group.^58^ Again, only the *cis*-isomer was obtained upon difunctionalization, and the μ_6_–O-V^III^ bonds show a significant contraction, accompanied by a relatively long bond from the addenda center to the ligand, with its C–N triple bond largely preserved. This is consistent with the observations for the nitroso ligand (N=O), which also behaves as a weak π-acceptor. These compounds inspired the use of isonitriles (C≡N–R) as analogues to C≡O, with the nitrilo group (N≡C–R) exhibiting similar π-acceptor bonding properties (Figure 2B).

### 3.2 Substitution of Bridging Oxygen Sites: A Synthetic Surprise

In principle, the bridging imido function μ-(N–R)^2–^ should be a suitable substitute for any μ_2_- or μ_3_-O centers, with the residue R sterically protecting the more reactive metal–nitrogen bonds. However, these steric hindrance effects also preclude μ_3_-O sites from replacement, and only the μ_2_-sites at the POM surface seem to be accessible for the reaction. Only the Lindqvist POMo structure shows sufficient reactivity to undergo partial μ_2_-(N–R) replacement, requiring intermediate structural dis- and reconnection steps of the POM framework.

#### 3.2.1 The Lindqvist-Type POMo

**Figure 12:**
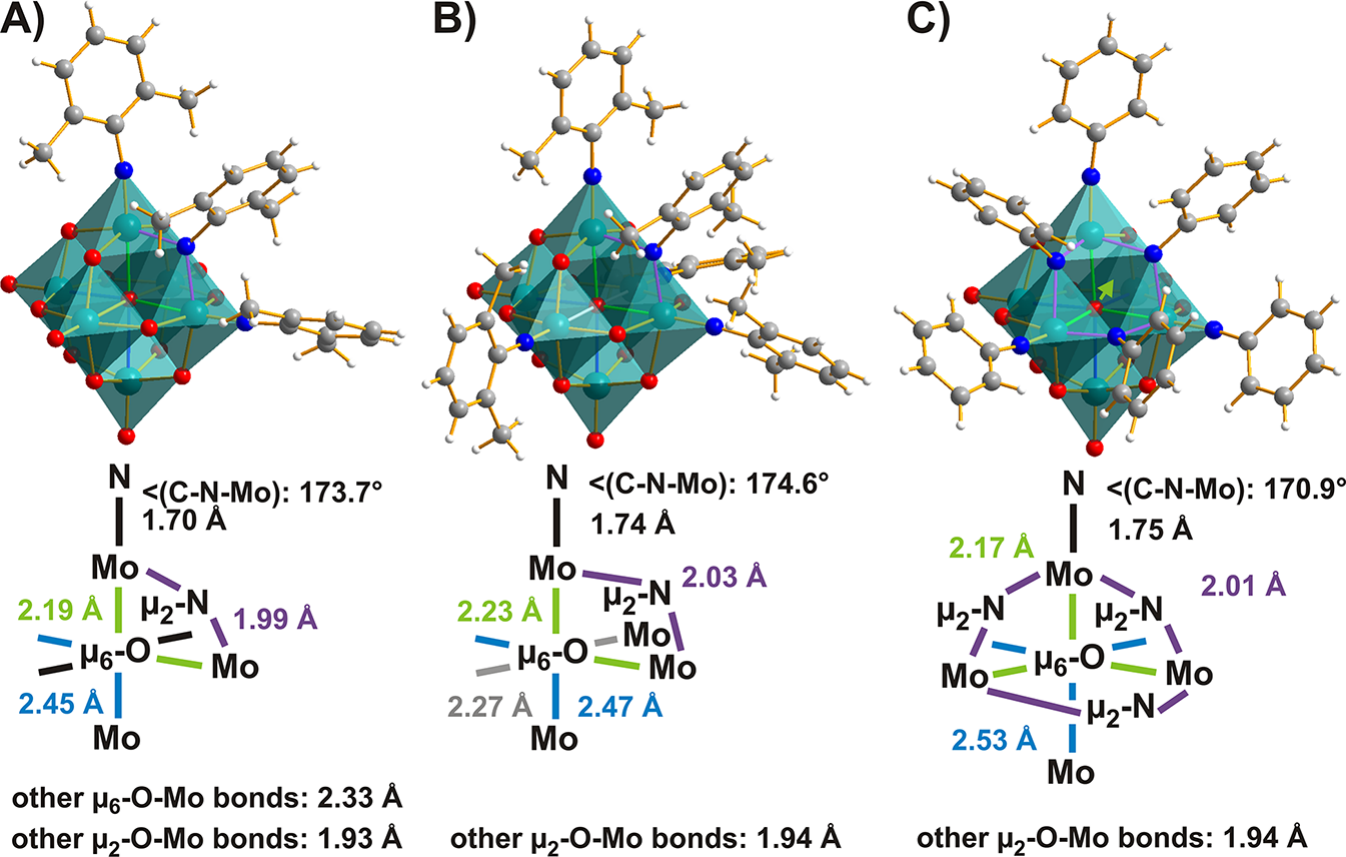
Lindqvist hexamolybdates featuring μ_2_-bridging imido ligands in a mixed ball-and-stick and polyhedral representation: (A) *cis*-[Mo^VI^_6_O_16_(NR)_2_(μ_2_-NR)]^2–^ with R = 2,6-dimethylphenyl,^42^ (B) *fac*-[Mo^VI^_6_O_14_(NR)_4_(μ_2_-NR)]^2–^ with R = 2,6-dimethylphenyl,^42^ and (C) *fac*-[Mo^VI^_6_O_13_(NR)_3_(μ_2_-NR)_3_]^2–^ with R = phenyl.^116^ The μ_2_-imido bonds to the metal centers are slightly longer than their O analogues, as they can participate with only one electron lone pair in additional metal π interaction. The relevant bonds are highlighted in color and their lengths are compared. Color code: dark green, Mo^VI^; red, O; blue, N; dark gray, C; white, H.

There are three examples for the replacement of bridging O centers in POMos (Figure 12),^42^ which were obtained applying a modified protocol for the multiple imido (=N–R^2–^) functionalization described in Section 3.1.2. This is underlined by the fact that the dimethylanilido-substituted anion with four terminal and one bridging O replacements was always obtained as a cocrystal with the only terminally modified compound. Common to all structures discussed here (Figure 12) is that only μ_2_-O positions linking two imido-Mo centers could be substituted (by the very same primary amine replacing the respective O_t_ sites), leading to an overall *cis*- or *fac*-substitution pattern. This type of substitution is preferred because the terminal imido modification activates distinct μ_2_-O atoms for reaction with the dehydration agent DCC, most likely by increasing their nucleophilicity with respect to the other bridging O sites. The structures of *cis*- and *fac*-isomers indicate a more pronounced interaction of the μ_6_-O center with the N-functionalized metal ions and presumably reduced π-involvement of the μ_2_-O position. The μ_2_-(R–N^2–^) function is well compatible with such less electron-demanding metal centers with a correspondingly longer μ_2_-N–Mo^VI^ distance (Figure 12). The only structure with multiple bridging O-replacement is a CCDC entry (1033546)^116^ with no publication linked to it.

Interestingly, a theoretical DFT study of the Lindqvist POMo and POT with varying central elements predicted the central μ_6_-nitrido group to form the shortest bonds to the addenda centers, even shorter than with a μ_6_-O.^117^ All μ_2_-O and O_t_ sites showed increased bond lengths, while all other group 5 (P and As) and group 6 (S and Se) elements were simulated to interact much weaker with the addenda metal ions.

## 4 Oxo-Replacement by Group 16 Elements: Chalcogens

Terminal oxygens O_t_ can be substituted by peroxide (see SI) and by single S^2–^ or even Se^2–^ ions, using suitable transfer agents (e.g., bis(trimethylsilyl)sulfide) or preformed fragments (e.g., {M^V^_2_S_2_O_2_}). Sulfido groups S^2–^ or organic thiols RS^–^ were not observed as bridging moieties in fully oxidized POMs, possibly because of their sensitivity to oxidation. The unique reduced building block [M^V^_2_S_2_O_2_]^2+^ (M = Mo, W),^118^ however, enables incorporation into POM frameworks and stabilizes the POM unit modifying its electronic structure.^45^ The polyoxothiometalate chemistry with particular emphasis on the reactivity of the cationic building blocks [Mo^V^_2_O_2_S_2_]^2+^ and [Mo^V^_3_S_4_]^4+^ toward POM building blocks has been carefully reviewed by Cadot et al.,^45^ and section 4.1.2 describes the most important oxo-replacing features for polyoxothiometalates.

### 4.1 Sulfur: The Higher Homologue of Oxygen

Although S parallels O in its chemical behavior and reactivity, its strongly reduced Pauling EN (2.58) and more diffuse valence orbitals hamper the interaction with POM addenda. Terminal or bridging sulfido groups are only stable when linked to a metal center with relatively weak oxidizing power (with lower standard redox potential) such as Nb^V^.

#### 4.1.1 The Terminal Sulfido Group: An Uneven Exchange for Oxygen

Klemperer et al.^119^ chose mixed-addenda Lindqvist POTs with a unique reactive O_t_ site at a Nb^V^ or Ta^V^ center and replaced it by an S^2–^ group using the S transfer agent bis(trimethylsilyl)sulfide ((CH_3_)_3_Si)_2_S in ACN. They thoroughly characterized the products [W^VI^_5_O_18_{Nb^V^S}]^3–^ and [W^VI^_5_O_18_{Ta^V^S}]^3–^ by ^17^O NMR and IR spectroscopy. Later, the Sécheresse group^120^ applied the same approach to a lacunary Keggin POT structure with the S transfer agent [2,4-bis(4-methoxyphenyl)-1,3-dithia-2,4-diphosphethane-2,4-disulfide] to obtain [P^V^W^VI^_11_O_39_{Nb^V^S}]^4–^ and analyzed the compound by IR, Raman, ^31^P NMR spectroscopy, and CV. There, the O replacement led to a slight shift of the reduction waves to a lower potential. This increase in reducibility was explained by an energy shift of the respective POT orbitals due to subtle structural changes.^120^ Radkov et al.^121^ investigated [P^V^W^VI^_11_O_39_{M^V^S}]^4–^ (M = Nb, Ta) and [W^VI^_5_O_18_{M^V^S}]^3–^ (M = Nb, Ta) by ^17^O, ^31^P, and ^183^W NMR spectroscopy and showed that the terminal S-bonds in the Keggin structures were more resistant to hydrolysis than in the Lindqvist compounds despite their overall lower charge density, which usually results in a more pronounced OH attack.

#### 4.1.2 Bridging μ_2_-Sulfido Ligands: Unique Stability as Part of the {M_2_S_2_O_2_} Subunit

##### 4.1.2.1 {M^V^_2_S_2_O_2_} Subunit

**Figure 13:**
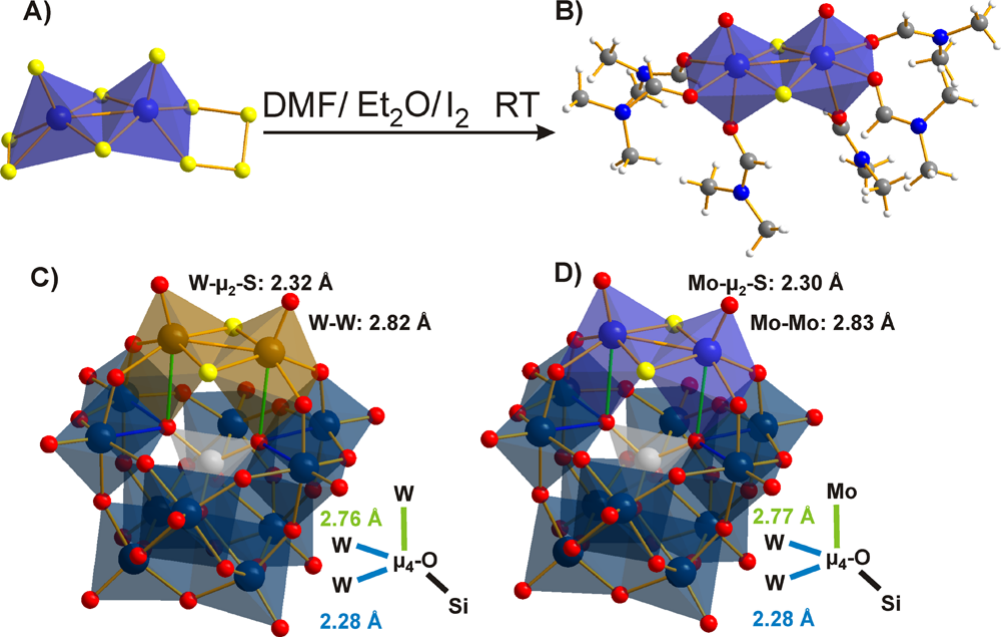
POMs with bridging sulfido ligands based on the {M^V^_2_S_2_O_2_} unit in a mixed ball-and-stick and polyhedral representation: (A) Thio-precursor [Mo^V^_2_S_2_O_2_(η^2^-S_4_)(η^2^-S_2_)]^2–^,^122^ (B) dinuclear building block [Mo^V^_2_S_2_O_2_(OCHN(CH_3_)_2_)_6_]^2+^ crystallized from DMF (dimethylformamide) with labile solvent molecules in the position of water ligands,^123^ (C) γ-[Si^IV^W^VI^_10_O_36_{W^V^_2_S_2_O_2_}]^6–^,^120^ and (D) γ-[Si^IV^W^VI^_10_O_36_{Mo^V^_2_S_2_O_2_}]^6–^.^120^ The incorporation of the reduced dinuclear unit elongates the μ_3_-O bond connecting the M^V^ centers to the central silicate. The relevant bonds are highlighted in color and their lengths are compared. Color code: dark blue, W^VI^; light blue, Mo^V^; brown, W^V^; red, O; blue, N; yellow, S; light gray, Si.

S cannot substitute for any bridging O site, but an interesting way to introduce μ_2_-S sites is by incorporation of the dinuclear {M^V^_2_S_2_O_2_} subunit (M = Mo, W) into a POM framework.^45^ Therefore, partial oxidation and hydrolysis of a thio-precursor, such as [M^V^_2_S_2_O_2_(η^2^-S_4_)(η^2^-S_2_)]^2–^ (Figure 13A) in water under S elimination,^45,122^ produces the dinuclear cationic building block [M^V^_2_S_2_O_2_(H_2_O)_6_]^2+^ (Figure 13B).^123^ During the synthesis of {M^V^_2_S_2_O_2_}, the metal centers of the precursor ortho-thiometalates M^VI^S_4_^2–^ are reduced and establish the central metal–metal bond (Figure 10A) as a characteristic feature of the building block.^124^

##### 4.1.2.2 The Keggin-Type

The reassembly of the intact γ-Keggin POT by {M^V^_2_S_2_O_2_} insertion to the lacunary anions γ-[Si^IV^W^VI^_10_O_36_]^8–^^125^ and γ-[P^V^W^VI^_10_O_36_]^7–^^126^ was presented by Cadot et al. accompanied by a detailed structural and electrochemical characterization. They compared the isovalent reduced O-form γ-[Si^IV^W^VI^_10_W^V^_2_O_40_]^6–^ with the S-substituted version γ-[Si^IV^W^VI^_10_O_36_{W^V^_2_S_2_O_2_}]^6–^ (Figure 13C,D for the Mo^V^-analogue) and revealed an important difference in the localization of the two additional electrons by ^183^W NMR analysis.^125^ While in the paramagnetic oxo-form, the additional charge is delocalized over the whole POT framework, recognizable as the typical intense blue color of reduced POMs;^127^ in the colorless diamagnetic thio-analogue, two electrons are localized in the metal–metal bond with μ_2_-S stabilization.^45^ The incorporation of {M^V^_2_S_2_O_2_} also enhances hydrolytic stability in aqueous solution, where the unsubstituted γ-[Si^IV^W^VI^_12_O_40_]^4–^ rearranges to α- and β-isomers at any pH.^127^ The increased charge of the thio-form seems to play a minor role for the charge density here, since the additional electrons are confined to only one side of the molecule.

The immobilized [Si^IV^W^VI^_10_O_36_{W^V^_2_S_2_O_2_}]^6–^ on the glassy carbon electrode was applied to the electroanalysis of iodate anions in aqueous medium with a limit detection of 6.2 μM, which is comparable to those of other previously reported chemically modified electrodes with POMs.^128^

### 4.2 Selenium: The Heaviest Chalcogen for O Substitution in POMs

Se (Pauling EN: 2.55) shows many analogies to S and is suitable for the replacement of O_t_ centers in POMs. Only three structures have been reported so far with this modification. Using the Se transfer agent bis(*n*-octyldimethylsilyl)selenide, Radkov et al.^121^ prepared the Lindqvist anion [W^VI^_5_O_18_{Nb^V^Se}]^3–^ and the Keggin POTs [P^V^W^VI^_11_O_39_{Nb^V^Se}]^4–^ and [P^V^W^VI^_11_O_39_{Ta^V^Se}]^4–^ from the respective mixed-metal oxo-precursors [P^V^M^V^W^VI^_11_O_40_]^4–^ (M = Nb or Ta). The structures were characterized by ^17^O, ^31^P, ^183^W, and ^77^Se NMR and IR spectroscopy. Compared to the analogue S-compounds, the Se-ligands were more susceptible to acidic hydrolysis, especially in the presence of oxygen, and the Ta=Se bond was less stable than the Nb=Se analogue, which probably prevents the isolation of the missing Lindqvist structure in this series, [W^VI^_5_O_18_{TaSe}]^3–^. In accordance with the other chalcogens O and S, the terminal Se^2–^ bonds were less reactive in the Keggin-POTs. The lability of the Se^2–^ groups suggests that Te with even lower electronegativity is not suitable to replace O in a POM framework.

The structural unit {Mo^V^_2_Se_2_O_2_}^129^ has also been reported and may offer routes to novel compounds with bridging Se^2–^ functions when introduced to POMs.

## 5 Oxo-Replacement by Group 17 Elements: Halogens

Halide anions are isoelectronic to oxo ligands, but with only one negative charge, they reduce the overall POM charge. They can replace both terminal and bridging O positions, and due to their weak π-donation, the local electron density at the halo-addenda centers is lower compared to the oxo-sites.

### 5.1 Bridging Fluorido-Ligands: Altering the Reduction Potential of POMs

**Figure 14:**
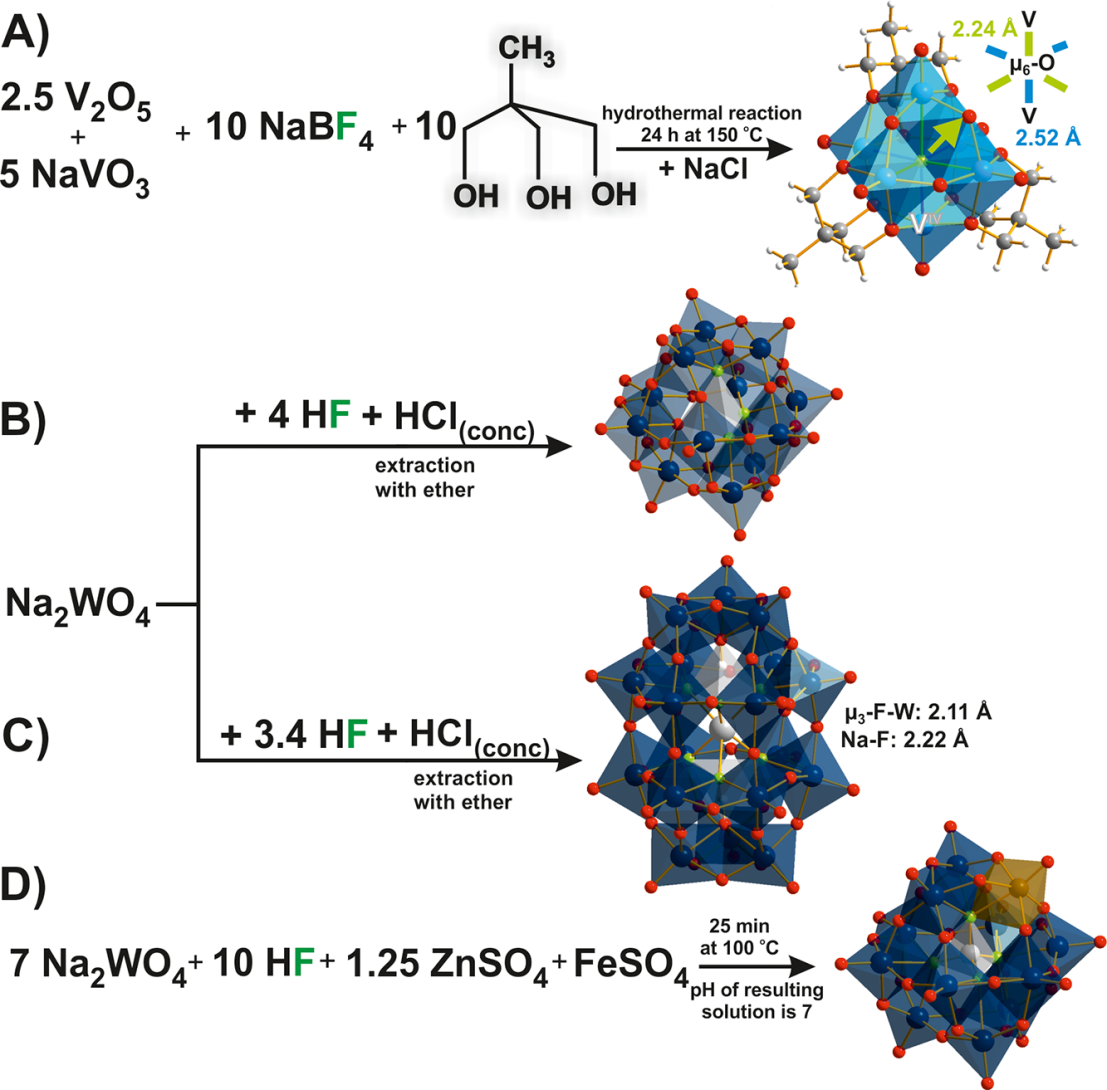
Synthesis scheme of POMs with F^–^ replacing constitutive O sites: (A) Lindqvist hexavanadate [V^IV^_6_O_6_(OH)_3_F((OCH_2_)_3_CCH_3_)_3_]^−^,^130^ (B) metatungstate anion with 3 replaced O [HW^VI^_12_O_37_F_3_]^4–^,^132,133^ and (C) [{NaF_6_}H_2_W^VI^_18_O_56_]^8–^ (in order to emphasize the analogy to the classical α-Wells–Dawson archetype, and the two internal protons were added manually to the structure, forming the center of hydrogen-bonded {OF_3_} tetrahedron).^141^ (D) Keggin PFOM [Zn^II^W^VI^_10_W^V^O_35_F_4_{Fe^III^(OH)}]^5–^ (the reduced W^V^ center is not localized).^43^ The relevant bonds are highlighted in color and their lengths are compared. Color code: dark blue, W; light blue, V^IV^; red, O; yellow-green, F; brown, Fe; light gray, Na or Zn; dark gray, C; white, H.

F (Pauling EN: 3.98) is the only element of the periodic table with a higher Pauling electronegativity than O (Pauling EN: 3.44). As the smallest halide anion, F^–^ (ionic radius 119 pm) features a similar size as oxide (126 pm), but only adopts bridging positions within POM scaffolds to retain its very high charge density. POM archetypes with central μ_3_-O or higher bridging oxygen sites, like the Lindqvist (Figure 1A), the Keggin metatungstate (Figure 14D), and the nonclassical Wells–Dawson anions (Figure 1C), were successfully modified with F^–^ at these positions, where it is enclosed in the center of the POM scaffold and further stabilized by proton or metal coordination. For synthesis, an aqueous HF solution is used to induce the self-assembly condensation process from ortho-metalates, resulting in various fluorination degrees in polyfluorooxometalates (PFOMs).

#### 5.1.1 The Lindqvist-Type

In two reported Lindqvist (Figure 1A) polyfluorooxovanadates, the central μ_6_-O atom was replaced by F^–^ (Figure 14A).^130,131^ In the first case, an intact Lindqvist structure comprising reduced V^IV^ addenda centers and hybridized with three tripodal ligands revealed a particular binding behavior of the central F. Rather than bridging all six addenda atoms in a μ_6_-mode with equal V–F distances, it was clearly shifted toward the single nonfunctionalized POM face (Figure 14A).^130^ Therefore, the authors decided to assign the F^–^ center a μ_3_-coordination mode. Considering the elongation of three bonds due to the alkoxy ligand attachment, μ_6_-F seems to be an appropriate description. The other comparable structure contains a lacunary Lindqvist fragment with a μ_5_-F center connecting all five V^V^ addenda metals, enabling a symmetric connectivity.^131^

#### 5.1.2 The Keggin-Type

The μ_3_-fluorinated Keggin metatungstates [H_2_W^VI^_12_O_39_F]^5–^, [H_2_W^VI^_12_O_38_F_2_]^4–^, and [HW^VI^_12_O_37_F_3_]^4–^ (Figure 14B) were directly obtained in a one-pot approach applying WO_4_^2–^, HF, and HCl^132,133^ and characterized by elemental analysis, ^1^H- and ^19^F-NMR. In the proposed structures, the central metatungstate protons are still mainly bound to the μ_3_-O sites, but the F-ligands participate in the hydrogen bonding interactions. A decrease in the charge of the Keggin anion compared to oxo-analogue [H_2_W^VI^_12_O_40_]^6–^ with increasing F^–^ substitution shifted the hydrolytic stability window of [H_2_W^VI^_12_O_40_]^6–^ (pH ≈ 2–7) to successively lower pH values (e.g., pH ≈ 1–5.8 for [H_2_W^VI^_12_O_39_F]^5–^),^133^ which is consistent with general observations on the charge-dependent hydrolytic stabilities of Keggin POTs.^134^ The theoretical product [W^VI^_12_O_36_F_4_]^4–^ with all four μ_3_-O sites substituted with F has never been reported, probably due to the impossibility of stabilizing the central protons and, as a consequence, decreasing the total negative charge, which leads to pronounced hydrolysis.

The described F-metatungstates [H_2_W^VI^_12_O_38_F_2_]^4–^ and [HW^VI^_12_O_37_F_3_]^4–^ (Figure 14B) hydrolyzed to pure POTs in the pH range 3–4 under partial F^–^ loss to re-establish a sufficiently stable charge. The intermediate reactive lacunary anions were trapped by reassembling with various metals under retention of one,^135,136^ two,^133,137,138^ or even three^133,139^ μ_3_-F sites. Most of these anions were obtained by Wasfi et al. in a one-pot procedure in the presence of the substituting metal without separate preformation of the PFOM fragment. This procedure gave rise to the stable mixed-valence anion [Zn^II^W^VI^_10_W^V^O_35_F_4_{Fe^III^(OH)}]^5–^ (Figure 14C) resulting from the incorporation of Fe^II^ into the fully oxidized PFOM framework and the internal delocalization of an electron from the iron center.^43^ Two important aspects about the PFOM chemistry can be learned. First, the F-replacement of all four central μ_3_-O sites was possible in the direct co-assembly approach, by stabilizing the inner charge density with a Zn^II^ center binding to all F atoms and by substituting one highly charged W^VI^ by Fe^II^. Second, the electron affinity of the PFOM framework was high enough to cause the oxidation of Fe^II^ in an intramolecular redox reaction, yielding a deeply blue and stable crystalline compound. In aqueous solution under air; however, the anion is oxidized to the compound [Zn^II^W^VI^_11_O_35_F_4_{Fe^III^(OH)}]^4–^. To date, all F-substituted Keggin anions (Table S1) were characterized by elemental and mass analysis as well as spectroscopic techniques with plausible structural conclusions, and some of them by powder X-ray diffraction, with no single X-ray structure currently available.

#### 5.1.3 The Wells–Dawson-Type

The second POM archetype suitable for μ_3_-O substitution by F is the nonclassical Wells–Dawson POT archetype, with a hexagonal-prismatically coordinated central group α-[{W^VI^O_6_}(H_2_)_2_W^VI^_18_O_56_]^6–^ (Figure 1C) as first reported by the Cronin group.^30^ Almost 30 years before, Chaveau et al.^140^ prepared a fluorinated Wells–Dawson compound and proposed the six central μ_3_-O atoms of the belt regions to be replaced by F, thereby forming {(μ_3_-F)_3_(μ_3_-O)} tetrahedron around a stabilizing proton. By X-ray crystallographic analysis, Jorris et al.^141^ showed 10 years later that an additional Na^+^ in the center stabilizes the six F-ligands (Figure 14C), establishing the final structure α-[{NaF_6_}H_2_W^VI^_18_O_56_]^8–^^142^ with an obvious analogy to the Wells–Dawson oxo-form α-[{W^VI^O_6_}(H_2_)_2_W^VI^_18_O_56_]^6–^,^30^ which was determined much later. This oxo-structure can be viewed as a central {WO_6_} moiety surrounded by a neutral POT cage with two proton-centered {(μ_3_-O)_4_} tetrahedra as in the metatungstate center (Figure 14B). The central W–O bonds are shortened (1.97 Å), and the μ_3_-O connections are clearly elongated (2.43 Å) with regard to the PFOM structure, pointing to the role of its central Na^+^ for mere charge stabilization. The only available Wells–Dawson PFOM crystal structure is that of the Fe^III^-substituted anion. Unfortunately, the Fe^III^ position is symmetrically disordered over all 18 metal positions; hence, this structure was evaluated as [{NaF_6_}H_2_W^VI^_18_O_56_]^8–^ (cf. Figure 14A). Due to the tight coordination of the reactive F sites in the center, the F_6_-Wells–Dawson POT anion survives lacunarizaton and recomplementation by addenda or transition metals to form a series of substituted compounds.^16,141^ Their increased oxidation potential was applied to assist the epoxidation of alkenes by hydrogen peroxide, and the Ni-derivative exhibited significant catalytic activity under full structural retainment.^16^

In order to incorporate a number of F^–^ ions other than six, a one-pot co-assembly approach was applied, leading to Wells–Dawson POTs with five,^144^ seven, and eight^145^ F sites. Again with no X-ray structural evidence, but reasonable spectroscopic and powder X-ray characterization, it is straightforward to assign the excess F atoms to μ_2_-sites when more than six are present in a structure. Interestingly, when Fe^II^ was used during synthesis, a Wells–Dawson mixed-valence compound [NaH_2_W^V^W^VI^_16_O_54_F_7_{Fe^III^(H_2_O)}]^8–^ was obtained.^145^

### 5.2 Terminal Chlorido-Ligands: Nothing for Electron-Deficient Metals

**Figure 15:**
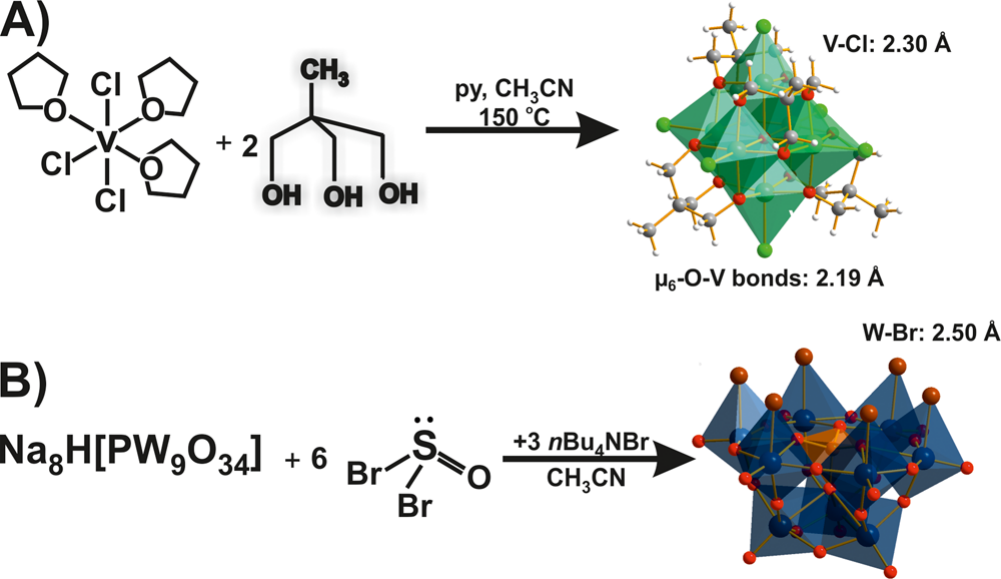
Synthesis scheme of (A) highly reduced and completely hybridized Lindqvist hexavanadate [V^III^_6_Cl_6_O(OCH_2_)_3_CCH_3_)_4_]^2–^^146^ and (B) trivacant Keggin POT A-*β*-[P^V^W^VI^_9_O_28_Br_6_]^3–^.^148^ The relevant bonds are highlighted. Py, pyridine, Bu, butyl. Color code: dark blue, W^VI^; green, V^III^; red, O; green, Cl; brown, Br; dark gray, C; white, H.

Cl (Pauling EN: 3.16) ions are less electronegative and larger than O, which restricts their applicability for the O-substitution in POMs. The usually highly oxidized addenda ions (e.g., V^V^, Mo^VI^) do not get sufficient charge compensation by the weak π-interactions with Cl-ligands. Therefore, this element has never been reported in a bridging O-site and only as a terminal ligand in a highly reduced Lindqvist hexavanadate structures [V^III^_6_Cl_6_O(OCH_2_)_3_CCH_3_)_4_]^2–^^146^ (Figure 15A), [V^III^V^IV^_4_V^V^O_6_Cl(OC_2_H_5_)_12_]^−^^147^ [V^III^V^IV^_3_V^V^_2_O_6_Cl(OC_2_H_5_)_12_]^147^ and [V^III^V^IV^_2_V^V^_3_O_6_Cl(OC_2_H_5_)_12_]^+^^147^ obtained by solvothermal synthesis, where V=O bond cleavage leads to the formation of a unique V^III^–Cl bond. The V^III^ centers do not require extensive electron donation and are stabilized by the complete surface hybridization of POM with four tripodal ligands. The electronic d^2^ configuration of the metal indicates double-bond character, with the Cl^–^ ligand acting as π-donor to the vacant d-orbitals of V^III^ (Figure 2A). This is supported by the slight contraction of all μ_6_-O-bonds by about 0.05 Å due to the weak *trans*-influence of the Cl^–^ ligands.

### 5.3 Terminal Bromido-Ligands: Between Stabilization and Activation of Lacunary Sites

The characteristics described for Cl ligands are even more pronounced for its higher homologue Br (Pauling EN: 2.96). The only POM structure with Br-substitution is the trilacunary Keggin POT A*-β*-[P^V^W^VI^_9_O_28_Br_6_]^3–^ (Figure 15B) obtained by treatment of the O-analogue with the Br transfer agent thionyl bromide in ACN.^148^ The high charge density of the oxo-form A*-β*-[P^V^W^VI^_9_O_34_]^9–^ at its six *cis*-dioxo W centers is reduced by replacing O^2–^ groups with Br^–^. The long bonds to the Br^–^ ions correspond to a simple single-bond interaction. While in non-nucleophilic solvents (e.g., ether), this structure is expected to be stable, and the introduction of weakly bound Br^–^ as a good leaving group certainly activates the POT fragment toward nucleophilic attack. This suggests the use of A*-β*-[P^V^W^VI^_9_O_28_Br_6_]^3–^ as a building block for sandwich POT structures or as a precursor for further modification with organic ligands (e.g., alkoxy groups).

The only remaining element suitable for O-replacement in a POM structure is I (EN: 2.66). It seems feasible to incorporate it into a lacunary POT structure just as shown for Br, but the very diffuse and soft electronic character might prevent its stable attachment.

## 6 Conclusions and Outlook

The possibly first impression that oxo-replaced POMs are *per se* unstable in aqueous solution under air is a strong simplification. The presented survey and comparison of well characterized O-replaced POM structures leads to the following comprehensive conclusions about accessibility and stability of these compounds. (1) POM reactivity toward oxo-replacement depends on: (i) influence of the POM size and the overall charge density: Because of the increasing degree of electronic delocalization with increasing numbers of {MO_6_} fragments in POM frameworks, the O centers are more tightly bound and not reactive enough for replacement. Therefore, direct O-substitution in an intact homometallic POM has only been achieved for the Lindqvist archetype, which exhibits both a compact structure and very low charge density (see section 3.1). (ii) The local charge density on the addenda atom: POMos are more reactive than POTs with less polar metal–oxygen bonds. To replace O sites in POT structures, the local electron density around O-sites can be increased by reducing the effective positive charge of the addenda atom by introducing derivatized reduced addenda ions (see sections 3.1.4, 3.1.6, and 4.1.2), group 5 ions (see sections 4.1.1 and 4.2 and SI), or lacunary sites (see section 5.3 and SI). (iii) Steric accessibility: Most O-replacements have been reported for O_t_ sites which are well-exposed on the POM surface and connected to only one metal ion (see section 1.2). (2) Stability of oxo-replaced POMs depends on: (i) The chemical similarity of the replacing element to O: Effective replacement of O sites requires isolobal ligands with elements of suitable electronegativity in isoelectronic interaction with the metal center (see section 1.2). (ii) The overall charge density: As with oxo analogues, the low charge density is the origin of the inherent instability of the oxo-substituted POMs in aqueous solution at all pH values. In some cases, O-replacing ligands can provide additional stability to the overall POM framework. The charge density effects became very evident in the halide-replaced POM structures. The generally lower charges of PFOMs (see Section 5.1) have shifted their hydrolytic stability window toward the lower pH range. However, the electron affinity is also influenced, which led to more positive reduction potentials with increasing fluoride content (and correspondingly lower charge).

The following synthetic strategies can be considered for further development of this class of POM hybrids: (i) prefunctionalized addenda fragments: The insertion of preformed units into lacunary POMs (see sections 3.1.4, 3.1.6, and 4.1.2) presents a handy route to O-replacement in less reactive larger POT structures such as Wells–Dawson compounds. (ii) Overall charge-density control: For the electron-donating ligands (e.g., imido, hydrazido, see sections 3.1.3–4), isostructural POMs with higher charge density should be more stable in water, and the Keggin scaffold [X^*n*+^M_12_O_40_]^(8–*n*)–^ can be charge adjusted more easily than the Lindqvist structure by proper selection of the central heteroatom. Therefore, it would be highly interesting to assess the hydrolytic stability of O-substituted derivatives. As an example, the imido-tungsten precursor inserted into the lacunary fragment [P^V^W^VI^_11_O_39_]^7–^ yielding [P^V^W^VI^_12_O_39_(N–C_6_H_5_)]^3–^^95^ (*q*/*m* = 0.25, see section 3.1.2) could be inserted into [Al^III^W^VI^_11_O_39_]^9–^ instead, to form [Al^III^W^VI^_12_O_39_(N–C_6_H_5_)]^5–^ (*q*/*m* = 0.42) stable against hydrolysis at neutral pH.^134^

The majority of studies on oxo-replaced POMs have so far focused on bulk synthesis and characterization. However, in the recent literature, there seems to be progress in investigating applications of this class of compounds as second-order nonlinear optical molecular materials^77−79^ and as antitumor agents.^66,90−92,105^ The attachment of known biologically active ligands by oxygen substitution in suitable POMs, as already shown for hydrazides^101^ or amantadines,^92^ can significantly expand the scope of hybrid POMs and become a direction for future research. Since POMs are redox active, their mixture with electroactive organic moieties generates compounds that have an advantage for a wide range of potential applications in the field of photonics and electronics. Only direct oxygen substitution enables the electronic synergies between the conjugated organic bridge and the POM (hexamolybdates) cages in these hybrids, making them an intriguing class of electroactive molecular materials. As the hybrid compounds exhibit improved optical and inhibitory activities compared to the purely inorganic POMs and the corresponding ligands alone, further development in this area is extremely interesting, and this line of research would benefit from more comprehensive studies to elucidate the underlying processes.

## Supporting Information Available

A table with O-substituted POMs structures based on the M–X (M, addenda ion, X, substituting element) structural search in the CCDC database as of November 2021; a structural and synthetic overview of amino-, thio-, and peroxo-substituted POMs (PDF)
