# Supplementary material for: Oxo-Replaced Polyoxometalates: There Is More than Oxygen
Source: ACS Org Inorg Au. 2022 Sep 20;2(6):477–95. doi: 10.1021/acsorginorgau.2c00014 (PMC9732882; doi:10.1021/acsorginorgau.2c00014)
Supplement: Supplementary file 1 — gg2c00014_si_001.pdf [file gg2c00014_si_001.pdf]

Oxo-replaced polyoxometalates: There is more than oxygen

Joscha Breibeck[1], Nadiia I. Gumerova[1] and Annette Rompel[1]*

[1]Universität Wien, Fakultät für Chemie, Institut für Biophysikalische Chemie, Josef-Holaubek-Platz 2, 1090 Wien, Austria, e-mail: annette.rompel@univie.ac.at, fax: +43-1-4277-852502, phone: +43-1-4277-52502, homepage: http://www.bpc.univie.ac.at
* Corresponding author: annette.rompel@univie.ac.at

Supplementary information

Contents

1. Table S1 3

2. Amino, imino and amido ligands – stabilizing agents for labile structures 25

3. An alternative binding-mode for oxygen - the peroxo modification 26

4. Other POM structures based on the {$M_2S_2O_2$} subunit 29

5. References 29

1. Table S1

Table S1: Summary of 283 CCDC crystal structures (as of November 2021) and further 99 relevant structures of oxo-replaced POMs. Abbreviations of methods: COND, conductivity measurement; CSI-MS, cryospray ionization mass spectrometry; CV, cyclic voltammetry; DLS, dynamic light scattering; DSC, differential scanning calorimetry; EA, elemental analysis; EPR, electron paramagnetic resonance; ESI-MS, electrospray ionization mass spectrometry; FAB-MS, fast-atom bombardment mass spectrometry; FS, fluorescence spectroscopy; HRS, hyper-Rayleigh scattering; IR, infrared; MALDI-MS, matrix-assisted laser desorption ionization mass spectrometry; MSU, magnetic susceptibility; NMR, nuclear magnetic resonance; Raman, Raman spectroscopy; SAXS, small-angle X-ray scattering; SCD, solid-state circular dichroism; SLS, static light scattering; Stark, Stark spectroscopy; TEM, transmission electron microscopy; TGA, thermogravimetric analysis; UV-Vis, ultraviolet-visible; UZ, ultrazentrifugation; XPD, X-ray powder diffraction; X-ray, X-ray single crystal structure (not available in CCDC). Abbreviations of solvents and liganda: ACN, acetonitrile; DCM, dichloromethane; EtOH, ethanol; MeOH, methanol; py, pyridine; calix, 4-tert-butylcalix[4]-arene; H_3tri, tris(hydroxymethyl)aminomethane).

Sum formula	POM archetype	Addenda atom with replaced O	Oxo-replacing hetero-atom	Synthesis conditions	Characterization	CCDC number	Reference
**Carbon (8 POMs)**							
$[Mo_6O_{18}((CCH_3)_5)]^{-}$	Lindqvist	Mo^{VI}	C (η^5-cp*)	Organic solvent exposed to air and water	CV, EA, ^{1}H-, ^{13}C-, ^{17}O-, ^{95}Mo-NMR, IR, TGA, UV-Vis	1190850, 114290, 962704, 866671	1,2,3,4
$[W_5MoO_{18}((CCH_3)_5)]^{-}$	Lindqvist	Mo^{VI}		MeOH/H_2O exposed to air	EA, ^{1}H-NMR, IR, TGA	-	4
cis–$[Mo_6O_{17}((CCH_3)_5)_2]^{0}$	Lindqvist	Mo^{VI}		MeOH/H_2O exposed to air	EA, IR	231765	5
$[Mo_5WO_{18}((CCH_3)_5)]^{-}$	Lindqvist	W^{VI}		MeOH/H_2O exposed to air	EA, ^{1}H-NMR, IR, TGA	866670, 866672	4
$[W_6O_{18}((CCH_3)_5)]^{-}$	Lindqvist	W^{VI}		MeOH/H_2O exposed to air	EA, ^{1}H-NMR, IR, TGA	-	4
cis–$[W_6O_{17}((CCH_3)_5)_2]^{0}$	Lindqvist	W^{VI}		dry degassed toluene	EA, ^{1}H-NMR, IR	1195601	6
$[V^{IV}_5V^{III}(OCH_3)_{12}O_6(CNC(CH_3)_3)]^{-}$	Lindqvist	V^{III}	C (isonitrilo)	oxygen-free DCM	CV, EA, ^{1}H-NMR, IR, UV-Vis	-	7
cis–$[V^{IV}_4V^{III}_2(OCH_3)_{12}O_5(CNC(CH_3)_3)_2]^{0}$	Lindqvist	V^{III}		oxygen-free DCM	CV, EA, ^{1}H-NMR, IR, UV-Vis	-	7
**Nitrogen (255 POMs)**							
$[NaV_6O_6\{(OCH_2CH_2)_2NH\}_6]^{+}$	Anderson -like	V^{IV}	N (amino)	solvothermal without solvent	CV, EA, IR, UV-Vis	994749	8
$[MV_6O_6\{(OCH_2CH_2)_3N\}_6]^{n+}$							
M = Li^{I}, n = 1				solvothermal in ACN/EtOH	EA, IR, MSU	236396	9
M = Na^{I}, n = 1				solvothermal in ACN/EtOH	EA, IR, MSU, UV-Vis	236397	9
M = Mg^{II}, n = 2				solvothermal in ACN/EtOH	EA, IR, MSU, XPS	-	9
M = Mn^{II}, n = 2				solvothermal in ACN/EtOH	EA, IR, MSU	196923	9

M = Fe^{II}, n = 2				solvothermal in ACN/EtOH	EA, IR, MSU, UV-Vis	216908	9
M = Co^{II}, n = 2				solvothermal in ACN/EtOH	EA, IR, MSU, XPS	-	9
M = Ni^{II}, n = 2				solvothermal in ACN/EtOH	EA, IR, MSU, XPS	-	9
$[V_{14}O_{18}(tri)_2(Htri)_6(HCOO)_2]$		V^{IV}	N (amino)	solvothermal in H_2O/MeOH	SXRD, IR, UV-Vis	2038973	10
$[(PMo_9O_{31})(NC_5H_5)_3]^{3–}$	Lacunary Keggin	Mo^{VI}	N (imino)	pyridine, ACN	CSI-MS, EA, ESI-MS, ^{1}H-, ^{31}P-NMR, IR	1899175, 1899176	11
$[(PMo_9O_{31})_2(NC_{10}H_8N)_3]^{6–}$				ACN, dioxane	EA, ESI-MS, IR	1899177	11
$[(PMo_9O_{31}(NC_5H_5))_4(C_{20}H_{12}N_4(C_5H_5N)_4)_2]^{12–}$				organic solvent	EA, ESI-MS, ^{1}H-, ^{31}P-NMR, IR, UV-Vis	1899178, 1899179	11
$[(SiW_{10}O_{36}(ONHCCH_3))_2Cu^{II}]^{8–}$	Lacunary Keggin	W^{VI}	N (amido)	acetone	CSI-MS, EA, ^{1}H-NMR, IR, MSU, UV-Vis, TGA	912546	12
$[\gamma–PMo_{10}O_{34}(py)_2]^{3–}$	Keggin	Mo^{VI}	N terminal (amido)	organic solvent	EA, IR, ^{31}P-, ^{183}W-NMR, UV-Vis	2044609	13
$[Mo_3MoW_{l8}O_{31}(OH)_6py_3]^{2–}$	Keggin	Mo^{IV}, Mo^{VI}, W^{VI}	N (amido)	organic solvent, solvothermal	XPS, EA, PXRD, SXRD, IR	973197	14
$[Mo_6W_6O_{28}(OH)_6py_6]^{2–}$	Keggin	Mo^{IV}, W^{VI}	N (amido)	organic solvent, solvothermal	XPS, EA, PXRD, SXRD, IR	973198	14
$[Mo_6W_6O_{29}(OH)_5py_6]^{–}$	Keggin	Mo^{IV}, W^{VI}	N (amido)	organic solvent, solvothermal	XPS, EA, PXRD, SXRD, IR	973199	14
$[Mo_6MoW_5O_{28}(OH)_6py_6]^{2–}$	Keggin	Mo^{IV}, Mo^{VI}, W^{VI}	N (amido)	organic solvent, solvothermal	XPS, EA, PXRD, SXRD, IR	2102185	14
$[(calix)V_6O_6(OCH_3)_8(CH_3OH)]^{–}$	Lindqvist	V^{V}	N (amido)	organic solvent	EA, PXRD, SXRD, IR	2071335	15
$[(calix)V_6O_7(OCH_3)_8]^{–}$	Lindqvist	V^{V}	N (amido)	organic solvent	EA, PXRD, SXRD, IR	2071336	15
$[P_2W_{12}O_{44}(ONHCCH_3)_2M_2(CH_3CO_2)]^{3–}$	Lacunary Dawson	W^{VI}	N (amido)				
M = Mn^{II}				ACN	CSI-MS, CV, EA, IR, MSU, UV-Vis	1883422	16
M = Co^{II}				ACN	CSI-MS, CV, EA, IR, MSU, UV-Vis	1883423	16

M = Ni^{II}				ACN	CSI-MS, CV, EA, IR, MSU, UV-Vis	1883424	16
M = Cu^{II}				ACN	CSI-MS, CV, EA, IR, MSU, UV-Vis	1883425	16
M = Zn^{II}				ACN	CSI-MS, CV, EA, IR, UV-Vis	1883426	16
$[H_8Si_2W_{20}O_{68}(ONHCCH_3)\{Zn^{II}(NCCH_3)\}]^{7-}$	Lacunary anion	W^{VI}	N (amido)	organic solvent	EA, CSI-MS, CV, ^{1}H-, ^{29}Si-NMR, IR, UV-Vis	1568655	17
**$[Mo_6O_{18}(NR)]^{2-}$**	Lindqvist	Mo^{VI}	N (imido)	**90 structures**			
R = CH_3				dry ACN	EA, ESI-MS, ^{1}H-NMR, UV-Vis	-	18
R = C_2H_5				dry ACN	EA, ESI-MS, ^{1}H-NMR, UV-Vis	688713	18
R = C_3H_7				dry ACN	EA, ESI-MS, ^{1}H-NMR, UV-Vis	688714	18
R = C_3H_6Cl				anhydrous ACN	EA, ESI-MS, ^{1}H-NMR, IR, UV-Vis	863193	19
R = C_3H_6I				anhydrous ACN	EA, ESI-MS, ^{1}H-NMR, IR, UV-Vis	863194	19
R = $C_3H_6ONO_2$				anhydrous ACN	EA, ESI-MS, ^{1}H-NMR, IR, UV-Vis	863195	19
R = $CH(CH_3)_2$				dry ACN	EA, ESI-MS, ^{1}H-NMR, UV-Vis	688715	18
R = C_4H_9				organic solvent	CV, EA, ESI-MS, ^{1}H-, ^{14}N-, ^{17}O-, ^{95}Mo-NMR, UV-Vis	141413, 688716	18,20
R = $C(CH_3)_3$				dry ACN	EA, ESI-MS, ^{1}H-NMR, UV-Vis	688717	18
R = C_6H_5 (phenyl)				organic solvent	CV, EA, IR, ^{14}N-, ^{95}Mo-NMR, Raman, UV-Vis	1175492, 1175493	21
R = C_5H_4N (3–pyridyl)				dry ACN	IR, UV-Vis	963551	22
R = C_5H_4N (4–pyridyl)				dry ACN	IR, UV-Vis	963552	22
R = $C_5H_3N(CH_3)$ (3–methyl)				dry ACN	IR, UV-Vis	963550	22

R = $C_5H_4N(CH_3)^+$ (*N*–methyl–2–pyridinium)				dry ACN	ESI-MS, IR, UV-Vis	963553	22
R = $C_5H_4N(C_2H_5)^+$ (*N*–ethyl–2–pyridinium)				dry ACN	ESI-MS, IR, UV-Vis	963554	22
R = C_6H_4F (*o*–fluoro)				anhydrous ACN	EA, ^{1}H-NMR, IR	699341	23
R = C_6H_4F (*p*–fluoro)				anhydrous ACN	EA, ^{1}H-NMR, IR	699344	23
R = C_6H_4Cl (*o*–chloro)				anhydrous ACN	CV, EA, ^{1}H-NMR, IR, UV-Vis	-	24
R = C_6H_4Cl (*m*–chloro)				anhydrous ACN	CV, EA, ^{1}H-NMR, IR, UV-Vis	628819	24
R = C_6H_4Cl (*p*–chloro)				anhydrous ACN under nitrogen	CV, EA, ^{1}H-NMR, IR, UV-Vis	217580	24,25
R = C_6H_4Br (*p*–bromo)				anhydrous ACN under nitrogen	EA, ^{1}H-NMR, IR, UV-Vis	249899	25,26
R = C_6H_4I (*p*–iodo)				anhydrous DMSO	CV, EA, ESI-MS, IR, ^{1}H-, ^{13}C-NMR, Raman, Stark, UV-Vis	1553805	27
R = $C_6H_4(CH_3)$ (*o*–methyl)				anhydrous ACN under nitrogen	EA, ^{1}H-NMR, IR, UV-Vis	217581	28
R = $C_6H_4(CH_3)$ (*p*–methyl)				anhydrous pyridine	CV, EA, ^{1}H-, ^{13}C-NMR, IR, UV-Vis	1188751, 1874154	29
R = C_6H_4CN				pyridine under nitrogen	EA, ^{1}H-NMR, IR, UV-Vis	246155	30
R = $C_6H_4(CO_2H)$				dry DMSO	CV, EA, ^{1}H-, ^{13}C-NMR, IR, UV-Vis	-	31
R = $C_6H_4(CO_2CH_3)$				anhydrous ACN	EA, ^{1}H-NMR, IR, UV-Vis	1181294	32
R = $C_6H_4(CO_2C_2H_5)$				anhydrous ACN	EA, ^{1}H-NMR, IR, UV-Vis	1181293	32
$[(Mo_6O_{18}(NC_6H_4CO_2))_4\{Cu^{II}_2(NCCH_3)_2\}]^{8-}$				anhydrous ACN	DLS, ESI-MS, IR, SLS, TEM, UV-Vis	1001148	33
R = $C_6H_3(CH_3)F$ (*o*–methyl)				anhydrous ACN	EA, ^{1}H-NMR, IR	699342	23
R = $C_6H_3(CH_3)F$ (*m*–methyl)				anhydrous ACN	EA, ^{1}H-NMR, IR	699343	23
R = $C_6H_3(CH_3)Cl$ (*o*–methyl)				dry ACN	-	850377	34

R = $C_6H_3(CH_3)Br$ (*o*–methyl)				anhydrous ACN	CV, EA, IR, ^{1}H-NMR, UV-Vis	632686	35
R = $C_6H_4(CF_3)$ (*o*–fluoromethyl)				anhydrous ACN	EA, ^{1}H-NMR, IR, UV-Vis	1169871	36
R = C_6H_4(*o*–NO_2)				anhydrous ACN	SXRD, EA, ^{1}H-NMR, ESI-MS, UV-Vis	802123	37
R = $C_6H_4(NCH_2N_2)$				anhydrous ACN	CV, EA, ESI-MS, IR, UV-Vis	1577980	38
R = $C_6H_4(CH_2)NCH_2N_2$				anhydrous ACN under nitrogen	CV, ESI-MS, ^{1}H-NMR, IR, UV-Vis	1537996	39
R = $C_6H_3(CH_3)C_6H_3(CH_3)(NH_2)$				anhydrous ACN	ESI-MS, ^{1}H-NMR, IR, UV-Vis	810528	40
R = $C_6H_3(OCH_3)C_6H_3(OCH_3)(NH_2)$				anhydrous ACN	ESI-MS, ^{1}H-NMR, IR, UV-Vis	810527	40
R = $C_6H_4(OCF_3)$ (*p*–fluoromethoxy)				anhydrous ACN	EA, ^{1}H-NMR, IR, UV-Vis	1169872	36
R = $C_6H_4(SCH_3)$ (*m*–methylmercapto)				dry ACN	CV, EA, ^{1}H-NMR, IR, UV-Vis	1031870	41
R = $C_6H_4(SCH_3)$ (*p*–methylmercapto)				dry ACN	CV, EA, ^{1}H-NMR, IR, UV-Vis	1031869	41
R = $C_6H_4(C_2H_5)$ (ethyl)				anhydrous ACN	EA, ^{1}H-NMR, IR, UV-Vis	967186	42
R = $C_6H_4(C_2H_3)$ (ethenyl)				pyridine under nitrogen	EA, ^{1}H-NMR, IR, UV-Vis	151509	43
R = $C_6H_4(C_2H)$ (ethynyl)				anhydrous DMSO	CV, EA, ESI-MS, ^{1}H-, ^{13}C-NMR, IR, Raman, Stark, UV-Vis	1553806	27
R = $C_6H_4(C_2)C_6H_4I$				anhydrous DMSO	CV, EA, ESI-MS, ^{1}H-, ^{13}C-NMR, IR, Raman, Stark, UV-Vis	1553808	27
R = $C_6H_4(C_2)C_6H_4NH_2$				anhydrous ACN	CV, EA, ESI-MS, ^{1}H-, ^{13}C-NMR, HRS, IR, UV-Vis	1428592	44

R = $C_6H_4(C_2)C_6H_4NO_2$				anhydrous DMSO	CV, EA, ESI-MS, ^{1}H-, ^{13}C-NMR, IR, Raman, Stark, UV-Vis	1553809	27
R = $C_6H_4(C_2)C_6H_4N(CH_3)_2$				anhydrous DMSO	CV, EA, ESI-MS, IR, ^{1}H-, ^{13}C-NMR, Raman, Stark, UV-Vis	1553810	27
R = $C_6H_4(C_2)C_6H_4(NC_4H_4)$				anhydrous DMSO	CV, EA, ESI-MS, ^{1}H-, ^{13}C-NMR, HRS, IR, UV-Vis	1428591	44
R = $C_6H_4(C_2)C_6H_4(NC_{10}H_8)$				dry DMSO	CV, EA, ESI-MS, ^{1}H-, ^{13}C-NMR, HRS, IR, Stark, UV-Vis	1837358	45
R = $C_6H_4(C_2)C_6H_4N(C_6H_5)_2$				dry DMSO	CV, EA, ESI-MS, ^{1}H-, ^{13}C-NMR, HRS, IR, Stark, UV-Vis	1837405	45
R = $C_6H_3(CH_3)_2$				dry ACN	EA, ^{1}H-NMR, UV-Vis	164192	46
R = $C_6H_4N(CH_3)_2$				anhydrous organic solvent	CV, EA, ESI-MS, IR, ^{1}H-, ^{13}C-NMR, Raman, Stark, UV-Vis	298785, 1553807	47, 27
R = $C_6H_2(CH_3)_2Br$				anhydrous ACN	CV, EA, ^{1}H-NMR, IR, UV-Vis	632685	35
R = $C_6H_2(CH_3)_2I$				dry ACN	EA, ^{1}H-NMR, UV-Vis	164191	46
R = $C_6H_2(CH_3)_2SCN$				ACN	EA, ESI-MS, ^{1}H-NMR, IR, TGA, UV-Vis	969621	48
R = $C_6H_3(C_2H_5)_2$				dry ACN	EA, ^{1}H-NMR, UV-Vis	-	46
R = $C_6H_4(NC_4H_4)$				anhydrous DMSO	CV, EA, ESI-MS, ^{1}H-, ^{13}C-NMR, HRS, IR, UV-Vis	1428590	44

R = $C_6H_4(C_6H_4N)$				dry DMSO	CV, EA, ^{1}H-, ^{13}C-NMR, IR, UV-Vis	1537328	31
R = $C_6H_2(CH_3)_2C_4HSBr(CH_3)$				dry ACN	CV, ^{1}H-NMR, IR, TGA, UV-Vis	1057032	49
R = $C_6H_2(CH_3)_2(CH)_2CN$				anhydrous THF under nitrogen	EA, ESI-MS, ^{1}H-NMR, IR, UV-Vis	621068	50
R = $C_6H_2(CH_3)_2(CH)_2CO_2C_2H_5$				anhydrous THF under nitrogen	EA, ESI-MS, ^{1}H-NMR, IR, UV-Vis	621066	50
R = $C_6H_2(CH_3)_2CHC(CH_3)CO_2C_2H_5$				anhydrous THF under nitrogen	EA, ESI-MS, ^{1}H-NMR, IR, UV-Vis	621065	50
R = $C_6H_2(CH_3)_2(CH)_2C_6H_5$				anhydrous THF under nitrogen	EA, ESI-MS, ^{1}H-NMR, IR, UV-Vis	621067	50
R = $C_6H_2(CH_3)_2(CH)_2C_6H_4Br$				anhydrous THF under nitrogen	EA, ESI-MS, ^{1}H-NMR, IR, UV-Vis	-	50
R = $C_6H_2(CH_3)_2(C_2H)$				dry ACN	EA, ^{1}H-NMR, UV-Vis	164190	46
R = $C_6H_2(CH_3)_2(C_2)C_6H_4(CH_3)$				organic solvent	EA, ^{1}H-NMR	154486	51
R = $C_6H_2(CH_3)_2(C_2)C_6H_3(C_2H)_2$				dry ACN	CV, EA, FS, ^{1}H-NMR, IR, UV-Vis, TGA	215080	52
R = $C_6H_2(CH_3)_2(C_2)C_6H_3(C(CH_3)_3)_2$				organic solvent	EA, ^{1}H-NMR, UV-Vis	-	51
R = $C_6H_2(CH_3)_2(C_2)(C_5H_4)Fe(C_5H_5)$				anhydrous DMF	CV, ESI-MS, ^{1}H-NMR, FS, IR, UV-Vis	239986	53
R = $C_6H_2(C_2H_5)_2(CH)_2C_6H_5$				anhydrous THF under nitrogen	EA, ESI-MS, ^{1}H-NMR, IR, UV-Vis	-	50
R = $C_6H_2(CH_3)_2(NC_6H_4N)C_6H_2(CH_3)_2(NH_2)$				anhydrous ACN under nitrogen	EA, ESI-MS, IR, UV-Vis	1518113	54
R = $C_6H_2(CH_3)_2(C_2)(C_{15}H_{10}N_3)$				dry ACN	CV, ESI-MS, ^{1}H-, ^{13}C-NMR, UV-Vis	201038	55
R = $C_6H_3(CH(CH_3)_2)_2$				organic solvent	CV, EA, ^{1}H-,^{14}N-, ^{17}O-, ^{95}Mo-NMR, UV-Vis	141414	20, 46
R = $C_6H_2(CH(CH_3)_2)_2Br$				ACN under nitrogen	EA, ESI-MS, ^{1}H-NMR, IR, TGA, UV-Vis	1420505	56

R = C_6H_{11} (cyclohexyl)				organic solvent	CV, EA, ESI-MS, ^{1}H-, ^{14}N-, ^{17}O-, ^{95}Mo-NMR, UV-Vis	1210775, 688718, 688719	18, 20
R = $C_6H_{10}(CO_2H)$ (cyclohexyl)				dry DMSO	CV, EA, ^{1}H-, ^{13}C-NMR, IR, UV-Vis	-	31
R = C_6H_{13} (*n*–hexyl)				dry ACN	EA, ESI-MS, ^{1}H-NMR, UV-Vis	688720	18
R = $C_{10}H_{15}$ (adamantyl)				anhydrous ACN	EA, ESI-MS, IR, UV-Vis	1012853	57
R = $(C_5H_4)Fe(C_5H_5)$				dry pyridine	CV, UV-Vis	123703	58
R = $C_{10}H_7$ (1–naphthyl)				anhydrous ACN	CV, EA, ESI-MS, ^{1}H-NMR, IR, UV-Vis	285280	59
R = $C_{10}H_7$ (2–naphthyl)				anhydrous ACN	COND, EA, ^{1}H-NMR, IR, UV-Vis	843751	60
R = $C_{10}H_6(CH_3)$ (methylnaphthyl)				dry ACN under nitrogen	EA, ESI-MS, ^{1}H-NMR, IR, UV-Vis	617137, 617138, 617139	61
R = $C_{13}H_9$ (fluorenyl)				anhydrous ACN	CV, EA, ESI-MS, IR, UV-Vis	1881932	38
R = $C_{16}H_9$ (pyrenyl)				anhydrous ACN	CV, ^{1}H-, ^{13}C-NMR, IR, MALDI-MS, UV-Vis	879741	62
R = $C_{18}H_{37}$ (*n*–octadecyl)				dry ACN	EA, ESI-MS, ^{1}H-NMR, UV-Vis	-	18
R = bis–2,9–di(4–aminophenyl)–1,10–phenanthroline				dry DMSO	EA, ^{1}H-NMR, UV-Vis, CV, EPR	-	63
R = 2–amino–3–methylbenzoxyl				dry ACN	SXRD, EA, IR, ESI-MS	1061525	64
R = 3–NO_2–C_6H_4				anhydrous ACN	SXRD, EA, IR, ^{1}H-NMR, UV-Vis	671418	65
R = 2–CH_3–4–NO_2–C_6H_3				anhydrous ACN	SXRD, EA, IR, ^{1}H-NMR, UV-Vis	671419	65
R = 2–CH_3–5–NO_2–C_6H_3				anhydrous ACN	SXRD, EA, IR, ^{1}H-NMR, UV-Vis	671420	65
**$[(Mo_6O_{18}(N))_2R]^{4-}$**	Lindqvist	Mo^{VI}	N (imido)	**2 POMs**			

R = $(C_2H_5)C{=}C(C_2H_5)$				dry ACN	SXRD, EA, IR, ^{1}H and ^{13}C NMR, UV-Vis	626589	66
R = $(C_2H_5)C{=}C(C_5H_{11})$				dry ACN	SXRD, EA, IR, ^{1}H and ^{13}C NMR, UV-Vis	626590	
***cis*–[$Mo_6O_{17}(NR_1R_2)_2$]$^{2-}$**	Lindqvist	Mo^{VI}	N (imido)	**24 POMs**			
$R_1 = R_2 = C_4H_9$				anhydrous ACN	EA, ESI-MS, IR, UV-Vis	-	67
$R_1 = R_2 = C(CH_3)_3$				anhydrous ACN	EA, ESI-MS, IR, UV-Vis	869448	67
$R_1 = R_2 = C_6H_5$				organic solvent	CV, EA, IR, ^{14}N-, ^{95}Mo-NMR, Raman, UV-Vis	1175492, 1175493	21
$R_1 = R_2 = C_6H_4(CH_3)$ (*o*–methyl)				anhydrous ACN under nitrogen	EA, ^{1}H-NMR, IR, UV-Vis	232108	28, 68
$R_1 = R_2 = C_6H_4(CF_3)$ (*o*–fluoromethyl)				anhydrous ACN	EA, ^{1}H-NMR, IR, UV-Vis	283877	69
$R_1 = R_2 = C_6H_4(OCH_3)$				dry ACN	EA, ^{1}H-NMR, IR, UV-Vis	255980	70
$R_1 = R_2 = C_6H_4$(*o*–NO_2)				anhydrous ACN	SXRD, EA, ^{1}H-NMR, ESI-MS, UV-Vis	802124	37
$R_1 = R_2 = C_6H_3(CH_3)_2$ (2,6–dimethyl)				dry ACN	CV, EA, FS, ^{1}H-NMR, TGA, UV-Vis	187413, 260161	71, 72
$R_1 = R_2 = C_6H_3(CH_3)(C_2H_5)$				anhydrous ACN under nitrogen	EA, ^{1}H-NMR, IR, UV-Vis	280245	73
$R_1 = R_2 = C_6H_3(CH_3)(CH(CH_3)_2)$				anhydrous ACN under nitrogen	EA, ^{1}H-NMR, IR, UV-Vis	280246	73
$R_1 = R_2 = C_6H_2(CH_3)_3$				dry ACN	EA, ^{1}H-NMR, UV-Vis	-	71
$R_1 = R_2 = C_6H_3(CH(CH_3)_2)_2$				pyridine	CV, EA, ^{1}H-, ^{14}N-, ^{17}O-, ^{95}Mo-NMR, UV-Vis	1174126	20, 74

$R_1 = R_2 = C_6H_2(CH(CH_3)_2)_2Br$				ACN under nitrogen	EA, ESI-MS, ^{1}H-NMR, IR, TGA, UV-Vis	1420506	56
$R_1 = R_2 = C_6H_2(CH(CH_3)_2)_2I$				dry ACN	EA, ^{1}H-NMR, UV-Vis	187412	71
$R_1 = R_2 = C_6H_2(CH(CH_3)_2)_2(CCH)$				anhydrous ACN	CV, DSC, ^{1}H-, ^{13}C-NMR, IR, TGA, UV-Vis	215081	75
$R_1 = R_2 = C_6H_2(CH(CH_3)_2)_2C_6H_2(CH(CH_3)_2)_2NH_2$				ACN under nitrogen	EA, ESI-MS, ^{1}H-NMR, IR, TGA, UV-Vis	1420507	56
$R_1 = R_2 = C_6H_2(CH(CH_3)_2)_2(C_2)(C_5H_4)Fe(C_5H_5)$				anhydrous DMF	CV, ESI-MS, FS, ^{1}H-NMR, IR, UV-Vis	-	53
$R_1 = R_2 = C_6H_2(CH(CH_3)_2)_2(C_2)(C_{15}H_8N_3)$				anhydrous DMF under nitrogen	CV, ESI-MS, ^{1}H-NMR, UV-Vis	272303	76
$R_1 = R_2 = C_6H_{11}$ (cyclohexyl)				anhydrous ACN	CV, EA, ESI-MS, IR, UV-Vis	723236, 869447	67, 77
$R_1 = R_2 = C_{10}H_{15}$ (adamantyl)				anhydrous ACN	EA, ESI-MS, IR, UV-Vis	1012854	57
$R_1 = C_6H_3(CH_3)_2$ (2,6–dimethyl), $R_2 = C(CH_3)_3$				anhydrous ACN	ESI-MS, ^{1}H-NMR, IR, UV-Vis	909670	78
$R_1 = C_6H_3(CH_3)_2$ (2,6–dimethyl), $R_2 = C_6H_2(CH(CH_3)_2)_2$ (2,6–diisopropyl)				anhydrous ACN	ESI-MS, ^{1}H-NMR, IR, UV-Vis	909669	78
$R_1 = C_6H_{11}$ (cyclohexyl), $R_2 = C(CH_3)_3$				anhydrous ACN	ESI-MS	-	78
$R_1 = C_6H_3(CH_3)_2$ (2,6–dimethyl), $R_2 = C_{10}H_7$ (naphthyl)				anhydrous ACN	ESI-MS	-	78
***trans*–$[Mo_6O_{17}(NR)_2]^{2–}$**	Lindqvist	Mo^{VI}	N (imido)	**5 POMs**			
$R = C_6H_4(NH_2)$				benzonitrile	CV, ^{1}H-NMR	1303793	79
$R = C_6H_3(CH_3)_2$ (2,6–dimethyl)				anhydrous ACN	EA, IR, ^{1}H-NMR, UV-Vis	294610	80
$R = C_6H_3(CH_3)_2$ (2,4–dimethyl)				anhydrous ACN	IR, UV-Vis	264240	81
$R = C_{10}H_7$ (1–naphthyl)				anhydrous ACN	COND, EA, ^{1}H-NMR, IR, UV-Vis	843752	60
$R = C_{10}H_{15}$ (adamantyl)				anhydrous ACN	EA, ESI-MS, IR, UV-Vis	1012855	57
***cis*–$[Mo_6O_{16}(NR)_2(\mu_2–NR)]^{2–}$**	Lindqvist	Mo^{VI}	N (imido)	**1 POM**			

R = $C_6H_3(CH_3)_2$				anhydrous ACN under N_2	EA, ESI-MS, ^{1}H-NMR, IR, UV-Vis	671814	82
***fac*–[$Mo_6O_{16}(NR)_3$]$^{2-}$**	Lindqvist	Mo^{VI}	N (imido)	**2 POMs**			
R = $C_6H_3(CH(CH_3)_2)_2$				pyridine	CV, EA, ^{1}H-, ^{14}N-, ^{17}O-, ^{95}Mo-NMR, UV-Vis	141415	20
R = $C_{10}H_{15}$ (adamantyl)				anhydrous ACN	EA, ESI-MS, IR, UV-Vis	1012856	57
***mer*–[$Mo_6O_{16}(NR)_3$]$^{2-}$**	Lindqvist	Mo^{VI}	N (imido)	**No structures!**			
***fac*–[$Mo_6O_{15}(NR)_4$]$^{2-}$**	Lindqvist	Mo^{VI}	N (imido)	**1 POM**			
R = $C_6H_3(CH(CH_3)_2)_2$				pyridine	CV, EA, ^{1}H-, ^{14}N-, ^{17}O-, ^{95}Mo-NMR, UV-Vis	1174127	20, 74
***mer*–[$Mo_6O_{15}(NR)_4$]$^{2-}$**	Lindqvist	Mo^{VI}	N (imido)	**No structures!**			
**[$Mo_6O_{14}(NR)_5$]$^{2-}$**	Lindqvist	Mo^{VI}	N (imido)	**2 POMs**			
R = $C_6H_3(CH_3)_2$				anhydrous ACN under N_2	EA, ESI-MS, ^{1}H-NMR, IR, UV-Vis	671815	82
R = $C_6H_3(CH(CH_3)_2)_2$				pyridine	CV, EA, ^{1}H-, ^{14}N-, ^{17}O-, ^{95}Mo-NMR, UV-Vis	141416	20
***fac*–[$Mo_6O_{14}(NR)_4(\mu_2$–NR)]$^{2-}$**	Lindqvist	Mo^{VI}	N (imido)	**1 POM**			
R = $C_6H_3(CH_3)_2$				anhydrous ACN under N_2	EA, ESI-MS, ^{1}H-NMR, IR, UV-Vis	671815	82
**H[$Mo_6O_{13}(NR)_6$]$^{-}$**	Lindqvist	Mo^{VI}	N (imido)	**1 POM**			
R = $C_6H_3(CH(CH_3)_2)_2$				pyridine	EA, ^{1}H-NMR, IR, UV-Vis	1217511	83
***fac*–[$Mo_6O_{13}(NR)_3(\mu_2$–NR$)_3$]$^{2-}$**	Lindqvist	Mo^{VI}	N (imido)	**1 POM**			
R = C_6H_5				No information available		1033546	84
[$W_5MoO_{18}(NC_5H_3(CH_3)_2)$]$^{2-}$	Lindqvist	Mo^{VI}	N (imido)	dry ACN	EA, ^{1}H-NMR, UV-Vis	166680	85
[$W_5MoO_{18}(NC_5H_2(CH_3)_2I)$]$^{2-}$	Lindqvist	Mo^{VI}	N (imido)	dry ACN	EA, ^{1}H-NMR, UV-Vis	166681	85
**[$(Mo_6O_{18})_2(NRN)$]$^{4-}$**	Lindqvist	Mo^{VI}	N (imido)				
R = C_6H_4 (phenyl)				dry pyridine	CV, ^{1}H-NMR, UV-Vis	-	86
R = $C_6H_3(CH_3)$ (tolyl)				dry pyridine	CV, ^{1}H-NMR, UV-Vis	-	86

R = C_6H_{10} (cyclohexyl)				dry pyridine	CV, ^{1}H-NMR, UV-Vis	X-ray	86
R = $C_6H_4(OC_2H_4O)C_6H_4$				anhydrous ACN	EA, ESI-MS, ^{1}H-NMR, IR, UV-Vis	838826	87
R = $C_6H_4(OC_4H_8O)C_6H_4$				anhydrous ACN	EA, ESI-MS, ^{1}H-NMR, IR, UV-Vis	838827	87
R = $C_6H_4(OC_6H_{12}O)C_6H_4$				anhydrous ACN	EA, ESI-MS, ^{1}H-NMR, IR, UV-Vis	838828	87
Lindqvist bridged by Anderson hybrid	Lindqvist	Mo^{VI}	N (imido)				
$[H_2\{Mn^{III}Mo_6O_{18}((OCH_2)_3CN)_2\}(Mo_6O_{18})]^{5-}$				microwave synthesis without dehydrating agent	EA, ESI-MS, ^{1}H-NMR, IR, SAXS, UV-Vis	1418707	88
**$[\{MMo_6O_{18}((OCH_2)_3CN)\}_2\{Mo_6O_{17}(XR)\}_2]^{7-}$**	Lindqvist	Mo^{VI}	N (imido)				
M = Mn^{III}, XR = O				anhydrous ACN	EA, ESI-MS, ^{1}H-NMR, IR, SCD, UV-Vis	785765	89
M = Fe^{III}, XR = O				anhydrous ACN	EA, ESI-MS, ^{1}H-NMR, IR, SCD, UV-Vis	785764	89
M = Mn^{III}, XR = $NC_6H_4(OCH_3)$ (*o*–methoxy)				anhydrous ACN	EA, ESI-MS, ^{1}H-NMR, IR, UV-Vis	-	90
M = Mn^{III}, XR = $NC_6H_3(CH_3)_2$ (2,6–dimethyl)	Lindqvist	Mo^{VI}	N (imido)	anhydrous ACN	EA, ESI-MS, ^{1}H-NMR, IR, UV-Vis	890245	90
M = Mn^{III}, XR = $NC_6H_2(CH_3)_2I$ (2,6–dimethyl–4–iodo)				anhydrous ACN	EA, ESI-MS, ^{1}H-NMR, IR, UV-Vis	-	90
M = Mn^{III}, R = $NC_6H_2(CH_3)_3$ (2,4,6–trimethyl)				anhydrous ACN	EA, ESI-MS, ^{1}H-NMR, IR, UV-Vis	890246	90
M = Mn^{III}, R = $NC_{10}H_7$ (1–naphthyl)				anhydrous ACN	EA, ESI-MS, ^{1}H-NMR, IR, UV-Vis	-	90
M = Mn^{III}, R = $NC_{10}H_6(CH_3)$ (2–methyl–1–naphthyl)				anhydrous ACN	EA, ESI-MS, ^{1}H-NMR, IR, UV-Vis	-	90
Lindqvist bridged by Lindqvist hybrid	Lindqvist	Mo^{VI}	N (imido)				
$[\{V_6O_{13}((OCH_2)_3CN)\}_2(Mo_6O_{18})]^{6-}$				anhydrous ACN, pyridine	CV, EA, ESI-MS, ^{1}H-NMR, IR, UV-Vis	1013553	91
$[W_6O_{18}(NC_6H_3(CH(CH_3)_2)_2)]^{2-}$	Lindqvist	W^{VI}	N (imido)	organic solvent	^{1}H-NMR, UV-Vis	1303059	92

$[PW_{12}O_{39}(NC_6H_5)]^{3-}$	Keggin	W^{VI}	N (imido)	anhydrous ACN under N_2	^{1}H-, ^{31}P-, ^{183}W-NMR, IR	-	93
**$[Mo_6O_{18}(NNR_1R_2)]^{2-}$**	Lindqvist	Mo^{VI}	N (hydrazido)				
$R_1 = CH_3$, $R_2 = C_6H_5$ (phenyl)				organic solvent	CV, EPR, IR	1166740	94
**$[Mo_6O_{18}(NNR)]^{3-}$**	Lindqvist	Mo^{II}	N (bent diazenido)				
R = C_6H_5 (phenyl)				organic solvent	CV, IR, ^{95}Mo-NMR, UV-Vis	1164945	95, 96, 97
R = C_6F_5 (perfluorophenyl)				organic solvent	CV, EPR, ^{95}Mo-NMR	1167093	95, 97
R = $C_6H_3(NO_2)_2$ (2,4–dinitro)				no information available		1298686, 1298687	98
R = C_6H_4–*o*–NO_2				methanol	EA, IR, UV-Vis, ^{95}Mo-NMR, SXRD	204770	97
R = C_6H_4–*p*–CO_2H				methanol	EA, IR, UV-Vis, ^{95}Mo-NMR, SXRD	204771	97
R = C_6H_4–*p*–CH_3				methanol	EA, IR, UV-Vis, ^{95}Mo-NMR	-	97
R = C_6H_4–*p*–F				methanol	EA, IR, UV-Vis, ^{95}Mo-NMR	-	97
R = C_6H_4–*p*–CN				methanol	EA, IR, UV-Vis, ^{95}Mo-NMR	-	97
R = C_6H_4–*p*–OCH_3				methanol	EA, IR, UV-Vis, ^{95}Mo-NMR	-	97
R = C_6H_4–*o*–Cl				methanol	EA, IR, UV-Vis, ^{95}Mo-NMR	-	97
R = C_6H_4–*p*–NO_2				methanol	EA, IR, UV-Vis, ^{95}Mo-NMR	-	97
R = C_6H_3–o,*p*–$(NO_2)_2$				methanol	EA, IR, UV-Vis, ^{95}Mo-NMR	-	97
$[Mo_5O_{13}(OCH_3)_4(NNC_6H_4NO_2)\{Na(OCH_3)\}]^{2-}$ (*o*–nitro)	Lindqvist (lacunary)	Mo^{II}	N (bent diazenido)	methanol	EA, IR, UV-Vis	-	99
$[Mo_5O_{13}(OCH_3)_4(NNC_6H_4NO_2)\{Na(OCH_3)\}]^{2-}$ (*m*–nitro)					EA, IR, UV-Vis	-	99
$[Mo_5O_{13}(OCH_3)_4(NNC_6H_4NO_2)\{Na(OCH_3)\}]^{2-}$ (*p*–nitro)	Lindqvist (lacunary)	Mo^{II}	N (bent diazenido)	methanol	EA, IR, ^{95}Mo-NMR, UV-Vis	771250	99
$[Mo_5O_{13}(OCH_3)_4(NNC_6H_3(NO_2)_2)\{Na(OCH_3)\}]^{2-}$ (o,*p*–dinitro)					EA, IR, UV-Vis	-	99

$[Mo_5O_{13}(OCH_3)_4(NNC_6F_5)\{Na(OCH_3)\}]^{2-}$					EA, IR, UV-Vis	-	99
$[(Mo_5O_{13}(OCH_3)_4(NNC_6F_5))_2Ba]^{4-}$	Lindqvist (lacunary)	Mo^{II}	N (bent diazenido)	methanol	EA, IR, UV-Vis	-	99
$[(Mo_5O_{13}(OCH_3)_4(NNC_6F_5))_2Bi^{III}]^{3-}$					EA, IR, UV-Vis	-	99
$\mathbf{[(Mo_5O_{13}(OCH_3)_4(NNC_6H_4NO_2))_2M]^{n-}}$							
M = Er^{III}, n = 3	Lindqvist (lacunary)	Mo^{II}	N (bent diazenido)	methanol	EA, MSU	1482841	100
M = Yb^{III}, n = 3					MSU	1482842	100
M = Tb^{III}, n = 3					MSU	1482838	100
M = Dy^{III}, n = 3					MSU	1482839	100
M = Ho^{III}, n = 3					MSU	1482840	100
M = Bi^{III}, n = 3					EA, IR, UV-Vis	771251	99
M = Ba^{II}, n = 4					EA, IR, UV-Vis	-	99
$\mathbf{[Mo_6O_{18}(NNCOAr)]^{3-}}$ Ar = Ph	Lindqvist	Mo^{II}	N (bent diazenido)	dry ACN	SXRD, IR, ^1H NMR, UV-Vis, CV	966752	101
Ar = Ph–*o*-Cl				dry ACN	SXRD, IR, ^1H NMR, UV-Vis, CV	966753	101
Ar = Ph–*m*-Cl				dry ACN	SXRD, IR, ^1H NMR, UV-Vis, CV	966754	101
Ar = Ph–*p*-Cl				dry ACN	SXRD, IR, ^1H NMR, UV-Vis, CV	966755	101
Ar = Ph–*o,p*-Cl_2				dry ACN	SXRD, IR, ^1H NMR, UV-Vis, CV	966756	101
Ar = Ph–*p*-Br				dry ACN	SXRD, IR, ^1H NMR, UV-Vis, CV	1422571	101
Ar = Ph–*m*-NO_2				dry ACN	SXRD, IR, ^1H NMR, UV-Vis, CV	966757	101
Ar = Ph–*p*-NO_2				dry ACN	SXRD, IR, ^1H NMR, UV-Vis, CV	966758	101
Ar = Ph–*p*-OMe				dry ACN	SXRD, IR, ^1H NMR, UV-Vis, CV	966759	101
$[PW_{11}O_{39}\{Mo(NNC_6H_4NO_2)\}]^{4-}$ (*o*–, *m*– or *p*–nitro)	Keggin	Mo^{II}	N (bent diazenido)	organic solvent	EA, IR, ^{31}P-, ^{183}W-NMR, UV-Vis	-	99
$[Mo_6O_{18}(NNC(CH_3)(C_6H_4OCH_3))]^{2-}$	Lindqvist	Mo^{VI}	N (diazo-alkane)	pyridine	CV, IR, UV-Vis	102874	102
$[Mo_6O_{18}(NNC_8H_7SN)]^{2-}$ (cyclic thiosemicarbazide)	Lindqvist	Mo^{VI}	N (thiosemi-carbazido)	dry ACN	CV, EA, ESI-MS, ^1H-, ^{13}C-NMR, IR, UV-Vis	766126	103

$[Mo_6O_{18}(NO)]^{3-}$	Lindqvist	Mo^{II}	N (nitrosyl)	organic solvent	CV, EA, IR, ^{14}N -, ^{17}O-, ^{95}Mo-NMR	1183659	104, 105
$[Mo_6O_{17}(OCH_3)(NO)]^{2-}$				organic solvent	CV, EA, ^{1}H-, ^{13}C-, ^{14}N-, ^{17}O-, ^{95}Mo-NMR	1291158	104
$[W_5O_{18}\{Mo(NO)\}]^{3-}$				distilled acetonitrile	CV, EA, IR, ^{14}N-, ^{17}O-, ^{183}W-NMR, UV-Vis, X-ray cell parameters	-	104
$[Mo_5O_{18}\{W(NO)\}]^{3-}$		W^{II}			CV, EA, IR, ^{14}N-, ^{17}O-, ^{95}Mo-, ^{183}W-NMR, UV-Vis, X-ray cell para-meters	-	104
$[W_6O_{18}(NO)]^{3-}$					CV, EA, IR, ^{14}N-, ^{17}O-, ^{183}W-NMR, UV-Vis, X-ray cell parameters	-	104
$[\{Na(OCH_3)\}Mo_5O_{13}(OCH_3)_4(NO)]^{2-}$	Lindqvist (lacunary)	Mo^{II}	N (nitroso)	organic solvent	CV, ^{95}Mo-NMR	1183657, 1183658	105, 106
$[\{Na(OC_2H_5)\}Mo_5O_{13}(OCH_3)_4(NO)]^{2-}$				methanol	EA, IR	-	106
$[\{Li(OCH_3)\}Mo_5O_{13}(OCH_3)_4(NO)]^{2-}$					EA, IR	-	106
$[\{Na(OCHN(CH_3)_2)\}Mo_5O_{13}(OCH_3)_4(NO)]^{2-}$					EA, IR	1291387	106
$[Mo_5O_{13}(OCH_3)_4(NO)\{Mn^{II}Br\}]^{2-}$					EA, IR, UV-Vis	193006	107
$[Mo_5O_{13}(OCH_3)_4(NO)\{Mn^{II}Cl\}]^{2-}$	Lindqvist (lacunary)	Mo^{II}	N (nitroso)	methanol	EA, IR, UV-Vis	-	107
$[(Mo_5O_{13}(OCH_3)_4(NO))_2Re^{II}(H_2O)(CO)_3]^{2-}$					EA, ^{1}H-, ^{13}C-NMR, IR	193003	107
**$[(Mo_5O_{13}(OCH_3)_4(NO))_2M]^{n-}$**	Lindqvist (lacunary)	Mo^{II}	N (nitroso)				
M = La^{III}, n = 3				anhydrous methanol	EA, ESI-MS, IR, MSU, UV-Vis	1511988	108
M = Ce^{III}, n = 3				anhydrous methanol	EA, ESI-MS, IR, MSU, UV-Vis	1511989, 115283	108, 109
M = Nd^{III}, n = 3				anhydrous methanol	EA, ESI-MS, IR, MSU, UV-Vis	1511990	108
M = Sm^{III}, n = 3				anhydrous methanol	EA, ESI-MS, IR, MSU, UV-Vis	1511991	108

M = Gd^{III}, n = 3				anhydrous methanol	EA, ESI-MS, IR, MSU, UV-Vis	1511992	108
M = Eu^{III}, n = 3				anhydrous methanol	EA, ESI-MS, IR, MSU, UV-Vis	1512443	108, 109
M = Dy^{III}, n = 3				anhydrous methanol	EA, ESI-MS, IR, MSU, UV-Vis	1511994	108
M = Tb^{III}, n = 3				anhydrous methanol	EA, ESI-MS, IR, MSU, UV-Vis	1511993	108
M = Ho^{III}, n = 3				anhydrous methanol	EA, ESI-MS, IR, MSU, UV-Vis	1511995	108
M = Er^{III}, n = 3				anhydrous methanol	EA, ESI-MS, IR, MSU, UV-Vis	1511996	108
M = Bi^{III}, n = 3				methanol	EA, ^{1}H-NMR, IR, UV-Vis	115282	109
M = Ca^{II}, n = 4				methanol	EA	-	109
M = Sr^{II}, n = 4				methanol	EA, IR	-	109
M = Ba^{II}, n = 4				methanol	EA, IR, UV-Vis	115284	109
M = Ag^{I}_2, n = 4				methanol	EA, IR, UV-Vis	1189309	110
M = $Mn^{II}(H_2O)_2$, n = 4				methanol	EA, IR, UV-Vis	193005	107
M = $\{Na^{I}(H_2O)(OCH_3)\}_2$, n = 4				methanol	EA, IR	1291385	106
M = $\{Na^{I}(H_2O)\}_2$, n = 4				methanol	EA, IR	1291386	106
$[(Mo_5O_{13}(OCH_3)_4(NO)\{Mn^{II}(CO)_3\})_2Na]^{3-}$	Lindqvist (lacunary)	Mo^{II}	N (nitroso)	methanol	EA, IR, UV-Vis	193004	107
$[\{Na(OCH_3)\}W_4MoO_{13}(OCH_3)_4(NO)]^{2-}$					EA, ^{1}H-, ^{183}W-NMR, IR, UV-Vis, X-ray cell para-meters	-	109
$[Bi^{III}(W_4MoO_{13}(OCH_3)_4(NO))_2]^{3-}$	Lindqvist (lacunary)	Mo^{II}	N (nitroso)	methanol	EA, IR	115285	109
$[\{Ni(MeOH)_2\}_2\{Mo(NO)\}_2(\mu_3\text{-}OH)_2(\mu\text{-}OMe)_4\{Mo_5O_{13}(OMe)_4(NO)\}_2]^{2-}$					EA, IR, MSU, UV-Vis	138681	111
$[PMo_{12}O_{39}(NO)]^{4-}$	Keggin	Mo^{II}	N (nitroso)	organic solvent	EA, IR, ^{31}P-, ^{183}W-NMR, X-ray cell parameters	-	112
$[PW_{11}O_{39}\{Mo(NO)\}]^{4-}$					EA, IR, ^{31}P-NMR	-	112
$[V^{V}V^{IV}_4V^{III}(OCH_3)_{12}O_6(NCCH_3)]^0$	Lindqvist	V^{III}	N (nitrilo)	oxygen-free ACN	CV, EA, ^{1}H-NMR, IR, UV-Vis	1896509	7
cis–$[V^{IV}_4V^{III}_2(OCH_3)_{12}O_5(NCCH_3)_2]^0$					CV, EA, ^{1}H-NMR, IR, UV-Vis	1883474	7

**Oxygen (peroxide) (14 POMs)**							
$H_3[Nb_6O_{13}(O_2)_6]^{5-}$	Lindqvist	Nb^{V}	O (η^2-peroxo)	H_2O	ESI-MS, ^{17}O-NMR	710790	113
$[Nb_{10}O_{27}(O_2)]^{6-}$	Deca-niobate	Nb^{V}	O (η^2-peroxo)	H_2O	ESI-MS, ^{17}O-NMR	-	113
$[Nb_{10}O_{26}(O_2)_2]^{6-}$	Deca-niobate	Nb^{V}	O (η^2-peroxo)	H_2O	ESI-MS, ^{17}O-NMR	-	113
A,β–$[PW_9O_{37}(NbO_2)_3]^{6-}$	Keggin	Nb^{V}	O (η^2-peroxo)	H_2O	EA, FAB-MS, IR, ^{31}P-, ^{183}W-NMR	1645702	114
α–$[SiW_{11}O_{39}(NbO_2)]^{5-}$	Keggin	Nb^{V}	O (η^2-peroxo)	H_2O	EA, IR, ^{29}Si-, ^{183}W-NMR	-	115
A,β–$[SiW_9O_{37}(NbO_2)_3]^{7-}$	Keggin	Nb^{V}	O (η^2-peroxo)	H_2O	EA, FAB-MS, IR, TGA, ^{183}W-NMR	-	116
α₁–$[P_2W_{17}O_{61}(NbO_2)]^{7-}$	Wells-Dawson	Nb^{V}	O (η^2-peroxo)	H_2O	EA, IR, ^{31}P-, ^{183}W-NMR	-	117
α₂–$[P_2W_{17}O_{61}(NbO_2)]^{7-}$	Wells-Dawson	Nb^{V}	O (η^2-peroxo)	H_2O	EA, IR, ^{31}P-, ^{183}W-NMR	-	117
$[P_2W_{12}O_{56}(NbO_2)_6]^{12-}$	Wells-Dawson	Nb^{V}	O (η^2-peroxo)	H_2O	EA, ^{31}P-, ^{183}W-NMR	X-ray	118
$[Mo_6O_{18}(O_2)]^{2-}$	Lindqvist	Mo^{VI}	O (η^2-peroxo)	H_2O	ESI-MS	-	119
$[Mo_6O_{17}(O_2)_2]^{2-}$					ESI-MS	-	119
$[Mo_6O_{16}(O_2)_3]^{2-}$					ESI-MS	-	119
$[Mo_6O_{15}(O_2)_4]^{2-}$					ESI-MS	-	119
β₃–$[Co^{II}W_{11}O_{35}(O_2)_4]^{10-}$	Keggin (lacunary)	W^{VI}	O (η^2-peroxo)	H_2O	EA, EPR, IR, MSU, UV-Vis	1647800	120
**Sulfur (34 POMs)**							
$[W_5O_{18}NbS]^{3-}$	Lindqvist	Nb^{V}	S (terminal sulfido)	dry ACN	IR, ^{17}O-NMR	-	121
$[W_5O_{18}TaS]^{3-}$		Ta^{V}		dry ACN	IR, ^{17}O-NMR	-	121
$[PW_{11}O_{39}NbS]^{4-}$	Keggin	Nb^{V}	S (terminal sulfido)	degassed ACN	EA, IR, ^{31}P-NMR, Raman, X-ray cell parameters	-	122
Structures with incorporated $\{M^{V}_2S_2O_2\}$–unit (M = Mo, W)							
γ–$[PW_{10}Mo_2S_2O_{38}]^{5-}$	Keggin	Mo^{V}	S (μ_2-sulfido)	H_2O or organic solvent	EA, IR, ^{31}P-, ^{95}Mo-, ^{183}W-NMR	-	123
γ–$[SiW_{10}Mo_2S_2O_{38}]^{6-}$	Keggin	Mo^{V}	S (μ_2-sulfido)	H_2O or organic solvent	EA, IR, UV-Vis, ^{183}W-NMR	1266973	124
γ–$[PW_{12}S_2O_{38}]^{5-}$		W^{V}		H_2O or organic solvent	EA, IR, ^{31}P-, ^{183}W-NMR	-	123

γ–$[SiW_{12}S_2O_{38}]^{6-}$		W^V		H_2O	EA, IR, UV-Vis, ^{183}W-NMR	1266972	124
$[(PW_9O_{34})_2(Mo_2S_2O_2)_3]^{12-}$	Keggin-Sandwich	Mo^V	S (μ_2-sulfido)	H_2O	EA, IR, ^{31}P-, ^{183}W-NMR	1647764, 1593479	125
$[(PW_9O_{34})_2(W_2S_2O_2)_3]^{12-}$		W^V		H_2O and organic solvent	EA, IR, ^{31}P-, ^{183}W-NMR	-	125
$[(PW_{11}O_{39})_2(Mo_2S_2O(OH)_2)_2]^{10-}$		Mo^V		H_2O	EA, ^{31}P-, ^{183}W-NMR	X-ray	126
$[(BW_{11}O_{39})_2(W_2O_5(H_2O)_2)(Mo_2S_2O_2)_3(OH)_4(H_2O)_2]^{10-}$		Mo^V		H_2O	EA, ESI-MS, UV-Vis, ^{183}W-NMR	890221	127
Cyclic structures based on the $\{M^V{}_2S_2O_2\}$–unit (M = Mo, W)							
$[(HPO_4)_4Mo_6O_6S_6(OH)_3]^{5-}$	Cyclic anion	Mo^V	S (μ_2-sulfido)	H_2O	EA, IR, ^{31}P-NMR, TGA	1726470	128
$[(HPO_4)_4Mo_6O_9S_3(OH)_3]^{5-}$				H_2O	EA, IR, ^{31}P-NMR	135261	129
$[(HAsO_4)_4Mo_6O_6S_6(OH)_3]^{5-}$				H_2O	EA, IR, TGA	1726469	128
$[Mo_8O_8S_8(H_2O)_8(C_2O_4)]^{2-}$				H_2O	EA, ESI-MS, ^{1}H-NMR	155360, 1208239	130
$[Mo_8O_8S_8(OH)_8\{MoO_4(H_2O)_2\}]^{2-}$	Cyclic anion	Mo^V	S (μ_2-sulfido)	degassed H_2O under N_2	EA, IR	1151933	131
$[Mo_8O_8S_8(OH)_8\{WO_4(OH)(H_2O)\}]^{3-}$				H_2O	COND, EA, IR, neutron diffraction, TGA	1727436, 1727437	132
$[Mo_{10}O_{10}S_{10}(OH)_{12}(H_2O)_3Cl]^{3-}$				H_2O	EA, IR	-	133
$[Mo_{10}O_{10}S_{10}(OH)_{10}(H_2O)_5Cl_2]^{2-}$				DMF	EA, IR	1288898	133
$[Mo_{10}O_{10}S_{10}(OH)_{10}(H_2O)_5I_2]^{2-}$				DMF	EA, IR	141506	134
$[Mo_{10}S_{10}O_{10}(OH)_{10}(H_6C_5O_4)]^{2-}$				H_2O	EA, ESI-MS, ^{1}H-NMR, IR	158056, 158057	130
$[(H_2PO_4)Mo_{10}O_{10}S_{10}(OH)_{11}(H_2O)_2]^{2-}$				H_2O	EA, IR, ^{31}P-NMR, TGA	116673	135
$[Mo_{10}O_{10}S_{10}(OH)_{10}(C_5O_5)]^{2-}$	Cyclic anion	Mo^V or W^V	S (μ_2-sulfido)	H_2O	EA, IR	936796	136
$[Mo_{12}O_{12}S_{12}(OH)_{12}(H_2O)_6]$				H_2O	IR	1725468	137
$[Mo_{12}S_{12}O_{12}(OH)_{12}(H_{10}C_7O_4)]^{2-}$				H_2O	EA, ESI-MS, ^{1}H-NMR, IR	158058	130
$[(H_2PO_4)_2Mo_{12}O_{12}S_{12}(OH)_{12}(H_2O)_2]^{4-}$				H_2O	EA, IR, ^{31}P-NMR, TGA	1726295	135
$[W_{16}O_{16}S_{16}(OH)_{16}(H_2O)_4(C_5H_6O_4)_2]^{4-}$				H_2O	EA, ^{1}H-NMR	146999, 147000	138

$[(Mo_6S_6O_{10})_3(C_4O_4)_9]^{12-}$				H_2O	EA, IR	975304	136
$[(Mo_2O_2S_2)_{12}(TeO_4)_2(C_4O_4)_4(OH)_{16}]^{8-}$				H_2O	EA	1538942	139
$[(Mo_2S_2O_2)_{14}(C_4O_4)_4(Te^{IV}O_4)_2(OH)_{20})]^{8-}$				H_2O	EA	1538939	139
$[(Mo_2O_2S_2)_{15}(TeO_4)_3(C_4O_4)_3O(OH)_{21}]^{11-}$				H_2O	EA	1538941	139
$[(Mo_6S_6O_{12})_4(C_4O_4)_4(CH_3COO)_2 Mo_{10}O_{28}]^{14-}$				H_2O	EA, IR	975305	136
$[(Mo_6S_6O_{12})_6(C_4O_4)_6Mo_{12}O_{34}]^{14-}$				H_2O	EA, IR	936797, 936798	136
$[(Mo_2O_2S_2)_{36}(Te_3O_{10})_4(C_4O_4)_{10}(OH)_{48}]^{28-}$				H_2O	EA	1538940	139
**Halogens (41 POMs)**							
$[V^{IV}{}_6O_6(OH)_3F((OCH_2)_3CCH_3)_3]^-$	Lindqvist	V^{IV}	F (μ_3-bridging)	hydrothermal in H_2O	CV, EA, IR	1171582	140
$[V^V{}_7V^{IV}{}_2O_{21}F(C_6H_4O_2N)_4]^{4-}$	Lindqvist (lacunary)	V^V	F (μ_5-bridging)	H_2O	EA, IR	1910707	141
Fluorinated metatungstates							
$[H_2W_{12}O_{39}F]^{5-}$	Keggin	W^{VI}	F (μ_3-bridging)	H_2O	EA, ^{1}H-, ^{19}F-NMR, UV-Vis	-	142
$[H_2W_{12}O_{38}F_2]^{4-}$					EA, ^{1}H-, ^{19}F-NMR, UV-Vis	-	142
$[HW_{12}O_{38}F_2]^{5-}$					CV, ^{19}F-NMR	-	142
$[HW_{12}O_{37}F_3]^{4-}$					CV, ^{19}F-NMR	-	142
$[H_2W_{11}O_{38}F\{Fe^{III}(H_2O)\}]^{6-}$					CV, EA, FAB-MS, IR, UV-Vis, XPD	-	143
$[H_2NaW_{11}O_{38}F\{Co^{II}(H_2O)\}]^{6-}$ originally described as: $[H_{10}NaW_{11}O_{42}F\{Co^{II}(H_2O)\}]^{6-}$, probably Keggin anion with disordered center					EA, IR, FAB-MS, UV-Vis, XPD	X-ray	144
$[H_2NaW_{11}O_{38}F\{Ni^{II}(H_2O)\}]^{6-}$ originally described as: $[H_{10}NaW_{11}O_{42}F\{Ni^{II}(H_2O)\}]^{6-}$, probably Keggin anion with disordered center					EA, FAB-MS, IR, UV-Vis, XPD	-	144
$[H_2W_{11}O_{37}F_2\{Al(H_2O)\}]^{5-}$	Keggin	W^{VI}	F (μ_3-bridging)	H_2O	CV, ^{19}F-NMR	-	142
$[H_4W_{11}O_{37}F_2\{Cu^{II}(H_2O)\}]^{4-}$					CV, EA, FAB-MS, IR, MSU, UV-Vis	-	145
$[H_4W_{11}O_{37}F_2\{Mn^{II}(H_2O)\}]^{4-}$					CV, EA, FAB-MS, IR, MSU, UV-Vis	-	145

$[H_2W_{11}O_{37}F_2\{Co^{II}(H_2O)\}]^{6-}$					CV, EA, FAB-MS, IR, UV-Vis, XPD	-	146
$[W_{11}O_{36}F_3\{Mn^{III}(OH)\}]^{7-}$					CV, EA, IR, FAB-MS, MSU, UV-Vis, XPD	-	147
$[H_2W_{11}O_{36}F_3\{Al(H_2O)\}]^{5-}$					CV, ^{19}F-NMR	-	142
$[ZnW^{VI}_{10}W^{V}O_{35}F_4\{Fe^{III}(OH)\}]^{5-}$					CV, EA, FAB-MS, IR, TGA, UV-Vis, XPD	-	148
Other fluorinated Keggin anions							
$[Co^{II}W_{11}O_{38}F\{Ni(H_2O)\}]^{7-}$	Keggin	W^{VI}	F (μ_3-bridging)	H_2O	CV, EA, FAB-MS, MSU, UV-Vis	X-ray	149
$[H_2Ni^{II}W_{11}O_{37}F_2\{Cu^{II}(H_2O)\}]^{4-}$					CV, EA, FAB-MS, IR, MSU, UV-Vis, XPD	-	150
$[H_2Mn^{II}W_{11}O_{37}F_2\{Ni^{II}(H_2O)\}]^{4-}$					CV, EA, FAB-MS, IR, MSU, UV-Vis, XPD	-	150
$[Zn^{II}W_{11}O_{37}F_2\{Cu^{II}(H_2O)\}]^{6-}$ (charge corrected by authors of this review)					CV, EA, FAB-MS, IR, TGA, UV-Vis, XPD	-	151
Fluorinated Wells–Dawson with various transition metal substitutions							
$[NaH_2W_{18}O_{56}F_6]^{7-}$	Wells-Dawson	W^{VI}	F (μ_3-bridging)	H_2O	EA, ^{1}H-, ^{19}F-, ^{23}Na-, ^{183}W-NMR, UV-Vis, UZ	X-ray	152, 153
$[NaH_2W_{17}VO_{56}F_6]^{8-}$					EA, ^{1}H-, ^{19}F-, ^{51}V-, ^{183}W-NMR, TGA	X-ray	154
**$[NaH_2W_{17}O_{55}F_6\{M(H_2O)\}]^{n-}$**							
M = Zn^{II}, n = 9–	Wells-Dawson	W^{VI}	F (μ_3-bridging)	H_2O	EA, ^{1}H-, ^{19}F-, ^{23}Na-, ^{183}W-NMR, UV-Vis	X-ray	153, 154
M = Co^{II}, n = 9–	Wells-Dawson	W^{VI}	F (μ_3-bridging)	H_2O	EA, FAB -MS, ^{1}H-, ^{19}F-, ^{23}Na-, ^{183}W-NMR, IR, UV-Vis, XPD	-	153, 154, 155

M = Co^{III}, n = 8–					EA, ^{1}H-, ^{19}F-, ^{23}Na-, ^{183}W-NMR, UV-Vis	-	153
M = Ni^{II}, n = 9–					EA, ^{1}H-, ^{19}F-, ^{23}Na-, ^{183}W-NMR, TGA, UV-Vis	X-ray	153, 154
M = Mn^{II}, n = 9–					EA, ^{1}H-, ^{19}F-, ^{23}Na-, ^{183}W-NMR, UV-Vis	-	153, 154
M = Mn^{III}, n = 8–					EA, ^{1}H-, ^{19}F-, ^{23}Na-, ^{183}W-NMR, UV-Vis	-	153
M = Fe^{II}, n = 9–					^{19}F-, ^{183}W-NMR	-	154
M = Fe^{III}, n = 8–					EA, FAB-MS, IR, UV-Vis, XPD	1632069	155
M = Ru^{II}, n = 9–					EA, ^{19}F-, ^{183}W-NMR, TGA	X-ray	154
Other degrees of fluorination							
$[NaH_4W_{17}O_{56}F_5\{Mg^{II}(H_2O)\}]^{8-}$	Wells-Dawson	W^{VI}	F (μ_3-bridging)	H_2O	EA, FAB -MS, IR, UV-Vis, XPD	-	156
$[NaH_3W_{17}O_{56}F_5\{Cu^{II}(H_2O)\}]^{9-}$					EA, FAB -MS, IR, UV-Vis, XPD	-	157
$[NaH_2W^{V}W^{VI}_{16}O_{54}F_7\{Fe^{III}(H_2O)\}]^{8-}$			F (μ_3- and μ_2-bridging)		EA, FAB -MS, IR, UV-Vis, XPD	-	156
$[NaH_2W_{17}O_{53}F_8\{Cu^{I}(H_2O)\}]^{8-}$					EA, FAB-MS, IR, UV-Vis, XPD	-	156
$[V^{III}_6Cl_6O((OCH_2)_3CCH_3)_4]^{2-}$	Lindqvist	V^{III}	Cl (terminal)	solvothermal in ACN	EA, MSU	757472	158
$[V^{III}_6Cl_6O((OCH_2)_3CC_2H_5)_4]^{2-}$					EA, MSU	757473	158
$[V^{III}V^{IV}_4V^{V}O_6Cl(OC_2H_5)_{12}]^{-}$	Lindqvist	V^{III}	Cl terminal	organic solvent	EA, SXRD, IR	1990062	159
$[V^{III}V^{IV}_3V^{V}_2O_6Cl(OC_2H_5)_{12}]$	Lindqvist	V^{III}	Cl terminal	organic solvent	EA, SXRD, IR	1990063	159
$[V^{III}V^{IV}_2V^{V}_3O_6Cl(OC_2H_5)_{12}]^{+}$	Lindqvist	V^{III}	Cl terminal	organic solvent	EA, SXRD, IR	1990064	159
A,β–$[PW_9O_{28}Br_6]^{3-}$	Keggin	W^{VI}	Br (terminal)	anhydrous ACN under N_2	IR, ^{31}P-, ^{183}W-NMR	139046	160

2. Amino, imino and amido ligands – stabilizing agents for labile structures

When O_t atoms are replaced by an organic amine (NR_3), imine ($R\text{-}N{=}CR_2$) or amide (R-$CONH_2$), the resulting amino functionalization is largely considered to be a *σ*-bond with weak *π*-contribution, and therefore less stable than a pronounced multiple bond interaction. Thus, only POM structures with suitable addenda centers that do not require further stabilization by electron donation through the ligand were found with this kind of substitution. Such addenda atoms require an increased electron density as provided by either reduction or in lacunary anions addenda atoms with charged *cis*-dioxo sites (cf. Fig. 1D-H).

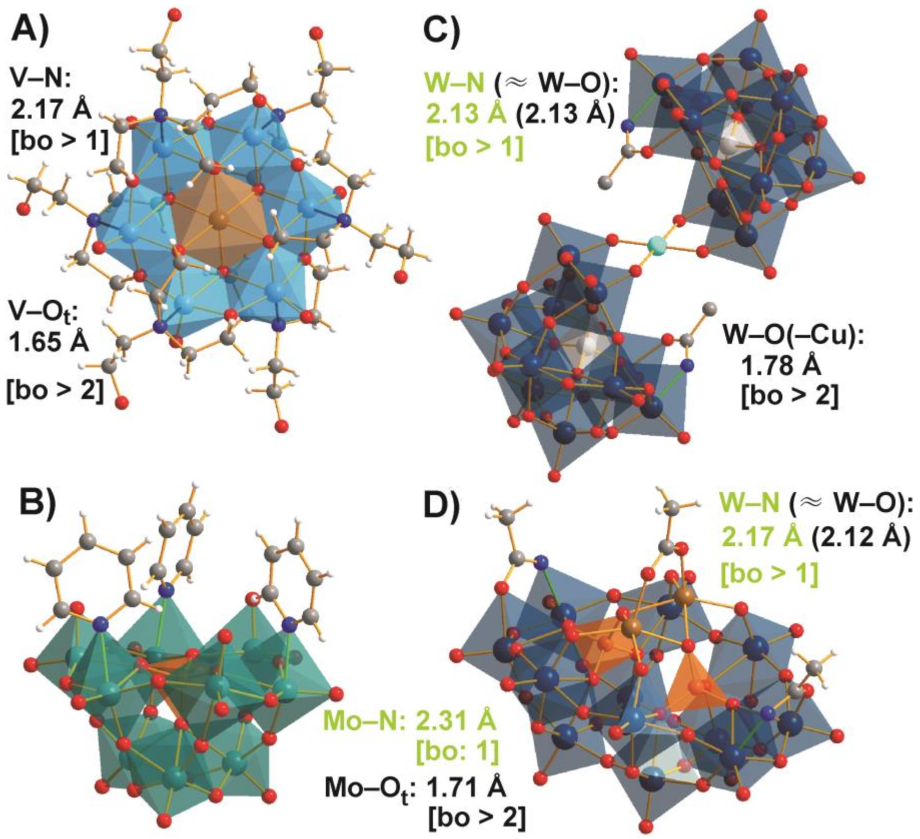

Fig. S1: POMs of various archetypes substituted with singly bound nitrogen ligands in a mixed ball-and-stick and polyhedral representation. **A**) Representative Anderson-Evans-like cation $[Fe^{II}V^{IV}_6O_6\{(OCH_2CH_2)_3N\}_6]^{2+}$ (CCDC: 216908[9]). **B**) Trivacant Keggin anion *A*-*α*-$[(P^VMo^{VI}_9O_{31})(NC_5H_5)_3]^{3-}$ (CCDC: 1899175[11]). **C**) Dilacunary *γ*-Keggin unit in $[(Si^{IV}W^{VI}_{10}O_{36}(ONHCCH_3))_2Cu^{II}]^{8-}$ (CCDC: 912546[12]). **D**) Representative hexavacant *γ*-Wells-Dawson fragment $[(P^V_2W^{VI}_{12}O_{44}(ONHCCH_3)_2Mn^{II}_2(CH_3CO_2)]^{3-}$ (CCDC: 1883422[16]). All metal-nitrogen bonds are essentially single bonds, while free oxo groups retain their multiple bonding interaction. Color code: light blue, V^{IV}; brown, Fe^{II} or Mn^{II}; dark blue, W^{VI}; light grey, Si; orange, P; dark green, Mo^{VI}; red, O; blue, N; dark grey, C; white, H; cyan, Cu^{II}. The

relevant bonds are highlighted in color and their lengths are compared. Suggested bond orders (bo) are given in square brackets.

Highly reactive Keggin-type trilacunary anions (Fig. S1B) were stabilized by spontaneous functionalization with pyridine ligands in organic media[11], where they otherwise rearrange to structures such as the monolacunary or intact forms to achieve a lower charge density. The high charge density of the $[A\text{-}\alpha\text{-}P^{V}Mo^{VI}_{9}O_{34}]^{9-}$ lacuna site was reduced by replacing three oxo-ligands with pyridyl functions, respectively, with very long bonds to the metal centers (Fig. S1B). For various lacunary POT scaffolds derived from Keggin[12,17] and Wells-Dawson[16] archetypes, an unexpected phenomenon occurred during the crystallization from acetonitrile: the solvent covalently attached to the POTs by an addition-condensation reaction ended up as an acetamido ligand bridging two neighboring W centers. In addition to this stabilizing interaction, transition metals were introduced to a dilacunary Keggin (Fig. S1C) and a Wells-Dawson fragment, which was obtained in an unusual *γ*-form (Fig. S1D).

Khan *et al.*[8] prepared a series of cationic polyoxovanadates featuring the Anderson-Evans scaffold (Fig. S1A) from fully oxidized decavanadate $[V^{V}_{10}O_{28}]^{6-}$ with an excess of tris-ethanolamine under solvothermal conditions. The obtained structures (Fig. S1A) comprise a neutral $[V^{IV}_{6}O_{6}\{(OCH_2CH_2)_3N\}_6]$ cage centered by various mono- and divalent cations, with the reduced V^{IV} centers being bridged by the amine ligand. Even the oxidation-sensitive Fe^{II} can be stabilized in aqueous solution enclosed in this complex.

3. An alternative binding-mode for oxygen - the peroxo modification

In the form of the peroxo ligand O_2^{2-}, oxygen can replace terminal O_t sites in a side-on η^2-coordination mode, providing an appropriate electronic configuration for a six-electron donation to the metal addenda atom (Fig. 1A), which would not be possible through end-on binding.

In an ESI-MS study of POMos in the presence of hydrogen peroxide, the Lindqvist hexamolybdate anion was found with up to four O_t sites to be transiently replaced by a peroxo ligand[119]. However, the only available crystal structure of a POM modified in this way is a Lindqvist polyoxoniobate (Fig. S2A) investigated by Ohlin *et al.*[113].

Consistent with the d orbital interaction, the O-O axis of the ligands is always parallel to one of the two O-Nb-O axes of a $\{NbO_5(O_2)\}$ unit. The bond lengths correspond to a strong ligand interaction with the metal, leaving the single bond linking the peroxo atoms unaffected[161]. The η^2-peroxo ligand generally exhibits a structural *trans*-influence very similar to the oxo-group it replaces[162].

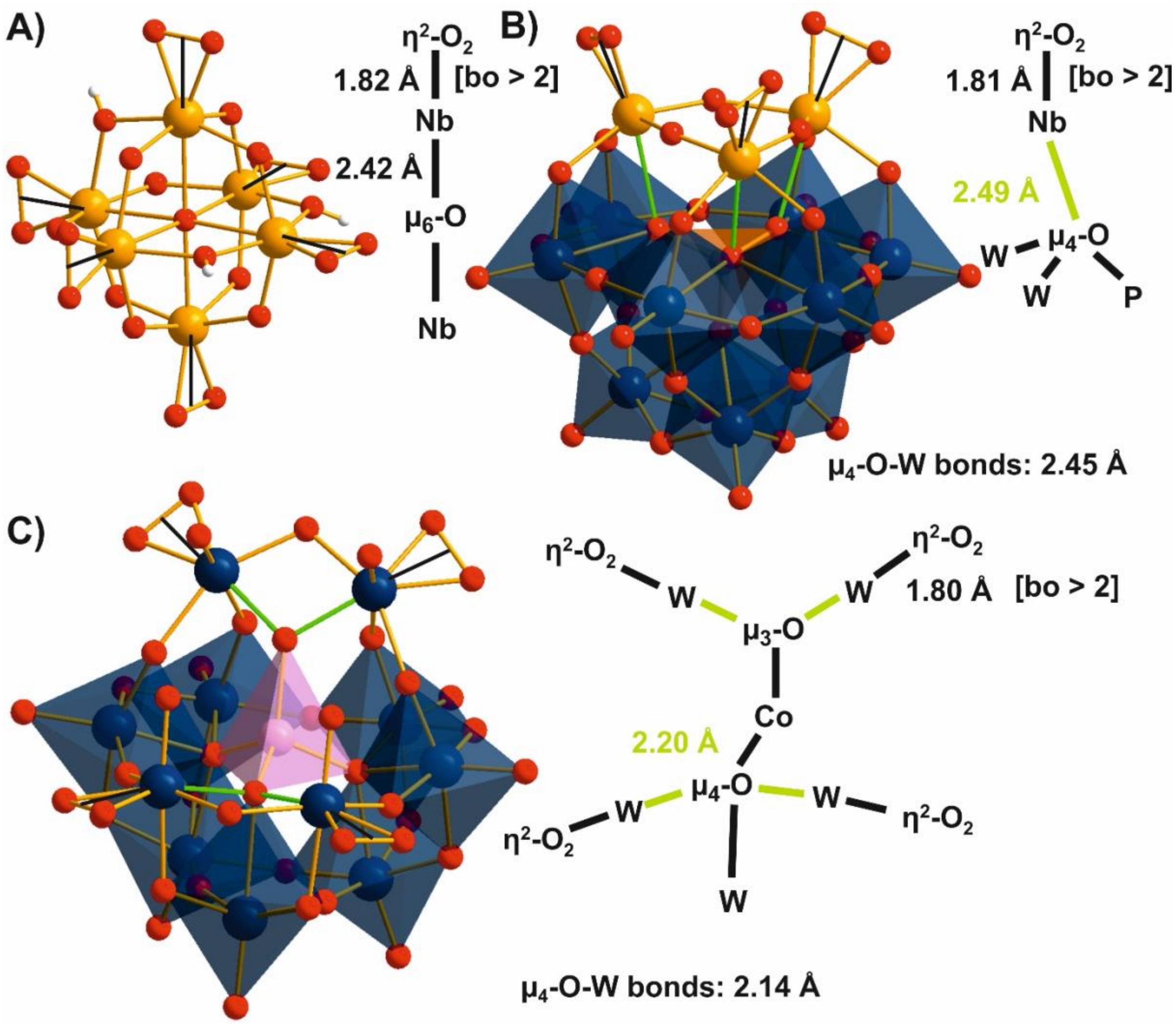

Fig. S2: POMs with peroxo ligands in a mixed ball-and-stick and polyhedral representation. **A**) Lindqvist anion $H_3[Nb^V{}_6O_{13}(O^I{}_2)_6]^{5-}$ (CCDC: 710790[113]). **B**) Keggin structure $[P^VW^{VI}{}_9O_{37}\{Nb^V(O^I{}_2)\}_3]^{6-}$ (CCDC: 1645702[114]). **C**) Highly charged lacunary Keggin structure $[Co^{II}W^{VI}{}_{11}O_{35}(O^I{}_2)_4]^{10-}$ (CCDC: 1647800[120]). The linkage to the terminal η^2-peroxo ligands has a strong multiple bond character, consistent with the nearly unchanged bonds to the μ_6-O center and a strong *trans*- influence comparable to the oxo-ligand O^{2-}. Color code: light orange, Nb^{VI}; dark blue, W^{VI}; orange, P; rose, Co; red, O; white, H. Relevant bonds are emphasized by colours and compared for their lengths. Suggested bond orders (bo) are given in square brackets.

By assessing the time-dependent solution chemistry of the Lindqvist hexaniobate and decaniobate structures in the presence of peroxide ions using a combined ESI-MS and ^{17}O-NMR approach, it was revealed that the decaniobate anion can be substituted by a maximum number of two terminal peroxo ligands before its decomposition to the Lindqvist archetype. The decaniobate peroxo modification occurs rapidly within a few minutes, but is lost over time, therefore no crystal structure could be obtained. The peroxo substitution of the smaller hexaniobate requires hours, but is remarkably stable. A transient increase of the degree of substitution was observed in a reaction with six equivalents of hydrogen peroxide, covering the

whole range from mono- to hexaperoxo species with a clear preference for even numbers of peroxo ligands.

The reactivity of Nb^{V} addenda centers for the peroxo replacement was also exploited to obtain the Keggin structures *A*-*β*-$[Si^{IV}W^{VI}_9O_{37}\{Nb^{V}O_2\}_3]^{7-}$ [116] and *A*-*β*-$[P^{V}W^{VI}_9O_{37}\{Nb^{V}O_2\}_3]^{6-}$ [114] (Fig. S2B) as well as the Wells-Dawson structure $[P^{V}_2W^{VI}_{12}O_{56}\{Nb^{V}O_2\}_6]^{12-}$ [118]. A high concentration of free peroxide was necessary to stabilize these compounds in solution and to enable – in some cases – their crystallization, but the principal synthetic accessibility of other Nb-substituted peroxo-POMs was demonstrated. Interestingly, the Keggin silicotungstate is synthetically accessible by three routes[116]: 1) the cleavage of the sandwich dimer *A*-*β*-$[(Si^{IV}W^{VI}_9Nb^{V}_3O_{37})_2(\mu_2\text{-}O)_3]^{8-}$ by peroxide, 2) the *in situ* completion of the trilacunary Keggin precursor *A*-*β*-$[Si^{IV}W^{VI}_9O_{34}]^{10-}$ with Nb^{V} centers from Lindqvist hexaniobate in the presence of peroxide, or 3) the direct oxo replacement of the preformed Keggin structure *A*-*β*-$[Si^{IV}W^{VI}_9Nb^{V}O_{40}]^{7-}$ by O_2^{2-}. The peroxo-substituted Keggin POTs were studied as catalysts for alkene epoxidation reactions. Although the reaction conditions (acidic pH in the presence of H_2O_2) were close to those of the POT synthesis, the originally applied polyoxometalates did not survive the alkene oxidation and decomposed to smaller POT species, with the simple orthotungstate system WO_4^{2-}/H_2O_2 exhibiting the highest activity[113]. However, the high charge density of *A*-*β*-$[Si^{IV}W^{VI}_9O_{37}\{Nb^{V}O_2\}_3]^{7-}$ (q/m = 0.58) was proposed to provide sufficiently nucleophilic oxygen atoms to catalyze the observed epoxide ring opening to the final diol products. The same anion was also investigated within a series of Keggin POTs as potential anti-HIV agents[115]. Although the antiviral activity correlated with the degree of $\{Nb(O_2)\}$ substitution, the design of the study did not allow a clear assignment of the observed efficacy to the peroxide content or to the increase in charge density through the substitution of W^{VI} addenda metals for Nb^{V}. As recognized by the authors, it is the relatively high charge density that preserves the structural integrity of these POMs in the physiological pH milieu applied for biological studies. Similar to the Keggin compounds, the Wells-Dawson POT $[P^{V}_2W^{VI}_{12}O_{56}\{Nb^{V}O_2\}_6]^{12-}$ has been reported to exhibit anti-HIV activity with an IC_{50} value in the nM range[118], and the monosubstituted compounds α_1- and α_2-$[P^{V}_2W^{VI}_{17}O_{61}\{Nb^{V}O_2\}]^{7-}$ showing a ten-fold reduced inhibition[117], but the comparison to oxo-POMs with the same charge density is missing.

Notably, the Acerete group reported the first peroxo Keggin POT derivative (Fig. S2C) with η^2-O_2^{2-} ligands attached to W addenda centers[120] by peroxide reduction of the Co^{III}-centered lacunary anion $[Co^{III}W^{VI}_{11}O_{39}]^{9-}$. The very high charge density on the *cis*-dioxo tungsten atoms in this anion activated the O_t sites for replacement, but only those pointing away from the lacuna. As observed for Nb^{V} addenda metals, the O_2^{2-}-substitution leaves the whole POT framework unaffected. The peroxo-substituted Keggin POT was demonstrated as an efficient

olefin epoxidation and alcohol oxidation catalyst, and established this class of compounds for more catalytic applications.

4. Other POM structures based on the {$M_2S_2O_2$} subunit

Cadot *et al.* were the first to recognize that the $[M^{V}_2S_2O_2(H_2O)_6]^{2+}$ building block can be combined with lacunary POM anions comprising vacant sites of appropriate size to accommodate this fragment. The group also synthesized various sandwich compounds based on mono-[126,127] and trilacunary[125] Keggin POT structures, whose defect sites could not be recomplemented by the {$M^{V}_2S_2O_2$} subunit in a 1:1 ratio.

Notably, the reversible self-condensation of the cationic building-block $[M^{V}_2S_2O_2(H_2O)_6]^{2+}$ under basic conditions gives rise to an interesting distinct family of cyclic polyoxothiometalates of the uncharged structure $[M^{V}_2S_2O_2(OH)_2(H_2O)]_n$, where the addenda metal M is mostly Mo[134,137]. The size and charge of the neutral metalate rings formed in this way can be controlled by the incorporation of inorganic [128,131,132] and organic[130] anionic templates in acidic media (see Table S1 for crystal structures). Since no corresponding oxo structures are available for comparison, these structures have generally not been considered for further discussion in this review.

5. References

[1] F. Bottomley, J. Chen. Organometallic oxides: oxidation of $[(\eta\text{-}C_5Me_5)Mo(CO)_2]_2$ with O_2 to form *syn*-$[(\eta\text{-}C_5Me_5)MoCl]_2(\mu\text{-}Cl)_2(\mu\text{-}O)$, *syn*-$[(\eta\text{-}C_5Me_5)MoCl]_2(\mu\text{-}Cl)(\mu\text{-}CO_3H)(\mu\text{-}O)$, and $[C_5Me_5O][(\eta\text{-}C_5Me_5)Mo_6O_{18}]$. *Organometallics* 11 (1992) 3404–3411. https://doi.org/10.1021/om00046a045.

[2] A. Proust, R. Thouvenot, P. Herson. Revisiting the synthesis of $[Mo_6(\eta^5\text{-}C_5Me_5)O_{18}]^-$. X-ray structural analysis, UV-visible, electrochemical and multinuclear NMR characterization. *J. Chem. Soc. Dalton Trans.* (1999) 51–56. https://doi.org/10.1039/a805832f.

[3] C. Bhaumik, E. Manoury, J.-C. Daran, P. Sözen-Aktaş, F. Demirhan, R. Poli. Investigation of the reaction of $[Cp^*{}_2M_2O_5]$ (M = Mo, W) with hydrogen peroxide and tert-butylhydroperoxide in MeCN; implications for olefin epoxidation catalyzed by organomolybdenum and organotungsten compounds. *J. Organomet. Chem.* 760 (2014) 115–123. https://doi.org/10.1016/j.jorganchem.2013.11.029.

[4] G. Taban-Çalışkan, D. Mesquita Fernandes, J.-C. Daran, D. Agustin, F. Demirhan, R. Poli. Rational Synthesis and Characterization of the Mixed-Metal Organometallic Polyoxometalates $[Cp^*Mo_xW_{6-x}O_{18}]^-$ (x = 0, 1, 5, 6). *Inorg. Chem.* 51 (2012) 5931–5940. https://doi.org/10.1021/ic300578g.

[5] E. Collange, L. Metteau, P. Richard, R. Poli. Synthesis and structure of a new organometallic polyoxomolybdate. *Polyhedron* 23 (2004) 2605–2610. https://doi.org/10.1016/j.poly.2004.06.025.

[6] J. R. Harper, A. L. Rheingold. Arsaoxanes as reversible, ligating oxygen-transfer agents in the synthesis of neutral metal-oxo clusters. The X-ray structures of $Cp*_2W_6O_{17}$ and $Cp*_6Mo_8O_{16}$. *J. Am. Chem. Soc.* 112 (1990) 4037–4038. https://doi.org/10.1021/ja00166a050.

[7] B. E. Petel, A. A. Fertig, M. L. Maiola, W. W. Brennessel, E. M. Matson. Controlling metal-to-oxygen ratios via M═O bond cleavage in polyoxovanadate alkoxide clusters. *Inorg. Chem.* 58 (2019) 10462–10471. https://doi.org/10.1021/acs.inorgchem.9b00389.

[8] M. I. Khan, Y. Zheng, H. Li, L. Swenson, A. Basha, R. J. Doedens. Organo-functionalized metal–oxide clusters: synthesis and characterization of the reduced cationic species $[NaV^{IV}_6O_6\{(OCH_2CH_2)_2NH\}_6]^+$. *Dalton Trans.* 43 (2014) 16509–16514. https://doi.org/10.1039/C4DT02174F.

[9] M. I. Khan, S. Tabussum, R. J. Doedens, V. O. Golub, C. J. O'Connor. Functionalized metal oxide clusters: Synthesis, characterization, crystal structures, and magnetic properties of a novel series of fully reduced heteropolyoxovanadium cationic clusters decorated with organic ligands–$[M^{VI}V_6O_6\{(OCH_2CH_2)_2N(CH_2CH_2OH)\}_6]X$ (M = Li, X = Cl·LiCl; M = Na, X = Cl·H_2O; M = Mg, X = 2Br·H_2O; M = Mn, Fe, X = 2Cl; M = Co, Ni, X = 2Cl·H_2O). *Inorg. Chem.* 43 (2004) 5850–5859. https://doi.org/10.1021/ic049417m.

[10] L. Huang, X. Liu, J. Zhou, H. Zou, D. Wen. One octasubstituted trisalkoxotetradecavanadate cluster, *Inorg. Chem.* 60 (2021) 14–18. https://doi.org/10.1021/acs.inorgchem.0c03089.

[11] C. Li, N. Mizuno, K. Yamaguchi, K. Suzuki. Self-assembly of anionic polyoxometalate–organic architectures based on lacunary phosphomolybdates and pyridyl ligands. *J. Am. Chem. Soc.* 141 (2019) 7687–7692. https://doi.org/10.1021/jacs.9b02541.

[12] K. Suzuki, M. Shinoe, N. Mizuno. Synthesis and reversible transformation of Cu_n-bridged (n = 1, 2, or 4) silicodecatungstate dimers. *Inorg. Chem.* 51 (2012) 11574–11581. https://doi.org/10.1021/ic301488a.

[13] C. Li, K. Yamaguchi, K. Suzuki. Ligand-directed approach in polyoxometalate synthesis: formation of a new divacant lacunary polyoxomolybdate $[\gamma\text{-}PMo_{10}O_{36}]^{7-}$. *Angew*. *Chem*. *Int*. *Ed*. 60 (2021) 6960–6964. https://doi.org/10.1002/anie.202016642.

[14] C. Ren, Z. Lu, B. Luo, X. Yi, L. Lin, L. Xu. Deeply reduced empty Keggin clusters $[Mo^{IV}_xM^{VI}_{12-x}O_{40-x}Py_x]$ (x = 3, 6; M = Mo, W; Py = Pyridine): Synthesis, structures, and Lewis field catalysis. *Inorg. Chem. Front*. 10 (2021) 1039–1046. https://doi.org/10.1039/D1QI01080H.

[15] R. L. Meyer, P. Miró, W. W. Brennessel, E. M. Matson. O_2 activation with a sterically encumbered, oxygen-deficient polyoxovanadate-alkoxide cluster. *Inorg. Chem*. 60 (2021), 13833–13843. https://doi.org/10.1021/acs.inorgchem.1c00887.

[16] K. Suzuki, T. Minato, N. Tominaga, I. Okumo, K. Yonesato, N. Mizuno, K. Yamaguchi. Hexavacant γ-Dawson-type phosphotungstates supporting an edge-sharing bis(square-pyramidal) $\{O_2M(\mu_3\text{-}O)_2(\mu\text{-}OAc)MO_2\}$ core (M = Mn^{2+}, Co^{2+}, Ni^{2+}, Cu^{2+}, or Zn^{2+}). *Dalton Trans.* 48 (2019) 7281–7289. https://doi.org/10.1039/C8DT04850A.

[17] J. Jeong, K. Suzuki, K. Yamaguchi, N. Mizuno. Visible-light-responsive catalysis of a zinc-introduced lacunary disilicoicosatungstate for the deoxygenation of pyridine *N*-oxides. *New J. Chem.* 41 (2017) 13226–13229. https://doi.org/10.1039/C7NJ03057F.

[18] Q. Li, L. Wang, P. Yin, Y. Wei, J. Hao, Y. Zhu, L. Zhu, G. Yuan. Convenient syntheses and structural characterizations of mono-substituted alkylimido hexamolybdates: $[Mo_6O_{18}(NR)]^{2-}$ (R = Me, Et, n-Pr, i-Pr, n-Bu, t-Bu, Cy, Hex, Ode). *Dalton Trans.* (2009) 1172–1179. https://doi.org/10.1039/B813924E.

[19] Q. Li, J. Zhang, L. Wang, J. Hao, P. Yin, Y. Wei. Nucleophilic substitution reaction for rational post-functionalization of polyoxometalates. *New J. Chem.* 40 (2016) 906–909. https://doi.org/10.1039/C5NJ01090J.

[20] J. B. Strong, G. P. A. Yap, R. Ostrander, L. M. Liable-Sands, A. L. Rheingold, R. Thouvenot, P. Gouzerh, E. A. Maatta. A new class of functionalized polyoxometalates: synthetic, structural, spectroscopic, and electrochemical studies of organoimido derivatives of $[Mo_6O_{19}]^{2-}$. *J. Am. Chem. Soc.* 122 (2000) 639–649. https://doi.org/10.1021/ja9927974.

[21] A. Proust, R. Thouvenot, M. Chaussade, F. Robert, P. Gouzerh. Phenylimido derivatives of $[Mo_6O_{19}]^{2-}$: syntheses, X-ray structures, vibrational, electrochemical, ^{95}Mo and ^{14}N NMR studies. *Inorg. Chim. Acta* 224 (1994) 81–95. https://doi.org/10.1016/0020-1693(94)04110-5.

[22] C. Lv, J. Hu, R. N. N. Khan, J. Zhang, Y. Wei. Postfunctionalization of polyoxometalates: an efficient strategy to construct organic–inorganic zwitterions. *Dalton Trans.* 44 (2015) 16698–16702. https://doi.org/10.1039/C5DT02407B.

[23] S. Xue, C. Xiang, Y. Wei, Z. Tao, A. Chai, W. Bian, Z. Xu. Synthesis, crystal structure, spectroscopic, and herbicidal activity studies of a series of designed fluoro-functionalized phenylimido derivatives of hexametalate cluster. *Cryst. Growth Des.* 8 (2008) 2437–2443. https://doi.org/10.1021/cg8000174.

[24] Q. Li, Y. Wei, H. Guo, C.-G. Zhan. Syntheses, structural characterizations and electronic absorption spectra simulation of three phenylimido substituted hexamolybdates incorporating a remote chloro group. *Inorg. Chim. Acta* 361 (2008) 2305–2313. https://doi.org/10.1016/j.ica.2007.11.031.

[25] P. Wu, Q. Li, N. Ge, Y. Wei, Y. Wang, P. Wang, H. Guo. An easy route to monofunctionalized organoimido derivatives of the lindqvist hexamolybdate. *Eur. J. Inorg. Chem.* 2004 (2004) 2819–2822. https://doi.org/10.1002/ejic.200400168.

[26] Q. Li, P. Wu, Y. Xia, Y. Wei, H. Guo. Synthesis, spectroscopic studies and crystal structure of a polyoxoanion cluster incorporating *para*-bromophenylimido ligand, $(Bu_4N)_2[Mo_6O_{18}(NC_6H_4Br\text{-}p)]$. *J. Organomet. Chem.* 691 (2006) 1223–1228. https://doi.org/10.1016/j.jorganchem.2005.11.071.

[27] A. Al-Yasari, N. Van Steerteghem, H. Kearns, H. El Moll, K. Faulds, J. A. Wright, B. S. Brunschwig, K. Clays, J. Fielden. Organoimido-polyoxometalate nonlinear optical chromophores: a structural, spectroscopic, and computational study. *Inorg. Chem.* 56 (2017) 10181–10194. https://doi.org/10.1021/acs.inorgchem.7b00708.

[28] Q. Li, P. Wu, Y. Wei, Y. Xia, Y. Wang, H. Guo. Organic-inorganic hybrids: preparation and structural characterization of $(Bu_4N)_2[Mo_6O_{17}(NAr)_2]$ and $(Bu_4N)_2[Mo_6O_{18}(NAr)]$ (Ar = o-$CH_3C_6H_4$). *Z. Anorg. Allg. Chem.* 631 (2005) 773–779. https://doi.org/10.1002/zaac.200400351.

[29] Y. Du, A. L. Rheingold, E. A. Maatta. A polyoxometalate incorporating an organoimido ligand: preparation and structure of $[Mo_5O_{18}(MoNC_6H_4CH_3)]^{2-}$. *J. Am. Chem. Soc.* 114 (1992) 345–346. https://doi.org/10.1021/ja00027a046.

[30] H. Kwen, A. M. Beatty, E. A. Maatta. A *p*-cyanophenylimido hexamolybdate: preparation and structure of $[(n\text{-}C_4H_9)_4N]_2[Mo_6O_{18}(N\text{-}p\text{-}C_6H_4CN)]$. *C. R. Chimie* 8 (2005) 1025–1028. https://doi.org/10.1016/j.crci.2005.01.003.

[31] H. El Moll, F. A. Black, C. J. Wood, A. Al-Yasari, A. Reddy Marri, I. V. Sazanovich, E. A. Gibson, J. Fielden. Increasing p-type dye sensitised solar cell photovoltages using polyoxometalates. *Phys. Chem. Chem. Phys.* 19 (2017) 18831–18835. https://doi.org/10.1039/C7CP01558E.

[32] Q. Li, P. Yin, L. Shi, Y. Wei. Syntheses, Crystal structures, and spectroscopic studies of aromatic ester derivatives of hexamolybdate. *Eur. J. Inorg. Chem.* 2009 (2009) 5227–5232. https://doi.org/10.1002/ejic.200900594.

[33] Y. Zhu, P. Yin, F. Xiao, D. Li, E. Bitterlich, Z. Xiao, J. Zhang, J. Hao, T. Liu, Y. Wang, Y. Wei. Bottom-up construction of POM-based macrostructures: coordination assembled paddle-wheel macroclusters and their vesicle-like supramolecular aggregation in solution. *J. Am. Chem. Soc.* 135 (2013) 17155–17160. https://doi.org/10.1021/ja408228b.

[34] Q. Li, Z. Xiao, L. Chen, J. Zhang. A new mono-functionalized organoimido hexamolybdate derivative: bis(tetra-*n*-butylammonium) (5-chloro-2-methylphenylimido)-μ_6-oxido-dodeca-μ_2-oxido-pentaoxidohexamolybdate(VI). *Acta Cryst.* E67 (2011) m1404–m1405. https://doi.org/10.1107/S1600536811036063.

[35] Q. Li, L. Zhu, X. Meng, Y. Zhu, J. Hao, Y. Wei. Two new bromo-functionalized organoimido derivatives of hexamolybdate: Synthesis, crystal structure, spectroscopic and electrochemical studies. *Inorg. Chim. Acta* 360 (2007) 2558–2564. https://doi.org/10.1016/j.ica.2006.12.039.

[36] S. Xue, A. Chai, Y. Wei, C. Xiang, W. Bian, J. Shen. Two novel mono-organoimido functionalized polyoxometalate clusters: Convenient synthesis, crystal structure and bioactivity of $[(n\text{-}C_4H_9)_4N]_2[Mo_6O_{18}(NAr)]$ (Ar = *o*-$CF_3C_6H_4$, *p*-$OCF_3C_6H_4$). *J. Mol. Struct.* 888 (2008) 300–306. https://doi.org/10.1016/j.molstruc.2007.12.035.

[37] Y. Zhu, W. Yan, Y. Zhang. Synthetic, structural and spectroscopic studies on mono- and bi-arylimido functionalized hexamolybdates bearing the strong electron-withdrawing nitro group, $(Bu_4N)_2[Mo_6O_{18}(\equiv NAr)]$ and $(Bu_4N)_2[Mo_6O_{17}(\equiv NAr)_2]$ (Ar=o-NO_2–C_6H_4). *J. Organomet. Chem.* 696 (2011) 1850–1855. https://doi.org/10.1016/j.jorganchem.2011.02.020.

[38] P. Hermosilla-Ibáñez, K. Wrighton-Araneda, W. Cañón-Mancisidor, M. Gutiérrez-Cutiño, V. Paredes-García, D. Venegas-Yazigi. Substitution effect on the charge transfer processes in organo-imido Lindqvist-polyoxomolybdate. *Molecules* 24 (2018) 44. https://doi.org/10.3390/molecules24010044.

[39] P. Hermosilla-Ibáñez, K. Wrighton-Araneda, G. Prado, V. Paredes-García, N. Pizarro, A. Vega, D. Venegas-Yazigi. The first Re^I organometallic complex with an organoimido-polyoxometalate ligand. *Dalton Trans.* 46 (2017) 8611–8620. https://doi.org/10.1039/C7DT01633F.

[40] Y. Zhu, Z. Xiao, L. Wang, P. Yin, J. Hao, Y. Wei, Y. Wang. Monosubstituted arylimido hexamolybdates containing pendant amino groups: synthesis and structural characterization. *Dalton Trans.* 40 (2011) 7304–7309. https://doi.org/10.1039/c1dt10168d.

[41] H. Guo, S.-Z. Li, X.-X. Xiong, D. Li, Z.-Y. Liu, L.-S. Wang, P.-F. Wu. Syntheses, structures and characterizations of two organoimido derivatives of POMs containing sulfide groups. *Polyhedron* 92 (2015) 1–6. https://doi.org/10.1016/j.poly.2015.02.010.

[42] H. Yu, S. Le, X. Zeng, J. Zhang, J. Xie. Facile synthesis of a novel mono-organoimido functionalized polyoxometalate cluster $[(n\text{-}C_4H_9)_4N]_2[Mo_6O_{18}(\equiv NAr)]$ (Ar = *p*-$C_2H_5C_6H_4$): Crystal structure, spectral characterization and initial antitumor activity. *Inorg. Chem. Commun.* 39 (2014) 135–139. https://doi.org/10.1016/j.inoche.2013.11.001.

[43] A. R. Moore, H. Kwen, A. M. Beatty, E. A. Maatta. Organoimido-polyoxometalates as polymer pendants. *Chem. Commun.* 18 (2000) 1793–1794. https://doi.org/10.1039/b005022i.

[44] A. Al-Yasari, N. Van Steerteghem, H. El Moll, K. Clays, J. Fielden. Donor–acceptor organo-imido polyoxometalates: high transparency, high activity redox-active NLO chromophores. *Dalton Trans.* 45 (2016) 2818–2822. https://doi.org/10.1039/C6DT00115G.

[45] A. Al-Yasari, P. Spence, H. El Moll, N. Van Steerteghem, P. N. Horton, B. S. Brunschwig, K. Clays, J. Fielden. Fine-tuning polyoxometalate non-linear optical chromophores: a molecular electronic "Goldilocks" effect. *Dalton Trans.* 47 (2018) 10415–10419. https://doi.org/10.1039/C8DT01491D.

[46] Y. Wei, B. Xu, C. L. Barnes, Z. Peng. An efficient and convenient reaction protocol to organoimido derivatives of polyoxometalates. *J. Am. Chem. Soc.* 123 (2001) 4083–4084. https://doi.org/10.1021/ja004033q.

[47] Z. Xiao, Y. Zhu, Y. Wei, Y. Wang. Synthesis and characteristic of a new arylimido derivative of hexamolybdate with remote strong electro-donating group $(Bu_4N)_2[Mo_6O_{18}NC_6H_4N(CH_3)_2$-*p*]. *Inorg. Chem. Commun.* 9 (2006) 400–402. https://doi.org/10.1016/j.inoche.2006.01.009.

[48] R. N. Nasim Khan, N. Mahmood, C. Lv, G. Sima, J. Zhang, J. Hao, Y. Hou, Y. Wei. Pristine organo-imido polyoxometalates as an anode for lithium ion batteries. *RSC Adv.* 4 (2014) 7374. https://doi.org/10.1039/c3ra46645k.

[49] M. Healey, S. Best, L. Goerigk, C. Ritchie. A heteroaromatically functionalized hexamolybdate. *Inorganics* 3 (2015) 82–100. https://doi.org/10.3390/inorganics3020082.

[50] Y. Zhu, L. Wang, J. Hao, P. Yin, J. Zhang, Q. Li, L. Zhu, Y. Wei. Palladium-catalyzed Heck reaction of polyoxometalate-functionalised aryl iodides and bromides with olefins. *Chem. Eur. J.* 15 (2009) 3076–3080. https://doi.org/10.1002/chem.200802509.

[51] B. Xu, Y. Wei, C. L. Barnes, Z. Peng. Hybrid molecular materials based on covalently linked inorganic polyoxometalates and organic conjugated systems. *Angew. Chem. Int. Ed.* 40 (2001) 2290–2292. https://doi.org/10.1002/1521-3773(20010618)40:12<2290::AID-ANIE2290>3.0.CO;2-P.

[52] B. Xu, M. Lu, J. Kang, D. Wang, J. Brown, Z. Peng. Synthesis and optical properties of conjugated polymers containing polyoxometalate clusters as side-chain pendants. *Chem. Mater.* 17 (2005) 2841–2851. https://doi.org/10.1021/cm050188r.

[53] J. Kang, J. A. Nelson, M. Lu, B. Xie, Z. Peng, D. R. Powell. Charge-transfer hybrids containing covalently bonded polyoxometalates and ferrocenyl units. *Inorg. Chem.* 43 (2004) 6408–6413. https://doi.org/10.1021/ic049250x.

[54] S. She, Z. Huang, P. Yin, A. Bayaguud, H. Jia, Y. Huang, Y. Wei, Y. Wei. Buildup of redox-responsive hybrid from polyoxometalate and redox-active conducting oligomer: its self-assemblies with controllable morphologies. *Chem. Eur. J.* 23 (2017) 14860–14865. https://doi.org/10.1002/chem.201702857.

[55] B. Xu, Z. Peng, Y. Wei, D. R. Powell. Polyoxometalates covalently bonded with terpyridine ligands. *Chem. Commun.* 9 (2003) 2562-2563. https://doi.org/10.1039/b307415c.

[56] R. Jangir, R. Antony, R. Murugavel. New sterically encumbered arylimido hexamolybdates for organic oxidation reactions. *New J. Chem.* 40 (2016) 1004–1013. https://doi.org/10.1039/C5NJ02330K.

[57] S. She, S. Bian, J. Hao, J. Zhang, J. Zhang, Y. Wei. Aliphatic organoimido derivatives of polyoxometalates containing a bioactive ligand. *Chem. Eur. J.* 20 (2014) 16987–16994. https://doi.org/10.1002/chem.201404317.

[58] J. L. Stark, V. G. Young, E. A. Maatta. A functionalized polyoxometalate bearing a ferrocenylimido ligand: preparation and structure of $[(FcN)Mo_6O_{18}]^{2-}$. *Angew. Chem. Int. Ed.* 34 (1995) 2547–2548. https://doi.org/10.1002/anie.199525471.

[59] Y. Zhu, Z. Xiao, N. Ge, N. Wang, Y. Wei, Y. Wang. Naphthyl amines as novel organoimido ligands for design of POM-based organic−inorganic hybrids: synthesis, structural characterization, and supramolecular assembly of $(Bu_4N)_2[Mo_6O_{18}N(Naph\text{-}1)]$. *Cryst. Growth Des.* 6 (2006) 1620–1625. https://doi.org/10.1021/cg0600694.

[60] Q. Liu, L. Hu, H. Fu, J. Yang, Q.-M. Fu, L. Liu, S.-Z. Liu, Z.-L. Du, C.-L. Ho, F.-R. Dai, W.-Y. Wong. Langmuir-blodgett films of hexamolybdate and naphthylamine prepared by two different approaches: synthesis, characterization, and materials properties. *Eur. J. Inorg. Chem.* 2012 (2012) 684–694. https://doi.org/10.1002/ejic.201100972.

[61] J. Hao, L. Ruhlmann, Y. Zhu, Q. Li, Y. Wei. Naphthylimido-substituted hexamolybdate: preparation, crystal structures, solvent effects, and optical properties of three polymorphs. *Inorg. Chem.* 46 (2007) 4960–4967. https://doi.org/10.1021/ic070064b.

[62] J. Gao, X. Liu, Y. Liu, L. Yu, Y. Feng, H. Chen, Y. Li, G. Rakesh, C. H. A. Huan, T. C. Sum, Y. Zhao, Q. Zhang. Experimental and theoretical studies on pyrene-grafted polyoxometalate hybrid. *Dalton Trans.* 41 (2012) 12185–12191. https://doi.org/10.1039/c2dt31474f.

[63] I. Bar-Nahum, K. V. Narasimhulu, L. Weiner, R. Neumann. Phenanthroline−polyoxometalate hybrid compounds and the observation of intramolecular charge transfer. *Inorg. Chem.* 44 (2005) 4900–4902. https://doi.org /10.1021/ic050473c.

[64] S. She, S. Bian, R. Huo, K. Chen, Z. Huang, J. Zhang, J. Hao, Y. Wei. Degradable organically-derivatized polyoxometalate with enhanced activity against glioblastoma cell line. *Sci. Rep.* 6 (2016) 33529. https://doi.org/10.1038/srep33529.

[65] S. Xue, A. Chai, Z. Cai, Y. Wei, C. Xiang, W. Bian, J. Shen. A new class of functionalized polyoxometalates: synthesis, structure and preliminary antitumor activity studies of three arylimido substituted hexamolybdates bearing a strong electron-withdrawing nitro group. $(Bu_4N)_2[Mo_6O_{18}(\equiv NAr)]$ (Ar = $3\text{-}NO_2\text{-}C_6H_4$, $2\text{-}CH_3\text{-}4\text{-}NO_2\text{-}C_6H_3$, $2\text{-}CH_3\text{-}5\text{-}NO_2\text{-}C_6H_3$). *Dalton Trans.* (2008) 4770–4775. https://doi.org/10.1039/b719388b.

[66] Q. Li, Y. Wei, J. Hao, Y. Zhu, L. Wang. Unexpected C=C bond formation via doubly dehydrogenative coupling of two saturated sp^3 C−H bonds activated with a polymolybdate. *J. Am. Chem. Soc.* 129 (2007) 5810–5811. https://doi.org/10.1021/ja070600z.

[67] C. Lv, J. Zhang, J. Hao, L. Liu, Y. Wei. Syntheses and structural characterizations of di-substituted alkylimido hexamolybdates: an insight on bi-alkylimido functionalization. *Dalton Trans.* 41 (2012) 10065. https://doi.org/10.1039/c2dt30471f.

[68] Q. Li, P. Wu, Y. Wei, Y. Wang, P. Wang, H. Guo. Synthesis, structure and supramolecular assembly in the crystalline state of a bifunctionalized arylimido derivative of hexamolybdate. *Inorg. Chem. Commun.* 7 (2004) 524–527. https://doi.org/10.1016/j.inoche.2004.01.016.

[69] S. Xue, S. Ke, L. Yan, Z. Cai, Y. Wei. A trifluoromethyl substituted organoimido derivative of the hexametalate cluster: Synthesis, crystal structure and bioactivity of $[Mo_6O_{17}(NAr)_2]^{2-}$ (Ar = *o*-$CF_3C_6H_4$). *J. Inorg. Biochem.* 99 (2005) 2276–2281. https://doi.org/10.1016/j.jinorgbio.2005.08.006.

[70] Y. Xia, P. Wu, Y. Wei, Y. Wang, H. Guo. Synthesis, crystal structure, and optical properties of a polyoxometalate-based inorganic−organic hybrid solid, $(n\text{-}Bu_4N)_2[Mo_6O_{17}(\equiv NAr)_2]$ (Ar = *o*-$CH_3OC_6H_4$). *Cryst. Growth Des.* 6 (2006) 253–257. https://doi.org/10.1021/cg0503797.

[71] L. Xu, M. Lu, B. Xu, Y. Wei, Z. Peng, D. R. Powell. Towards main-chain-polyoxometalate-containing hybrid polymers: a highly efficient approach to bifunctionalized organoimido derivatives of hexamolybdates. *Angew. Chem. Int. Ed.* 41 (2002) 4129–4132. https://doi.org/10.1002/1521-3773(20021104)41:21<4129::AID-ANIE4129>3.0.CO;2-R.

[72] Y. Qiu, L. Xu, G. Gao, W. Wang, F. Li. A new arylimido derivative of polyoxometalate $(Bu_4N)_2[Mo_6O_{17}(NAr)_2]$ [Ar = 2,6-$(CH_3)_2C_6H_3$]: Synthesis, structure and physicochemical properties. *Inorg. Chim. Acta* 359 (2006) 451–458. https://doi.org/10.1016/j.ica.2005.07.020.

[73] Y. Shi, G. Xue, H. Hu, F. Fu, J. Wang. Synthesis and characterization of the organoimidopolyoxomolybdates $[n\text{-}Bu_4N]_2[Mo_6O_{17}(\equiv NAr)_2]$ (Ar = 2-ethyl-6-methylphenyl and 2-isopropyl-6-methylphenyl). *J. Coord. Chem.* 59 (2006) 1739–1747. https://doi.org/10.1080/00958970500537978.

[74] J. B. Strong, R. Ostrander, A. L. Rheingold, E. A. Maatta. Ensheathing a polyoxometalate: convenient systematic introduction of organoimido ligands at terminal oxo sites in $[Mo_6O_{19}]^{2-}$. *J. Am. Chem. Soc.* 116 (1994) 3601–3602. https://doi.org/10.1021/ja00087a057.

[75] M. Lu, B. Xie, J. Kang, F.-C. Chen, Y. Yang, Z. Peng. Synthesis of main-chain polyoxometalate-containing hybrid polymers and their applications in photovoltaic Cells. *Chem. Mater.* 17 (2005) 402–408. https://doi.org/10.1021/cm049003r.

[76] J. Kang, B. Xu, Z. Peng, X. Zhu, Y. Wei, D. R. Powell. Molecular and polymeric hybrids based on covalently linked polyoxometalates and transition-metal complexes. *Angew. Chem. Int. Ed.* 44 (2005) 6902–6905. https://doi.org/10.1002/anie.200501924.

[77] L. Wang, P. Yin, J. Zhang, J. Hao, C. Lv, F. Xiao, Y. Wei. χ-Octamolybdate $[Mo^{V}_4Mo^{VI}_4O_{24}]^{4-}$: An unusual small polyoxometalate in partially reduced form from nonaqueous solvent reduction. *Chem. Eur. J.* 17 (2011) 4796–4801. https://doi.org/10.1002/chem.201002154.

[78] C. Lv, R. N. N. Khan, J. Zhang, J. Hu, J. Hao, Y. Wei. Bifunctionalization of polyoxometalates with two different organoimido ligands. *Chem. Eur. J.* 19 (2013) 1174–1178. https://doi.org/10.1002/chem.201203204.

[79] W. Clegg, R. J. Errington, K. A. Fraser, S. A. Holmes, A. Schäfer, Functionalisation of $[Mo_6O_{19}]^{2-}$ with aromatic amines: synthesis and structure of a hexamolybdate building block with linear difunctionality. *Chem. Commun.* 4 (1995) 455–456. https://doi.org/10.1039/C39950000455.

[80] Y. Xia, Y. Wei, Y. Wang, H. Guo. A kinetically controlled trans bifunctionalized organoimido derivative of the Lindqvist-type hexamolybdate: synthesis, spectroscopic characterization, and crystal structure of $(n\text{-}Bu_4N)_2\{trans\text{-}[Mo_6O_{17}(NAr)_2]\}$ (Ar = 2,6-dimethylphenyl). *Inorg. Chem.* 44 (2005) 9823–9828. https://doi.org/10.1021/ic051319t.

[81] C. Qin, X. Wang, L. Xu, Y. Wei. A linear bifunctionalized organoimido derivative of hexamolybdate: Convenient synthesis and crystal structure. *Inorg. Chem. Commun.* 8 (2005) 751–754. https://doi.org/10.1016/j.inoche.2005.04.015.

[82] J. Hao, Y. Xia, L. Wang, L. Ruhlmann, Y. Zhu, Q. Li, P. Yin, Y. Wei, H. Guo. Unprecedented replacement of bridging oxygen atoms in polyoxometalates with organic imido ligands. *Angew. Chem. Int. Ed.* 120 (2008) 2666–2670. https://doi.org/10.1002/anie.200704546.

[83] J. B. Strong, B. S. Haggerty, A. L. Rheingold, E. A. Maatta. A superoctahedral complex derived from a polyoxometalate: the hexakis(arylimido)hexamolybdate anion $[Mo_6(NAr)_6O_{13}H]^{-}$. *Chem. Commun.* 12 (1997) 1137–1138. https://doi.org/10.1039/a701782k.

[84] Y. Huang, J. Zhang, H. Jia, J. Hao, Y. Wei. CSD crystal structure (2014).

[85] Y. Wei, M. Lu, C. F. Cheung, C. L. Barnes, Z. Peng. Functionalization of $[MoW_5O_{19}]^{2-}$ with aromatic amines: synthesis of the first arylimido derivatives of mixed-metal polyoxometalates. *Inorg. Chem.* 40 (2001) 5489–5490. https://doi.org/10.1021/ic0155683.

[86] J. L. Stark, A. L. Rheingold, E. A. Maatta. Polyoxometalate clusters as building blocks: preparation and structure of bis(hexamolybdate) complexes covalently bridged by organodiimido ligands. *J. Chem. Soc., Chem. Commun.* 11 (1995) 1165–1166. https://doi.org/10.1039/c39950001165.

[87] F. Xiao, M. F. Misdrahi, J. Zhang, P. Yin, J. Hao, C. Lv, Z. Xiao, T. Liu, Y. Wei. Buildup of amphiphilic molecular bola from organic-inorganic hybrid polyoxometalates and their vesicle-like supramolecular assembly. *Chem. Eur. J.* 17 (2011) 12006–12009. https://doi.org/10.1002/chem.201101966.

[88] C. Ritchie, G. Bryant. Microwave assisted synthesis of a mono organoimido functionalized Anderson polyoxometalate. *Dalton Trans.* 44 (2015) 20826–20829. https://doi.org/10.1039/C5DT04000K.

[89] J. Zhang, J. Hao, Y. Wei, F. Xiao, P. Yin, L. Wang. Nanoscale chiral rod-like molecular triads assembled from achiral polyoxometalates. *J. Am. Chem. Soc.* 132 (2010) 14–15. https://doi.org/10.1021/ja907535g.

[90] J. Zhang, P. Yin, J. Hao, F. Xiao, L. Chen, Y. Wei. Synthesis and assembly of a difunctional core POM cluster with two appended POM cluster caps. *Chem. Eur. J.* 18 (2012) 13596–13599. https://doi.org/10.1002/chem.201201098.

[91] A. Bayaguud, J. Zhang, R. N. N. Khan, J. Hao, Y. Wei. A redox active triad nanorod constructed from covalently interlinked organo-hexametalates. C*hem. Commun.* 50 (2014) 13150–13152. https://doi.org/10.1039/C4CC06297C.

[92] T. R. Mohs, G. P. A. Yap, A. L. Rheingold, E. A. Maatta. An organoimido derivative of the hexatungstate cluster: preparation and structure of $[W_6O_{18}(NAr)]^{2-}$ (Ar = 2,6-(i-Pr)$_2C_6H_3$). *Inorg. Chem*. 34 (1995) 9–10. https://doi.org/10.1021/ic00105a005.

[93] J. C. Duhacek, D. C. Duncan. Phenylimido functionalization of α-$[PW_{12}O_{40}]^{3-}$. *Inorg. Chem.* 46 (2007) 7253–7255. https://doi.org/10.1021/ic701024c.

[94] H. Kang, J. Zubieta. Co-ordination complexes of polyoxomolybdates with a hexanuclear core: synthesis and structural characterization of $(NBu^n_4)_2[Mo_6O_{18}(NNMePh)]$. *J. Chem. Soc., Chem. Commun.* 17 (1988) 1192–1193. https://doi.org/10.1039/c39880001192.

[95] S. Bank, S. Liu, S. N. Shaikh, X. Sun, J. Zubieta, P. D. Ellis. ^{95}Mo NMR Studies of (Aryldiazenido)- and (Organohydrazido)molybdates. Crystal and molecular structure of $[n\text{-}Bu_4N]_3[Mo_6O_{18}(NNC_6F_5)]$. *Inorg. Chem.* 27 (1988) 3535–3543. https://doi.org/10.1021/ic00293a021.

[96] T.-C. Hsieh, J. A. Zubieta. Synthesis and characterization of oxomolybdate clusters containing coordinatively bound organo-diazenido units: the crystal and molecular structure of the hexanuclear diazenido-oxomolybdate, $(NBu^n_4)_3[Mo_6O_{18}(N_2C_6H_5)]$. *Polyhedron 5* (1986) 1655–1657. https://doi.org/10.1016/S0277-5387(00)84576-3.

[97] C. Bustos, B. Hasenknopf, R. Thouvenot, J. Vaissermann, A. Proust, P. Gouzerh. Lindqvist-type (aryldiazenido)polyoxomolybdates − synthesis, and structural and spectroscopic characterization of compounds of the type $(nBu_4N)_3[Mo_6O_{18}(N_2Ar)]$. *Eur. J. Inorg. Chem.* 2003 (2003) 2757–2766. https://doi.org/10.1002/ejic.200300112.

[98] K.-B. Yu, H.-L. Li, X.-Z. You. Jiegou Huaxue 11 (1992) 248.

[99] C. Bustos, D. M.-L. Carey, K. Boubekeur, R. Thouvenot, A. Proust, P. Gouzerh. Aryldiazenido derivatives: A new entry to the functionalization of Keggin polyoxometalates. *Inorg. Chim. Acta* 363 (2010) 4262–4268. https://doi.org/10.1016/j.ica.2010.06.044.

[100] J. J. Baldoví, Y. Duan, C. Bustos, S. Cardona-Serra, P. Gouzerh, R. Villanneau, G. Gontard, J. M. Clemente-Juan, A. Gaita-Ariño, C. Giménez-Saiz, A. Proust, E. Coronado. Single ion magnets based on lanthanoid polyoxomolybdate complexes. *Dalton Trans.* 45 (2016) 16653–16660. https://doi.org/10.1039/C6DT02258H.

[101] L. Wang, P. Yin, J. Zhang, F. Xiao, Z. Fang, W. Fu, Y. Wei, S. Xue. An unprecedented class of benzoyldiazenido-functionalized polyoxometalates with enhanced antitumour activities. *Eur. J. Inorg. Chem.* 46 (2017) 5475–5484. https://doi.org/10.1002/ejic.201700990.

[102] H. Kwen, V. G. Young, Jr., E. A. Maatta. A Diazoalkane Derivative of a polyoxometalate: preparation and structure of $[Mo_6O_{18}(NNC(C_6H_4OCH_3)CH_3)]^{2-}$. *Angew. Chem. Int. Ed.* 38 (1999) 1145–1146. https://doi.org/10.1002/(SICI)1521-3773(19990419)38:8<1145::AID-ANIE1145>3.0.CO;2-M.

[103] S. Gatard, S. Blanchard, B. Schollhorn, P. Gouzerh, A. Proust, K. Boubekeur. Electroactive benzothiazole hydrazones and their $[Mo_6O_{19}]^{2-}$ derivatives: Promising building blocks for conducting molecular materials. *Chem. Eur. J.* 16 (2010) 8390–8399. https://doi.org/10.1002/chem.201000427.

[104] A. Proust, R. Thouvenot, F. Robert, P. Gouzerh. Molybdenum oxo nitrosyl complexes. 2. ^{95}Mo NMR studies of defect and complete Lindqvist-type derivatives. Crystal and molecular structure of (*n*-$Bu_4N)_2[Mo_6O_{17}(OCH_3)(NO)]$. *Inorg. Chem.* 32 (1993) 5299–5304. https://doi.org/10.1021/ic00075a057.

[105] P. Gouzerh, Y. Jeannin, A. Proust, F. Robert. Two novel polyoxomolybdates containing the $(MoNO)^{3\oplus}$ unit: $[Mo_5Na(NO)O_{13}(OCH_3)_4]^{2\ominus}$ and $[Mo_6(NO)O_{18}]^{3\ominus}$. *Angew. Chem. Int. Ed.* 28 (1989) 1363–1364. https://doi.org/10.1002/anie.198913631.

[106] A. Proust, P. Gouzerh, F. Robert. Molybdenum oxo nitrosyl complexes. 1. Defect Lindqvist compounds of the type $[Mo_5O_{13}(OR)_4(NO)]^{3-}$ (R = CH_3, C_2H_5) Solid-state interactions with alkali-metal cations. *Inorg. Chem.* 32 (1993) 5291–5298. https://doi.org/10.1021/ic00075a056.

[107] R. Villanneau, A. Proust, F. Robert, P. Gouzerh. Coordination chemistry of the soluble metal oxide analogue $[Mo_5O_{13}(OCH_3)_4(NO)]^{3-}$ with manganese carbonyl species. *Chem. Eur. J.* 9 (2003) 1982–1990. https://doi.org/10.1002/chem.200204547.

[108] S. She, C. Gao, K. Chen, A. Bayaguud, Y. Huang, B.-W. Wang, S. Gao, Y. Wei. A series of Weakley-type polyoxomolybdates: Synthesis, characterization, and magnetic properties by a combined experimental and theoretical approach. *Inorg. Chem.* 57 (2018) 963–969. https://doi.org/10.1021/acs.inorgchem.7b01971.

[109] R. Villanneau, A. Proust, F. Robert, P. Gouzerh. Co-ordination chemistry of lacunary Lindqvist-type polyoxometalates: cubic *vs.* square-antiprismatic co-ordination. *J. Chem. Soc., Dalton Trans.* 4 (1999) 421–426. https://doi.org/10.1039/a807942k.

[110] R. Villanneau, A. Proust, F. Robert. Synthesis and characterization of $[NBu_4]_4[Ag_2\{Mo_5O_{13}(OMe)_4(NO)\}_2]$, a novel polyoxomolybdate complex with a short $Ag^I \cdots Ag^I$ distance. *Chem. Commun.* 13 (1998) 1491–1492. https://doi.org/10.1039/a802548g.

[111] R. Villanneau, A. Proust, F. Robert, P. Veillet, P. Gouzerh. Synthesis, structure, and magnetic properties of $(n\text{-}Bu_4N)_2[\{Ni(MeOH)_2\}_2\{Mo(NO)\}_2(\mu_3\text{-}OH)_2(\mu\text{-}OMe)_4\{Mo_5O_{13}(OMe)_4(NO)\}_2]$, a new type of polyoxometalate incorporating a rhomb-like cluster. *Inorg. Chem.* 38 (1999) 4981–4985. https://doi.org/10.1021/ic990480z.

[112] A. Proust, M. Fournier, R. Thouvenot, P. Gouzerh. Synthesis and characterization of Keggin derivatives containing an $[Mo(NO)]^{3+}$ unit: $(n\text{-}Bu_4N)_4[PM_{11}O_{39}\{Mo(NO)\}]$ (M = Mo, W). *Inorg. Chim. Acta* 215 (1994) 61–66. https://doi.org/10.1016/0020-1693(93)03666-X.

[113] C. A. Ohlin, E. M. Villa, J. C. Fettinger, W. H. Casey. Distinctly different reactivities of two similar polyoxoniobates with hydrogen peroxide. *Angew. Chem. Int. Ed.* 47 (2008) 8251–8254. https://doi.org/10.1002/anie.200803688.

[114] M. K. Harrup, G.-S. Kim, H. Zeng, R. P. Johnson, D. VanDerveer, C. L. Hill. Triniobium polytungstophosphates. Syntheses, structures, clarification of isomerism and reactivity in the presence of H_2O_2. *Inorg. Chem.* 37 (1998) 5550-5556. https://doi.org/10.1021/ic980467z.

[115] G.-S. Kim, D. A. Judd, C. L. Hill, R. F. Schinazi. Synthesis, characterization and biological activity of a new potent class of Anti-HIV agents, the peroxoniobium-substituted heteropolytungstates. *J. Med. Chem.* 37 (1994) 816-820. https://doi.org/10.1021/jm00032a016.

[116] M. W. Droege, R. G. Finke. A novel triperoxyniobium-containing polyoxoanion, $SiW_9(NbO_2)_3O_{37}^{7-}$: synthesis, characterization, catalytic allylic epoxidations with H_2O_2 and preliminary kinetic studies. *J. Mol. Catal.* 69 (1991) 323-338. https://doi.org/10.1016/0304-5102(91)80113-H.

[117] D. A. Judd, J. H. Nettles, N. Nevins, J. P. Snyder, D. C. Liotta, J. Tang, J. Ermolieff, R. F. Schinazi, C. L. Hill. Polyoxometalate HIV-1 protease inhibitors. A new mode of protease inhibition. *J. Am. Chem. Soc.* 123 (2001) 886-897. https://doi.org/10.1021/ja001809e.

[118] D. A. Judd, Q. Chen, C. F. Campana, C. L. Hill. Synthesis, Solution and solid state structures, and aqueous chemistry of an unstable polyperoxo polyoxometalate: $[P_2W_{12}(NbO_2)_6O_{56}]^{12-}$. *J. Am. Chem. Soc.* 119 (1997) 5461–5462. https://doi.org/10.1021/ja970329t.

[119] M. J. Deery, O. W. Howarth, K. R. Jennings. Application of electrospray ionisation mass spectrometry to the study of dilute aqueous oligomeric anions and their reactions. *J. Chem. Soc., Dalton Trans.* 24 (1997) 4783–4788. https://doi.org/10.1039/A706560D.

[120] J. Server-Carrió, J. Bas-Serra, M. E. González-Núñez, A. García-Gastaldi, G. B. Jameson, L. C. W. Baker, R. Acerete. Synthesis, characterization, and catalysis of β_3-$[(Co^{II}O_4)W_{11}O_{31}(O_2)_4]^{10-}$, the first Keggin-based true heteropoly dioxygen (peroxo) anion. Spectroscopic (ESR, IR) evidence for the formation of superoxo polytungstates. *J. Am. Chem. Soc.* 121 (1999) 977-984. https://doi.org/10.1021/ja9804969.

[121] W. G. Klemperer, C. Schwartz. Synthesis and characterization of the polyoxothioanions $TaW_5O_{18}S^{3-}$ and $NbW_5O_{18}S^{3-}$. *Inorg. Chem.* 24 (1985) 4459–4461. https://doi.org/10.1021/ic00220a008.

[122] E. Cadot, V. Béreau, F. Sécheresse. Synthesis and characterization of the polyoxothioanion α-$[PW_{11}NbSO_{39}]^{4-}$ derived from the Keggin structure. *Inorg. Chim. Acta* 239 (1995) 39–42. https://doi.org/10.1016/0020-1693(95)04711-5.

[123] E. Cadot, V. Béreau, F. Sécheresse. Sulfur in Keggin heteropolyanions. Syntheses and multinuclear NMR characterizations (^{31}P, ^{183}W and ^{95}Mo) of γ-$[PW_{10}M_2S_2O_{38}]^{5-}$ (M = Mo, W). *Inorg. Chim. Acta* 252 (1996) 101–106. https://doi.org/10.1016/S0020-1693(96)05302-9.

[124] E. Cadot, V. Béreau, B. Marg, S. Halut, F. Sécheresse. Syntheses and characterization of γ-$[SiW_{10}M_2S_2O_{38}]^{6-}$ (M = Mo^V , W^V). Two Keggin oxothio heteropolyanions with a metal−metal bond. *Inorg. Chem.* 35 (1996) 3099–3106. https://doi.org/10.1021/ic951054q.

[125] V. Béreau, E. Cadot, H. Bögge, A. Müller, F. Sécheresse. Addition of $\{M_2S_2O_2\}^{2+}$, M = Mo, W, to A-α-$[PW_9O_{34}]^{9-}$. Synthesis and structural characterizations in the solid state and in solution. *Inorg. Chem.* 38 (1999) 5803–5808. https://doi.org/10.1021/ic990666y.

[126] F. Sécheresse, E. Cadot. Sulfur-containing Keggin anions and cyclic polyoxothioanions, in: Polyoxometalate Mol. Sci., Springer Netherlands, Dordrecht, 2003: pp. 253–271. https://doi.org/10.1007/978-94-010-0091-8_8.

[127] V. S. Korenev, P. A. Abramov, C. Vicent, D. A. Mainichev, S. Floquet, E. Cadot, M. N. Sokolov, V. P. Fedin. Trapping $\{BW_{12}\}_2$ tungstoborate: synthesis and crystal structure of hybrid $[\{(H_2BW_{12}O_{42})_2O\}\{Mo_6O_6S_6(OH)_4(H_2O)_2\}]^{14-}$ anion. *Dalton Trans*. 41 (2012) 14484–14486. https://doi.org/10.1039/c2dt31512b.

[128] E. Cadot, A. Dolbecq, B. Salignac, F. Sécheresse. Self-condensation of $[Mo^V{}_2O_2S_2]^{2+}$ with phosphate or arsenate ions by acid-base processes in aqueous solution: syntheses, crystal structures, and reactivity of $[(HXO_4)_4Mo_6S_6O_6(OH)_3]^{5-}$. X = P, As, *Chem. Eur. J.* 5 (1999) 2396–2403. https://doi.org/10.1002/(SICI)1521-3765(19990802)5:8<2396::AID-CHEM2396>3.0.CO;2-C.

[129] A. Dolbecq, E. Cadot, D. Eisner, F. Sécheresse. Regioselective S/O substitutions in heteropolyoxothioanions: ^{31}P NMR study and X-ray crystal structure of the half-substituted anion $[(HPO_4)_4Mo_6S_3O_9(OH)_3]^{5-}$. *Inorg. Chim. Acta* 300–302 (2000) 151–157. https://doi.org/10.1016/S0020-1693(99)00458-2.

[130] B. Salignac, S. Riedel, A. Dolbecq, F. Sécheresse, E. Cadot. “Wheeling Templates” in molecular oxothiomolybdate rings: syntheses, structures, and dynamics. *J. Am. Chem. Soc.* 122 (2000) 10381–10389. https://doi.org/10.1021/ja001878t.

[131] A. Dolbecq, E. Cadot, F. Sécheresse. $[Mo_9S_8O_{12}(OH)_8(H_2O)_2]^{2-}$: a novel polyoxothiomolybdate with a Mo^{VI} octahedron encapsulated in a reduced Mo^{V} cyclic octanuclear core. *Chem. Commun.* 12 (1998) 2293–2294. https://doi.org/10.1039/a805453c.

[132] A. Dolbecq, C. du Peloux, A.-L. Auberty, S. A. Mason, P. Barboux, J. Marrot, E. Cadot, F. Sécheresse. Synthesis, X-ray and Neutron Diffraction Characterization, and Ionic Conduction Properties of a New Oxothiomolybdate $Li_3[Mo_8S_8O_8(OH)_8\{HWO_5(H_2O)\}]\cdot 18\ H_2O$. *Chem. Eur. J.* 8 (2002) 349–356. https://doi.org/10.1002/1521-3765(20020118)8:2<349::AID-CHEM349>3.0.CO;2-5.

[133] E. Cadot, A. Dolbecq, B. Salignac, F. Sécheresse. From molecular rings to the 3-D solid: ionization of the neutral $[Mo_{10}S_{10}O_{10}(OH)_{10}(H_2O)_5]$ molecular ring for the building blocks strategy. *J. Phys. Chem. Solids* 62 (2001) 1533–1543. https://doi.org/10.1016/S0022-3697(01)00070-1.

[134] E. Cadot, B. Salignac, J. Marrot, A. Dolbecq, F. Sécheresse. $[Mo_{10}S_{10}O_{10}(OH)_{10}(H_2O)_5]$: a novel decameric molecular ring showing supramolecular properties. *Chem. Commun.* 10 (2000) 261–262. https://doi.org/10.1039/a909024j.

[135] E. Cadot, B. Salignac, T. Loiseau, A. Dolbecq, F. Sécheresse. Syntheses and ^{31}P NMR studies of cyclic oxothiomolybdate(V) molecular rings: exchange properties and crystal structures of the monophosphate decamer $[(H_2PO_4)Mo_{10}S_{10}O_{10}(OH)_{11}(H_2O)_2]^{2-}$ and the diphosphate dodecamer $[(HPO_4)_2Mo_{12}S_{12}O_{12}(OH)_{12}(H_2O)_2]^{4-}$. *Chem. Eur. J.* 5 (1999) 3390–3398. https://doi.org/10.1002/(SICI)1521-3765(19991105)5:11<3390::AID-CHEM3390>3.0.CO;2-B.

[136] H.-Y. Zang, A. R. de la Oliva, H. N. Miras, D.-L. Long, R. T. McBurney, L. Cronin. Discovery of gigantic molecular nanostructures using a flow reaction array as a search engine. *Nat. Commun.* 5 (2014) 3715. https://doi.org/10.1038/ncomms4715.

[137] E. Cadot, B. Salignac, S. Halut, F. Sécheresse. $[Mo_{12}S_{12}O_{12}(OH)_{12}(H_2O)_6]$: A cyclic molecular cluster based on the $[Mo_2S_2O_2]^{2+}$ building block. *Angew. Chem. Int. Ed.* 37 (1998) 611–613. https://doi.org/10.1002/(SICI)1521-3773(19980316)37:5<611::AID-ANIE611>3.0.CO;2-O.

[138] E. Cadot, J. Marrot, F. Sécheresse. $[W_{16}S_{16}O_{16}(OH)_{16}(H_2O)_4(C_5H_6O_4)_2]^{4-}$: A Flexible, Pillared oxothiotungstate wheel, *Angew. Chem. Int. Ed.* 40 (2001) 774–777. https://doi.org/10.1002/1521-3773(20010216)40:4<774::AID-ANIE7740>3.0.CO;2-L.

[139] J. W. Purcell, H. N. Miras, D.-L. Long, P. Markopoulou, L. Cronin. Tellurite-squarate driven assembly of a new family of nanoscale clusters based on $(Mo_2O_2S_2)^{2+}$. *Chem. Eur. J.* 23 (2017) 9683–9689. https://doi.org/10.1002/chem.201701920.

[140] M. I. Khan, Q. Chen, H. Hope, S. Parkin, C. J. O'Connor, J. Zubieta. Hydrothermal synthesis and characterization of hexavanadium polyoxo alkoxide anion clusters: crystal structures of the vanadium(IV) species $Ba[V_6O_7(OH)_3\{(OCH_2)_3CCH_3\}_3]\cdot 3H_2O$ and $Na_2[V_6O_7\{(OCH_2)_3CCH_2CH_3\}_4]$, of the mixed-valence complex $(Me_3NH)[V^{IV}{}_5V^{V}O_7(OH)_3\{(OCH_2)_3CCH_3\}_3]$, and of the fluoro derivative $Na[V_6O_6F(OH)_3\{(OCH_2)_3CCH_3\}_3]\cdot 3H_2O$. *Inorg. Chem.* 32 (1993) 2929–2937. https://doi.org/10.1021/ic00065a022.

[141] M. Nicolaou, C. Drouza, A. D. Keramidas. Controlled one pot synthesis of polyoxofluorovanadate molecular hybrids exhibiting peroxidase like activity. *New J. Chem.* 43 (2019) 17595–17602. https://doi.org/10.1039/C9NJ01999E.

[142] F. Chauveau, P. Doppelt, J. Lefebvre. Mechanism of fluoride loss from fluoropolytungstates possessing the Keggin structure. *Polyhedron* 1 (1982) 263–267. https://doi.org/10.1016/S0277-5387(00)87163-6.

[143] S. H. Wasfi, W. L. Johnson, S. McMasters. Preparation and characterization of $[Fe(III)W_{11}O_{39}FH_4]^{6-}$, a new member of the 1:11 heteropolyoxofluorotungstate anions. *Synth. React. Inorg. Met. Chem.* 31 (2001) 391–403. https://doi.org/10.1081/SIM-100002227.

[144] S. H. Wasfi, A. L. Rheingold, B. S. Haggerty. Preparation and characterization of two novel heteropolyfluorooxotungstate anions $[NaCoW_{11}O_{43}FH_{12}]^{6-}$ and $[NaNiW_{11}O_{43}FH_{12}]^{6-}$. *Inorg. Chim. Acta* 282 (1998) 136–141. https://doi.org/10.1016/S0020-1693(98)00158-3.

[145] S. H. Wasfi, J. C. Johnson. The preparation and characterization of two new heteropolyoxofluorotungstate anions $[CuW_{11}O_{38}F_2H_6]^{4-}$ and $[MnW_{11}O_{38}F_2H_6]^{4-}$. *Synth. React. Inorg. Met. Chem.* 26 (1996) 1073–1086. https://doi.org/10.1080/00945719608004352.

[146] S. H. Wasfi, W. L. Johnson, D. L. Martin. The preparation and characterization of a new heteropolyoxofluorotungstate anion $[CoW_{11}O_{38}F_2H_4]^{6-}$. *Synth. React. Inorg. Met. Chem.* 27 (1997) 401–417. https://doi.org/10.1080/00945719708000197.

[147] S. H. Wasfi, J. C. Johnson. The preparation and characterization of a new heteropolyoxofluorotungstate anion $[MnW_{11}O_{37}F_3H]^{7-}$. *Synth. React. Inorg. Met. Chem.* 26 (1996) 1339–1349. https://doi.org/10.1080/00945719608005128.

[148] S. H. Wasfi, W. L. Johnson, D. L. Martin. The preparation and characterization of the fluorinated dinuclear mixed-valence heteropolyoxotungstate anion $[Fe^{3+}ZnW^{6+}{}_{10}W^{5+}O_{36}F_4]^{6-}$. *Synth. React. Inorg. Met. Chem.* 27 (1997) 535–549. https://doi.org/10.1080/00945719708000208.

[149] S. H. Wasfi, S. Tribbitt, F. Divita. The preparation and characterization of two new isomorphous binuclear heteropolytungstate anions. *Synth. React. Inorg. Met. Chem.* 23 (1993) 991–1010. https://doi.org/10.1080/15533179308016877.

[150] S. H. Wasfi, J. Johnson, D. Martin. The preparation and characterization of two new heteropolyoxofluorotungstate anions $[CuNiW_{11}O_{38}F_2H_4]^{4-}$ and $[NiMnW_{11}O_{38}F_2H_4]^{4-}$. *Synth. React. Inorg. Met. Chem.* 25 (1995) 1061–1076. https://doi.org/10.1080/15533179508218294.

[151] S. H. Wasfi, W. L. Johnson, D. L. Martin. The preparation and characterization of the fluorinated dinuclear heteropolyoxotungstate anion with loosely coordinated water molecule: $[(H_2O)CuO_5ZnO_4W_{11}O_{28}F_2]^{8-}$. *Synth. React. Inorg. Met. Chem.* 28 (1998) 223–244. https://doi.org/10.1080/00945719809351900.

[152] F. Chauveau, P. Doppelt, J. Lefebvre. Fluorotungstates of the metatungstate family: identification and properties of one compound of the 2-18 series. *Inorg. Chem.* 19 (1980) 2803–2806. https://doi.org/10.1021/ic50211a062.

[153] T. L. Jorris, M. Kozik, L. C. W. Baker. Reformulation of the hexafluorooctadecatungstate anion as a heteropoly sodate complex: $[H_2F_6NaW_{18}O_{56}]^{7-}$. *Inorg. Chem.* 29 (1990) 4584–4586. https://doi.org/10.1021/ic00347a052.

[154] R. Ben-Daniel, A. M. Khenkin, R. Neumann. The nickel-substituted quasi-Wells-Dawson-type polyfluoroxometalate, $[Ni^{II}(H_2O)H_2F_6NaW_{17}O_{55}]^{9-}$, as a uniquely active nickel-based catalyst for the activation of hydrogen peroxide and the epoxidation of alkenes and alkenols. *Chem. Eur. J.* 6 (2000) 3722–3728. https://doi.org/10.1002/1521-3765(20001016)6:20<3722::AID-CHEM3722>3.0.CO;2-8.

[155] S. H. Wasfi, C. E. Costello, A. L. Rheingold, B. S. Haggerty. Preparation and characterization of two new isomorphous heteropoly oxofluorotungstate anions $[CoW_{17}O_{56}F_6NaH_4]^{9-}$ and $[FeW_{17}O_{56}F_6NaH_4]^{8-}$. *Inorg. Chem.* 30 (1991) 1788–1792. https://doi.org/10.1021/ic00008a021.

[156] S. H. Wasfi, S. A. Tribbitt. The preparation and characterization of three new fluorotungstate anions having the Dawson structure $[Cu^{+}W_{17}O_{54}F_8NaH_4]^{8-}$, $[MgW_{17}O_{57}F_5NaH_6]^{8-}$, and $[Fe^{+3}W^{+5}W_{16}{}^{+6}O_{55}F_7NaH_4]^{8-}$. *Synth. React. Inorg. Met. Chem*. 24 (1994) 487–498. https://doi.org/10.1080/00945719408000126.

[157] S. H. Wasfi. The Preparation and characterization of a new heteropolytungstate anion $[CuW_{17}O_{57}F_5H_5Na]^{9-}$. *Synth. React. Inorg. Met. Chem.* 22 (1992) 663–672. https://doi.org/10.1080/15533179208020236.

[158] L. J. Batchelor, R. Shaw, S. J. Markey, M. Helliwell, E. J. L. McInnes. An all-vanadium(III) hexametalate Lindqvist structure and its chromium and iron analogues. *Chem. Eur. J.* 16 (2010) 5554–5557. https://doi.org/10.1002/chem.201000222.

[159] M. L. Maiola, B. E. Petel, W. W. Brennessel, E. M. Matson. Site-selective halogenation of mixed-valent vanadium oxide clusters. *Dalton Trans*. (2020), 16184–16192. https://doi.org/10.1039/D0DT01077D

[160] R. J. Errington, R. L. Wingad, W. Clegg, M. R. J. Elsegood. Direct bromination of Keggin fragments to give $[PW_9O_{28}Br_6]^{3-}$: a polyoxotungstate with a hexabrominated face. *Angew. Chem. Int. Ed.* 39 (2000) 3884–3886. https://doi.org/10.1002/1521-3773(20001103)39:21<3884::AID-ANIE3884>3.0.CO;2-M.

[161] P. L. Holland. Metal–dioxygen and metal–dinitrogen complexes: where are the electrons? *Dalton Trans.* 39 (2010) 5415. https://doi.org/10.1039/c001397h.

[162] V. S. Sergienko. Structural characteristics of peroxo complexes of group IV and V transition metals. Review. *Crystallogr. Rep.* 49 (2004) 907–929. https://doi.org/10.113
